# Supplementary material for: Improving mass spectrometry analysis of protein structures with arginine-selective chemical cross-linkers
Source: Nat Commun. 2019 Sep 2;10:3911. doi: 10.1038/s41467-019-11917-z (PMC6718413; doi:10.1038/s41467-019-11917-z)
Supplement: Supplementary file 1 — Supplementary Information [file 41467_2019_11917_MOESM1_ESM.docx]

**Supplementary Information**

**Improving Mass Spectrometry Analysis of Protein Structures with Arginine-Selective Chemical Cross-linkers**

Jones et al.

**Contents**

[**Supplementary Figures** 3](#_Toc15540269)

[Supplementary Figure 1. Theoretical assessment of lysine-lysine, arginine-arginine and lysine-arginine cross-linking 3](#_Toc15540270)

[Supplementary Figure 2. Structures of compounds screened for their reactivity to arginine 4](#_Toc15540271)

[Supplementary Figure 3. Calculation of second order rate constant for reaction of p-OMe-phenyl glyoxal with N-Ac-Arginine methyl ester. 5](#_Toc15540272)

[Supplementary Figure 4. Reaction of *p*-OMe phenyl glyoxal and N-Ac arginine methyl ester 6](#_Toc15540273)

[Supplementary Figure 5. Optimization of cross-linking conditions using BSA 7](#_Toc15540274)

[Supplementary Figure 6. SDS-PAGE gels of ArGO-cross-linked proteins 8](#_Toc15540275)

[Supplementary Figure 7. Euclidean distances and structural compatibility of ArGO cross-links 10](#_Toc15540276)

[Supplementary Figure 8. Sodium periodate treatment of ArGO-ligation reactions 11](#_Toc15540277)

[Supplementary Figure 9. Structures of ArGO analogues and number of BSA peptide pairs identified with each 12](#_Toc15540278)

[Supplementary Figure 10. Optimisation of cross-linking buffer and trypsin digestion for ArGO2 13](#_Toc15540279)

[Supplementary Figure 11. Structure modelling of CNG complex using Rosetta 3.5 with prepack protocol for pre-treatment. 14](#_Toc15540280)

[Supplementary Figure 12. Optimization of KArGO cross-linking conditions using BSA 15](#_Toc15540281)

[Supplementary Figure 13. KArGO CXMS analysis of ALPK1 N-terminal domain (NTD) 17](#_Toc15540282)

[Supplementary Tables 18](#_Toc15540283)

[Supplementary Table 1 18](#_Toc15540284)

[Supplementary Table 2 19](#_Toc15540285)

[Supplementary Table 3 20](#_Toc15540286)

[Supplementary Table 4 21](#_Toc15540287)

[Supplementary Note 23](#_Toc15540288)

[Supplementary Methods 75](#_Toc15540289)

[Mass Spectrometry 75](#_Toc15540290)

[Chemistry Experimental 75](#_Toc15540291)

[Supplementary References 77](#_Toc15540292)

**Supplementary Figures**

**
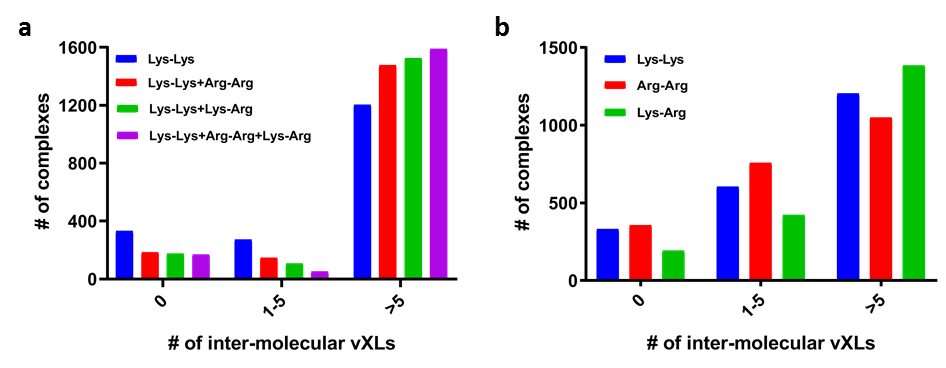
**

Supplementary Figure 1. Theoretical assessment of lysine-lysine, arginine-arginine and lysine-arginine cross-linking

Using Xwalk^1^, and following a procedure (Supplementary method) , the number of K-K, R-R, and K-R pairs not exceeding a Cα-Cα distance of 24 Å (the upper limit of DSS and BS^3^ cross-links) were calculated from 1808 PDB structures of proteins complexes. The number of protein complexes with 0, 1-5, or >5 virtual inter-molecular cross-links with combinations of cross-linkers **a)** or K-K, R-R, K-R cross-linkers alone **b)** are shown.

Supplementary Figure 2. Structures of compounds screened for their reactivity to arginine


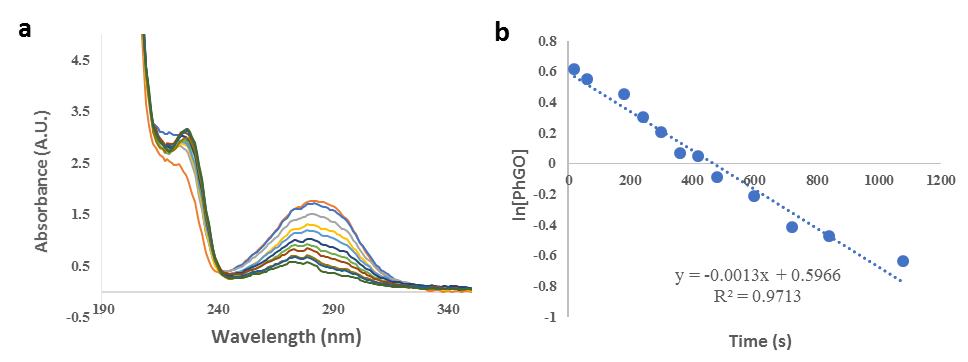


Supplementary Figure 3. Calculation of second order rate constant for reaction of p-OMe-phenyl glyoxal with N-Ac-Arginine methyl ester.

**a)** Calculation of second order rate constant for reaction of p-OMe-phenyl glyoxal with N-Ac-Arginine methyl ester. UV-vis spectroscopy was used to measure the pseudo-first order rate constant for the condensation of p-OMe-phenyl glyoxal (2.0 mM) and N-Ac-Arginine methyl ester (20 mM) in 100 mM pH 8 borate buffer:MeCN (3.75:1.25 mL), and the reaction mixture was stirred at room temperature. Samples (2 L) were taken every 60-120 s and analysed directly by Nano-drop UV. Reaction conversion was monitored by the decrease in p-OMe-phenyl glyoxal absorbance at 283 nm. The reaction was repeated in triplicate.

**b)** The pseudo-first order rate constant (k­obs) for the reaction was determined by plotting ln[p-OMe-PhGO] versus time (s), and analysis by linear regression. The average kobs = 1.3 x 10-3 s^-1. The second order rate constant was estimated by kobs/[Arginine]. As a result, the second order rate constant was 6.5 x 10-2 M^-1 s^-1 in 3:1 100 mM pH 8 borate buffer:MeCN.

Supplementary Figure 4. Reaction of *p*-OMe phenyl glyoxal and N-Ac arginine methyl ester

Reactant **1** is converted to intermediate **2** (MW = 394) and then to major product **3** (MW = 376). **2** is stabilized by borate through the formation of **4**. Compounds **2** and **3** were detected within 1 h. Longer reaction times (3 h) resulted in the formation of oxidation product **5** (MW = 374) and bis-glyoxal adduct **6** (MW = 540) whose structures are shown.


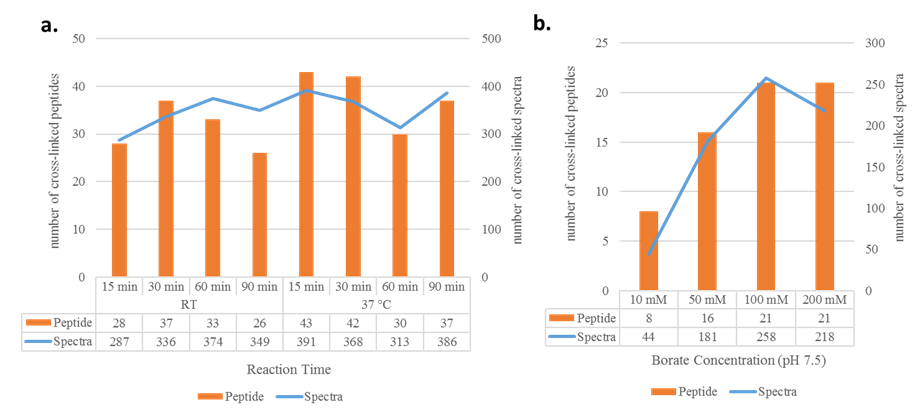


**
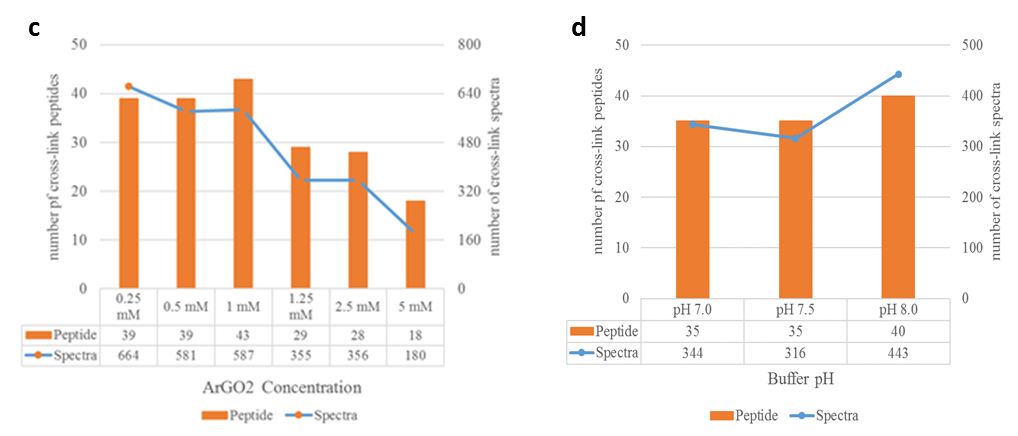
**

Supplementary Figure 5. Optimization of cross-linking conditions using BSA

**a)** Number of uniquely identified ArGO-linked peptide pairs (orange bars, peptide pairs cross-linked through the same pair of arginine residues are merged as one, same below) and their total spectral counts (blue line) from 15 – 90 min cross-linking reactions at RT or 37 °C.

**b-d)** Number of ArGO-linked peptide pairs (orange bars) and spectral counts (blue line) identified from sets of cross-linking reactions in which either the concentration of borate **b)** concentration of ArGO2 **c)**, or pH **d)** was varied. Detailed reaction conditions are described below the graphs.


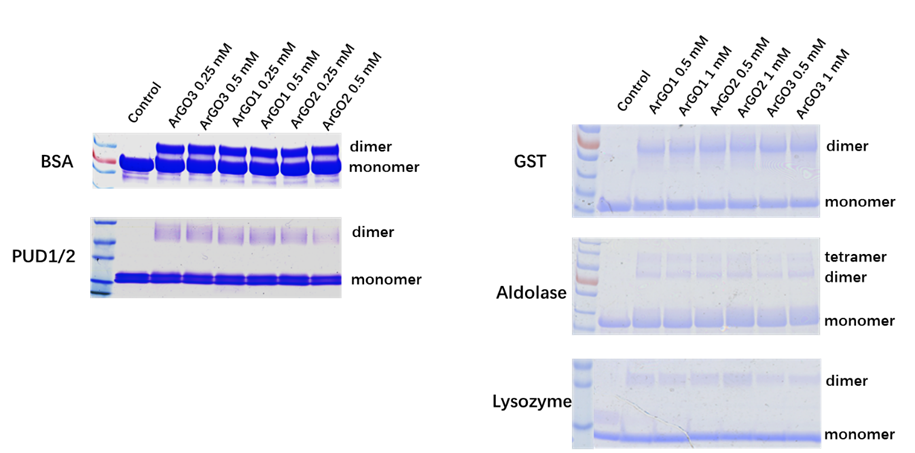


Supplementary Figure 6. SDS-PAGE gels of ArGO-cross-linked proteins

GST, Aldolase, lysozyme, PUD-1/2 and BSA were treated with ArGO1-3 at 0.5 or 1 mM for 1 h at RT. The uncropped gel figures are provided in Source Data file.

**a**


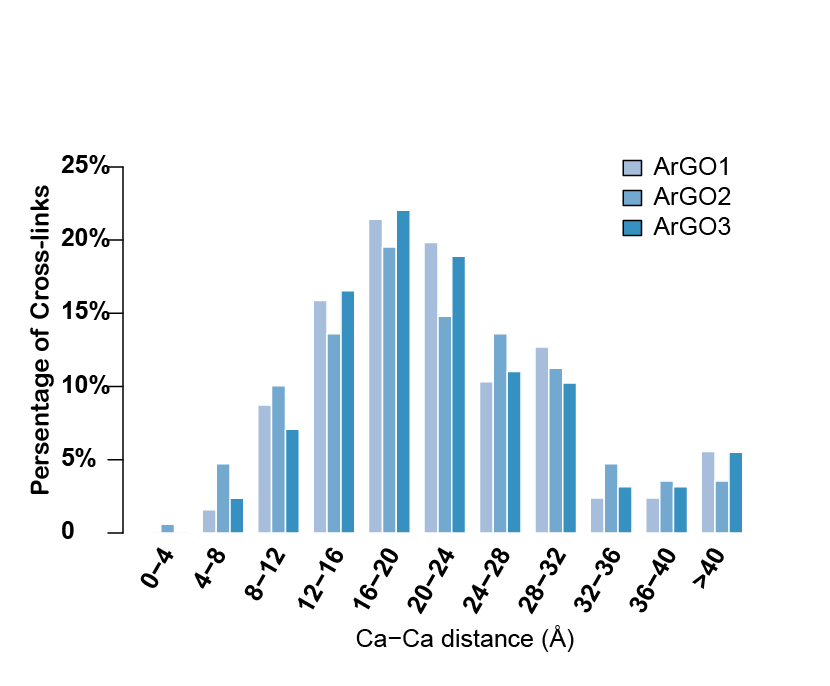


**b.**

**
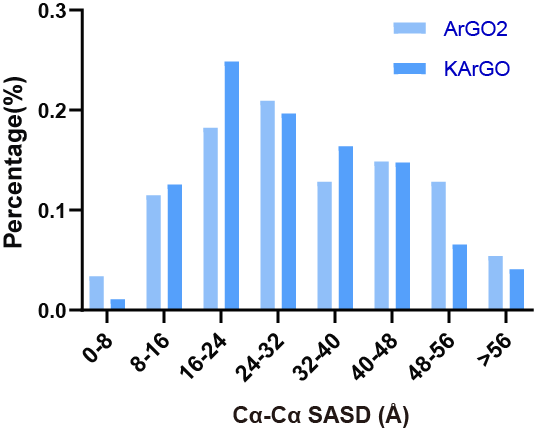
**

**c**

|  | ArGO1 | ArGO2 | ArGO3 |
| --- | --- | --- | --- |
| Max. Distance Restraint (Å) | 29.4 | 33.7 | 37.7 |
| Structural Compatibly (%) | 84.1 | 90.5 | 92.1 |

**d**

Theoretically cross-linkable and experimentally observed R-R or K-R pairs in six model proteins.

| **Euclidean Distance (Å)** | **0-4** | **4-8** | **8-12** | **12-16** | **16-20** | **20-24** | **24-28** | **28-32** | **32-36** | **36-40** | **>40** |
| --- | --- | --- | --- | --- | --- | --- | --- | --- | --- | --- | --- |
| Theoretical RR pairs | 0 | 13 | 47 | 87 | 132 | 159 | 156 | 128 | 118 | 118 |  |
| ArGO1 X-links | 0 | 2 | 11 | 22 | 25 | 25 | 14 | 15 | 3 | 3 | 6 |
| ArGO2 X-links | 1 | 8 | 17 | 23 | 33 | 25 | 23 | 19 | 8 | 6 | 6 |
| ArGO3 X-links | 0 | 3 | 9 | 23 | 27 | 23 | 15 | 12 | 4 | 4 | 7 |
| Theoretical KR pairs | 0 | 26 | 115 | 191 | 273 | 348 | 428 | 404 | 372 | 334 |  |
| KArGO X-links | 2 | 8 | 47 | 75 | 59 | 55 | 53 | 21 | 20 | 10 | 29 |

Supplementary Figure 7. Euclidean distances and structural compatibility of ArGO cross-links

**a)** Distribution of the distances of ArGO cross-links in six model proteins.

**b)** Distribution of the solvent accessible surface distance (SASD) of ArGO2 cross-links in six model proteins. Source data of the ED and SASD of ArGO2 cross-links are provided in Source Data file.

**c)** The maximum distance of two arginine residues cross-linkable by ArGO1-3 and the percentage of observed cross-links that fit within the limit. The Source data of all distance of each KArGO cross-links are provided in the Source Data file.

**d)** Theoretically cross-linkable and experimentally observed R-R or K-R pairs in six model proteins.

a

b


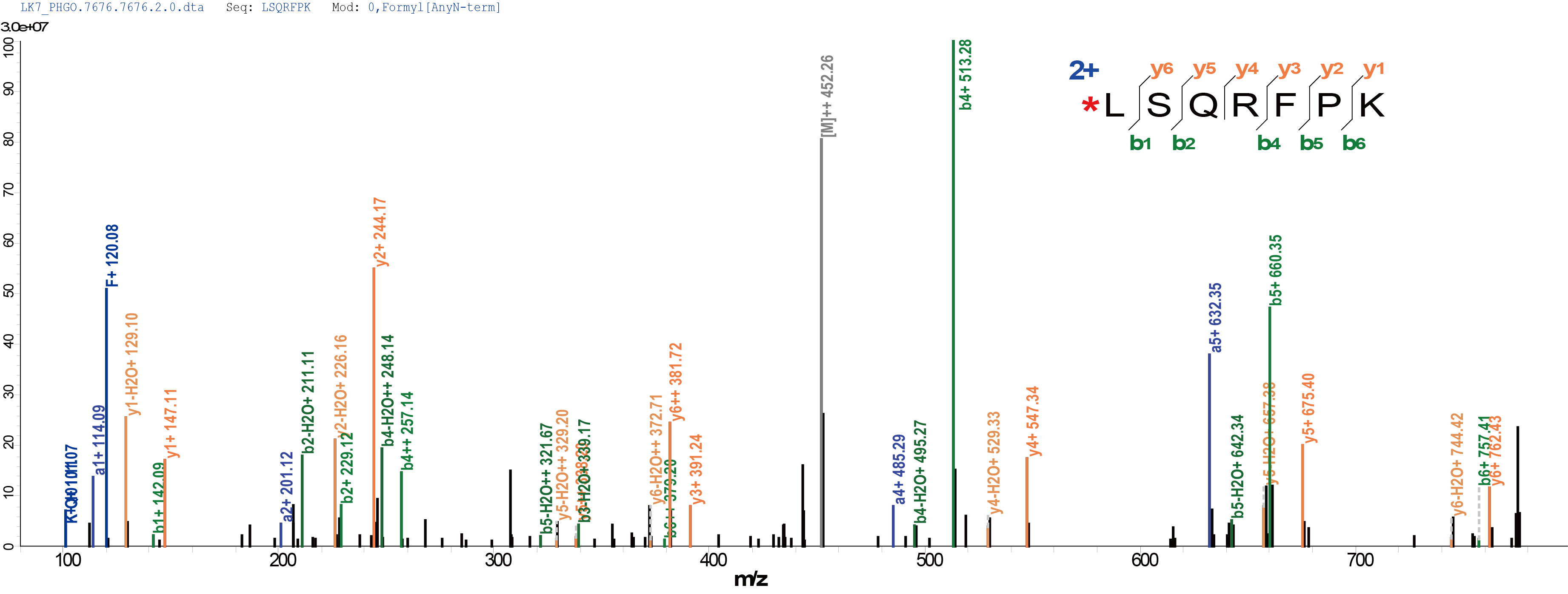


Supplementary Figure 8. Sodium periodate treatment of ArGO-ligation reactions

**a)** Treatment of N-acetyl arginine methyl ester (50 mM) and *p*-OMe phenyl glyoxal (**1**) (25 mM) reaction with NaIO_4_ (10 eq.). Oxidative cleavage of **2** by NaIO_4_ resulted in a mixture of N-(N-formylcarbamimidoyl) benzamide **A1** and N-carbamimidoylbenzamide **A2**. The formyl group of **A1** was quantitatively removed by addition of hydrazine monohydrate (20 eq.), which also quenched unreacted NaIO­4 with release of nitrogen gas.

b) The MS/MS spectrum of N terminal formylated LK7.


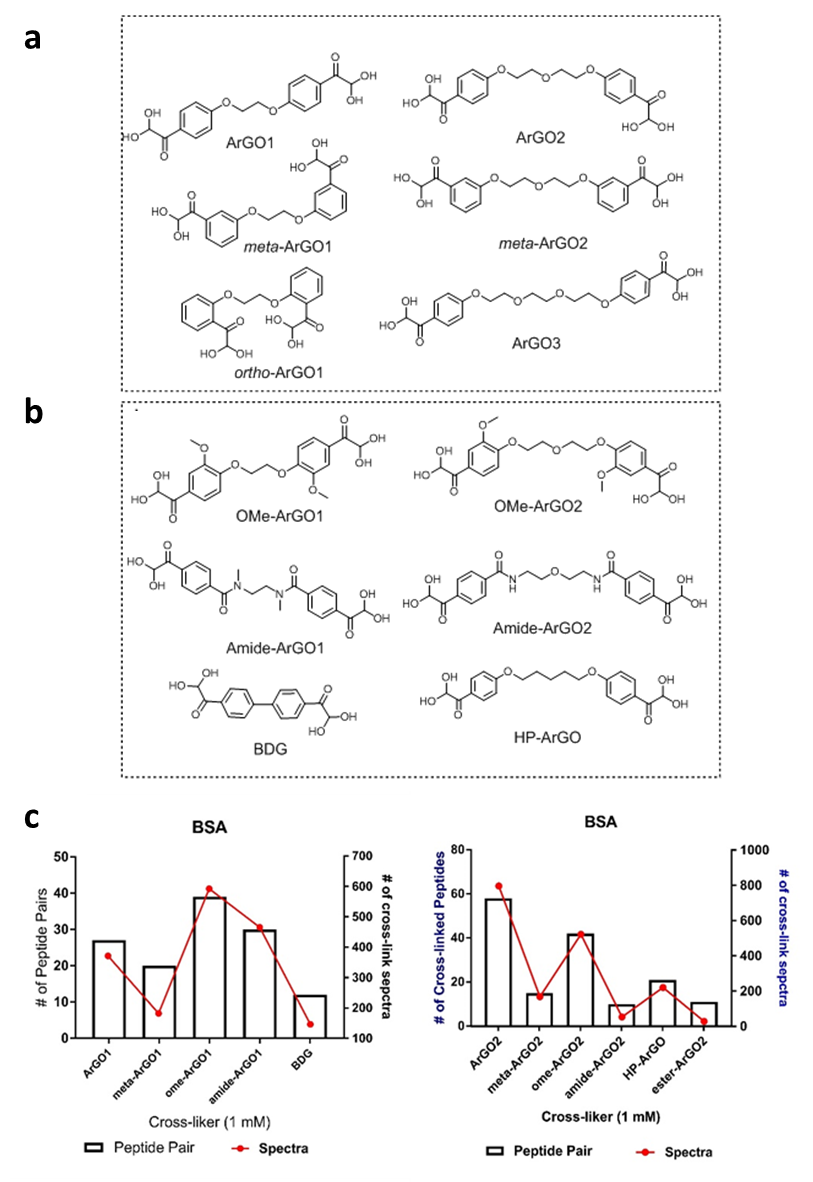


Supplementary Figure 9. Structures of ArGO analogues and number of BSA peptide pairs identified with each

**a)** Ortho- and meta-substituted analogues of ArGO 1-3.

**b)** Electron rich (OMe-ArGO), and electron-deficient (amide-ArGO) variants of ArGO 1-2. Structure of previously reported arginine cross-linker, BDG.^2^ Hydrophobic (HP)-ArGO possesses a C5 alkyl chain spacer.

**c)** Number of non-redundant cross-linked peptides and spectra identified with ArGO analogues from BSA. Reaction conditions employed: 6 mg/mL BSA, 1 mM ArGO in 100 mM borate buffer pH 7.5, 1 h, 25 °C.


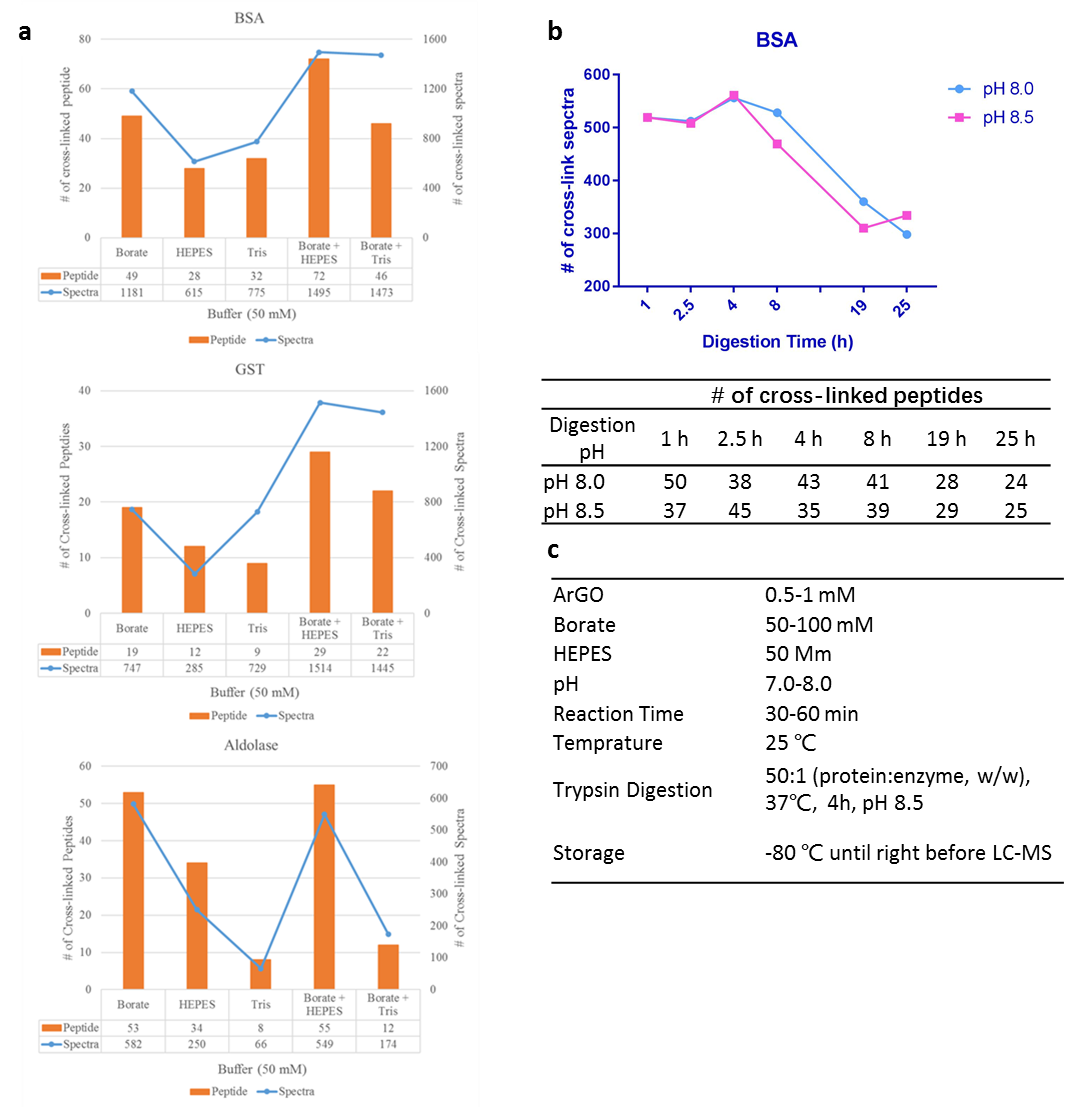


Supplementary Figure 10. Optimisation of cross-linking buffer and trypsin digestion for ArGO2

**a)** ArGO2 cross-linking in borate, HEPES, and Tris buffer, 50 mM each, either alone or in combination (pH 7.5), were tested on BSA, Glutathione-S-transferase (GST) and Aldolase. Number of uniquely identified ArGO-linked peptide pairs (orange bars, those sharing the same linked arginine residues are merged as one) and their total spectral counts (blue line) are shown. The indicated proteins (0.6 mg/ml each) were treated with 1 mM ArGO2 for 1 h at RT.

**b)** Optimization of trypsin digestion time and pH. ArGO-cross-linked BSA was digested with trypsin at 50:1 (protein:trypsin) under pH 8.0 or pH 8.5.

**c)** The optimized cross-linking conditions for ArGO.

**
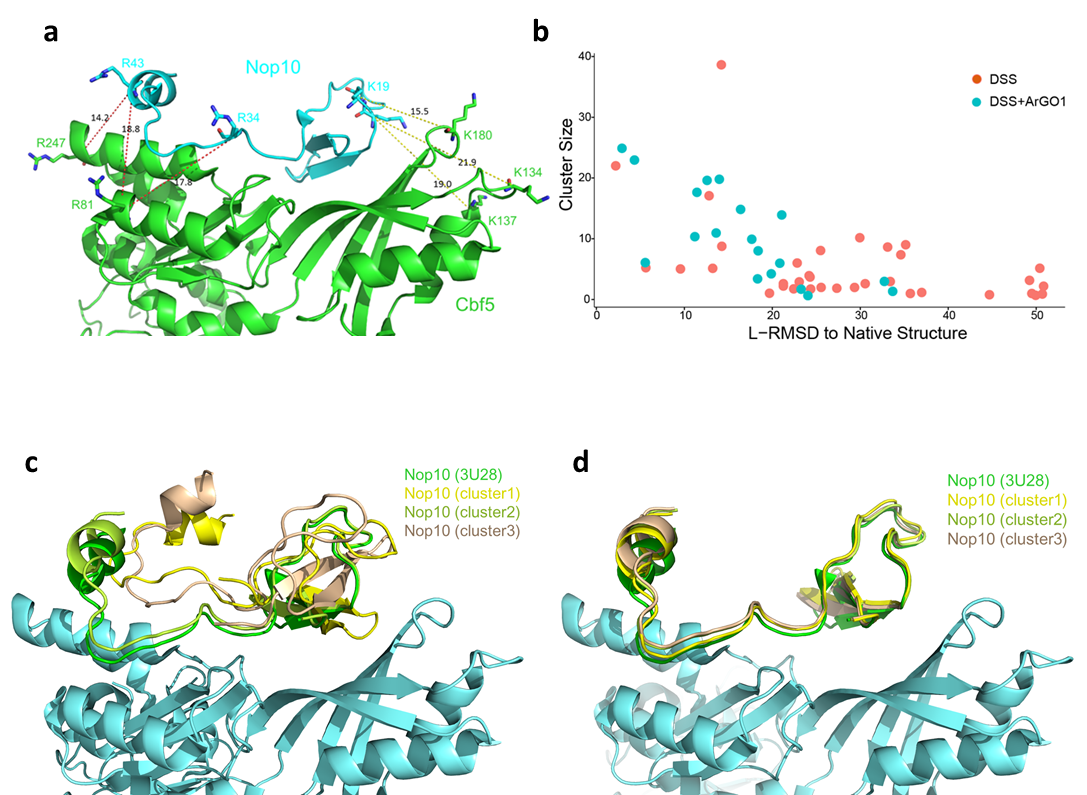
**

Supplementary Figure 11. Structure modelling of CNG complex using Rosetta 3.5 with prepack protocol for pre-treatment.

**a)** Cross-links identified with DSS or ArGO between Nop10 and Cbf5 are mapped to the yeast CNG subcomplex structure (PDB code: 3U28). Between Nop10 and Cbf5, three K-K cross-links were identified with DSS (dotted yellow lines) and three R-R cross-links were identified with ArGO1 (dotted red lines).

**b)** Summary of Rosetta docking results with DSS or DSS + ArGO1 distance restraints. Each dot represents a cluster of conformations thus obtained, with the cluster size (number of poses in a cluster) and ligand−RMSD (the distance between a representative pose of a cluster and the native structure.

**c,d)** After local docking, the three largest clusters (1-3) obtained from Rosetta docking using only DSS restraints (c) or DSS + ArGO restraints (d) are superimposed with the crystal structure (3U28). The interface between Cbf5 (bottom) and Nop10 (top) is shown.

**
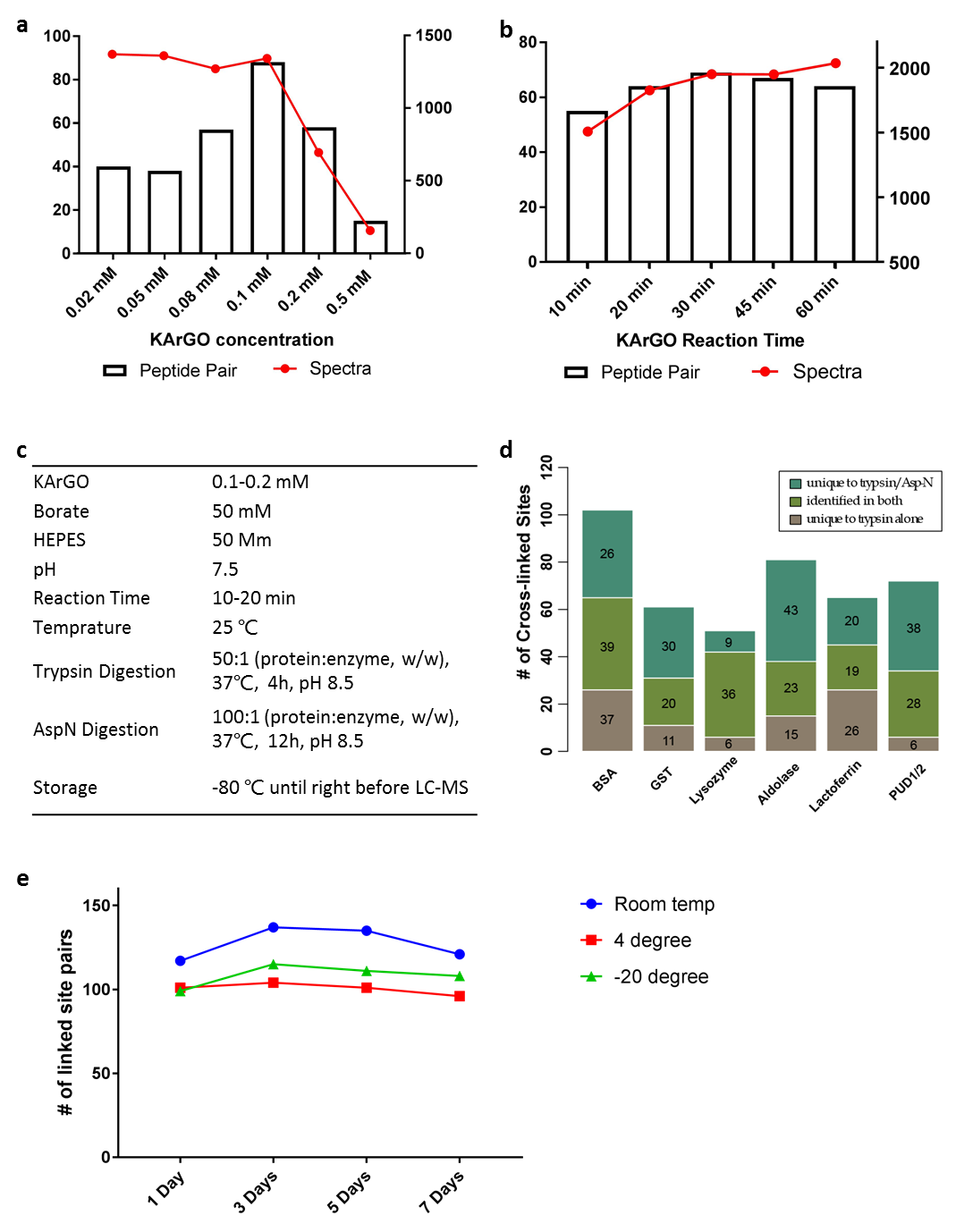
**

Supplementary Figure 12. Optimization of KArGO cross-linking conditions using BSA

**a)** Number of uniquely identified KArGO-linked peptide pairs (black bars, peptide pairs cross-linked through the same pair of arginine residues are merged as one) and their total spectral counts (red line) using KArGO concentration from 0.02 – 0.5 mM.

**b)** Number of KArGO-linked peptide pairs (orange bars) and spectral counts (blue line) identified from cross-linking reactions from 10 – 60 min cross-linking reactions at RT.

**c)** The optimized condition for arginine-lysine cross-linking using KArGO.

**d)** Optimization of digestion protocol. KArGO-cross-linked BSA was incubated with trypsin alone (1:50 protein:trypsin) for 4 h at pH 8.5, or with AspN (ratio 1：100) for 12 h followed by trypsin. Digested peptides were analysed by LC-MS/MS and the data were searched using pLink software to identify cross-linked peptide pairs. Source data of identification results of trypsin and trypsin plus AspN digestion are provided in Source Data file.

**e)** Number of KArGO cross-links identified from a mixture of BSA and aldolase after the cross-linked proteins were precipitated and stored at the indicated temperature for 1 - 7 days.
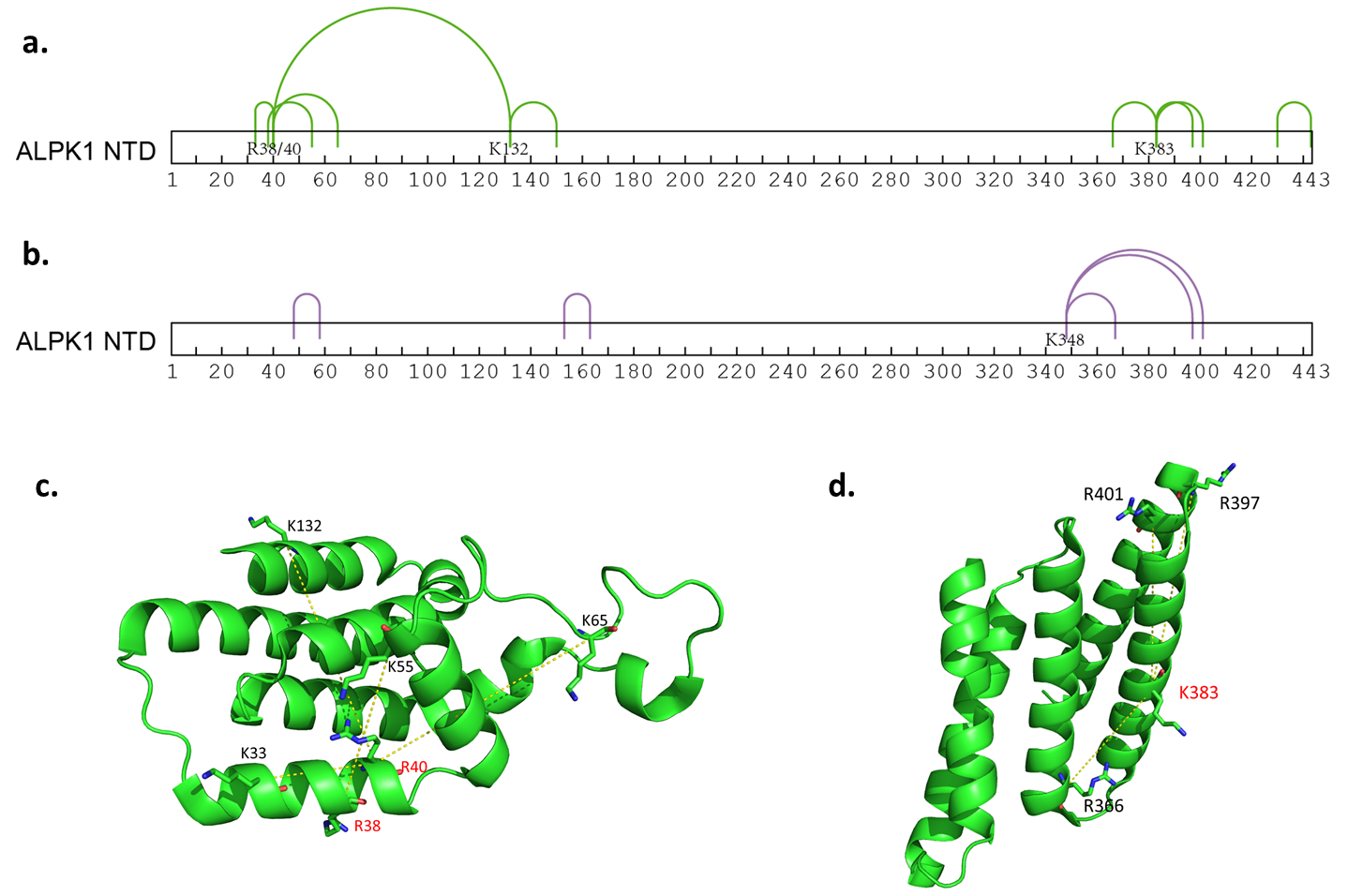


Supplementary Figure 13. KArGO CXMS analysis of ALPK1 N-terminal domain (NTD)

NTD was analysed in the presence of ADP-β-D-manno-heptose, with or without the kinase domain (KD) (959-1244) of ALPK1.

**a-b)** Abundantly identified K-R cross-links within the NTD in the absence **a)** or presence **b)** of the kinase domain. Filter criteria: no. of spectra > 10; when the denominator is zero, spectral fold change >5, and best E-value < 1e-4.

**c-d)** Visualization of the cross-links involving R38 or R40 **c)**, and K383 **d)**, as mapped onto a crystal structure of ALPK1 NTD (PDB: 5Z2C).

# **Supplementary Tables**

Supplementary Table 1. **Mass shifts (ΔM, in Da) introduced by ArGO or KArGO to cross/loop-linked or mono-linked peptides**


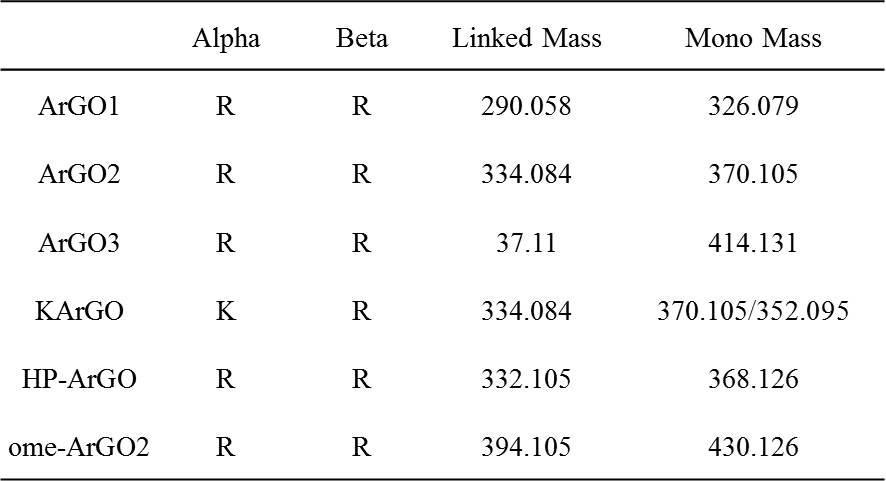


Supplementary Table 2. **Summary of the inter-protein cross-links identified from the CNGP complex after KArGO cross-linking**

| **Linked Site** | **Total Spec** | **Best E-value** | **Distance** |
| --- | --- | --- | --- |
| Nop10 (19)-Nhp2 (118) | 170 | 8.40E-29 | NA |
| Nop10 (34)-Nhp2 (107) | 75 | 1.66E-13 | NA |
| Nop10 (34)-Cbf5 (97) | 68 | 2.87E-12 | 11.37 |
| Nop10 (40)-Cbf5 (81) | 64 | 4.87E-05 | 15.66 |
| Nop10 (19)-Cbf5 (128) | 60 | 8.69E-22 | 17.88 |
| Nop10 (1)-Cbf5 (128) | 54 | 3.60E-23 | NA |
| Nop10 (19)-Cbf5 (181) | 29 | 1.59E-28 | 13.46 |
| Nop10 (34)-Gar1 (77) | 29 | 9.57E-07 | 51.63 |
| Gar1 (77)-Cbf5 (338) | 27 | 3.20E-11 | 54.05 |
| Nop10 (34)-Nhp2 (61) | 27 | 3.98E-07 | NA |
| Gar1 (23)-Cbf5 (114) | 26 | 1.89E-18 | 37.59 |
| Nhp2 (107)-Cbf5 (181) | 18 | 8.09E-24 | NA |
| Cbf5 (31)-Nop10 (34) | 17 | 4.40E-07 | 45.44 |
| Gar1 (90)-Cbf5 (161) | 15 | 2.34E-14 | 17.15 |
| Gar1 (84)-Cbf5 (338) | 14 | 2.87E-08 | 44.04 |
| Gar1 (84)-Cbf5 (165) | 13 | 1.76E-05 | 17.25 |
| Cbf5 (50)-Nop10 (51) | 13 | 1.27E-05 | NA |
| Gar1 (41)-Cbf5 (165) | 12 | 1.08E-04 | 29.55 |
| Gar1 (28)-Cbf5 (349) | 10 | 5.80E-17 | 61.11 |
| Gar1 (46)-Cbf5 (349) | 10 | 1.11E-08 | 69.49 |
| Nop10 (34)-Nhp2 (69) | 10 | 5.78E-04 | NA |
| Gar1 (28)-Cbf5 (165) | 9 | 1.31E-04 | 14.21 |
| Gar1 (77)-Cbf5 (349) | 8 | 3.81E-12 | 75.34 |
| Cbf5 (50)-Gar1 (90) | 7 | 2.11E-04 | 51.12 |
| Nop10 (51)-Cbf5 (337) | 6 | 2.93E-12 | NA |
| Nop10 (34)-Cbf5 (337) | 5 | 3.05E-12 | 39.47 |
| Gar1 (84)-Cbf5 (348) | 5 | 2.25E-06 | 63 |
| Nop10 (51)-Gar1 (84) | 5 | 2.97E-10 | NA |
| Gar1 (77)-Nhp2 (118) | 5 | 5.36E-07 | NA |
| Nop10 (34)-Gar1 (84) | 5 | 1.96E-06 | 48.98 |
| Gar1 (23)-Cbf5 (87) | 5 | 1.11E-12 | 49.21 |
| Nhp2 (118)-Cbf5 (180) | 5 | 1.51E-06 | NA |
| Gar1 (84)-Cbf5 (128) | 4 | 5.81E-15 | 33.66 |
| Gar1 (77)-Cbf5 (141) | 4 | 1.01E-08 | 24.15 |
| Nop10 (12)-Cbf5 (349) | 3 | 1.55E-17 | 48.56 |
| Nop10 (18)-Cbf5 (128) | 3 | 6.16E-13 | 15.52 |
| Gar1 (23)-Cbf5 (337) | 3 | 1.59E-15 | 44.85 |

Filter criteria: # of spectra > 3, and best E-value < 1e-3. The CNG complex structure (pdb code, 3u28) is used as reference. Cbf5 refers to chain A, Nop10 refers to chain B, and Gar1 refers to chain C.

Supplementary Table 3. **KArGO cross-links within the ALPK1 NTD which were affected by the presence or absence of the C-terminal kinase domain (KD)**

|  | **Linked Site** | **NTD+KD** | | **NTD alone** | | **Distance ED (Å)** |
| --- | --- | --- | --- | --- | --- | --- |
|  |  | **# Spectrum** | **Best E-value** | **# Spectrum** | **Best E-value** |  |
| 1 | ALPK1(40)-ALPK1(33) |  |  | 39 | 1.10E-10 | 10.55 |
| 2 | ALPK1(40)-ALPK1(65) | 3 | 7.92E-12 | 18 | 1.05E-07 | 22.27 |
| 3 | ALPK1(40)-ALPK1(329) |  |  | 16 | 5.64E-08 | 22.56 |
| 4 | ALPK1(38)-ALPK1(55) |  |  | 10 | 1.88E-05 | 17.51 |
| 5 | ALPK1(40)-ALPK1(132) |  |  | 10 | 5.55E-05 | 22.04 |
| 6 | ALPK1(132)-ALPK1(150) | 8 | 9.63E-11 | 38 | 3.25E-16 | 12.74 |
| 7 | ALPK1(383)-ALPK1(401) |  |  | 35 | 1.72E-10 | 17.83 |
| 8 | ALPK1(383)-ALPK1(397) |  |  | 32 | 1.20E-06 | 21.15 |
| 9 | ALPK1(383)-ALPK1(366) |  |  | 18 | 2.31E-05 | 15.07 |
| 10 | ALPK1(430)-ALPK1(443) | 10 | 1.87E-24 | 69 | 1.71E-10 | 15.08 |
|  |  |  |  |  |  |  |
| 11 | ALPK1(48)-ALPK1(58) | 13 | 6.89E-08 |  |  | 13.76 |
| 12 | ALPK1(153)-ALPK1(163) | 11 | 2.06E-08 |  |  | 10.15 |
| 13 | ALPK1(348)-ALPK1(401) | 103 | 6.11E-21 | 4 | 3.23E-06 | 9.42 |
| 14 | ALPK1(348)-ALPK1(397) | 56 | 1.96E-15 |  |  | 13.56 |
| 15 | ALPK1(348)-ALPK1(367) | 12 | 5.69E-07 |  |  | 28.11 |

Filter criteria: # of spectrum > 10; when the denominator is zero, spectral fold change >5, and best E-value < 1e-4. The ALPK1 NTD structure (pdb code, 5z2c) is used as reference. Note: All cross-links except the R40-K329 are shown in fig. S15 a-b, because R40-K329 is most likely an inter-molecular cross-link. The minimal Cα-Cα distance R40 and K329 is 22.56 Å. The total spectra count of the linear peptides of ALPK1 NTD number of MS2 spectra of ALPK1 NTD peptides in the presence or absence of KD is 11005 or 10279, respectively.

Supplementary Table 4. **UtpA Cross-linking results with BS^3^ and KArGO**

The reaction conditions are described in the methods section. Filter criteria: # of spectra > 5, and best E-value < 1e-3. The UtpA sub-complex structure (pdb code, 5wlc) is used as reference. Utp4 refers to chain LN, Utp5 refers to chain LL, Utp8 refers to chain LI, Utp9 refers to chain LK, Utp15 refers to chain LJ.

**a)** Overview of UtpA cross-linking with BS^3^ and KArGO.

**b)** Spectral count, E-value and structural compatibility of 22 inter-molecular BS^3^ cross-links.

**c)** Spectral count, E-value and structural compatibility of 14 inter- molecular KArGO cross-links.

**a. Summary of UtpA Cross-linking results with BS3 and KArGO**

| **Cross-Linker** | **Total  # of X-links** | **Intra-subunit  X-links** | **Inter-subunit  X-links** | **Structural Compatibility (intra-subunit)** | **Structural Compatibility  (inter-subunit)** |
| --- | --- | --- | --- | --- | --- |
| BS^3^ | 126 | 104 | 22 | 66% (<24 Å) | 11% (<30 Å) |
|  |  |  |  | 80% (<30 Å) |  |
| KArGO | 57 | 43 | 14 | 97% (<32.2 Å) | 57% (<32.2 Å) |

**b. The inter-molecular site pairs identified from UtpA complex cross-linked with BS^3^**

| **Linked Sites** | **Total Spectra** | **Best E-value** | **Type** | **Distance (Å)** | **< 30 Å?** |
| --- | --- | --- | --- | --- | --- |
| Utp4(767)-Utp8 (483) | 39 | 3.26E-21 | Inter | NA |  |
| Utp4(371)-Utp8 (483) | 35 | 2.24E-31 | Inter | NA |  |
| Utp4(765)-Utp8 (483) | 27 | 1.29E-31 | inter | NA |  |
| Utp15 (497)-Utp4(773) | 19 | 2.69E-15 | inter | 43.5 | No |
| Utp5 (553)-Utp8 (483) | 16 | 1.33E-26 | inter | NA |  |
| Utp4(354)-Utp8 (483) | 14 | 6.61E-12 | inter | NA |  |
| Utp4(718)-Utp5 (473) | 14 | 6.56E-17 | inter | NA |  |
| Utp4(313)-Utp5 (473) | 14 | 2.39E-20 | inter | 98.6 | No |
| Utp4(316)-Utp5 (473) | 13 | 5.27E-10 | inter | 102.3 | No |
| Utp15 (396)-Utp4(624) | 13 | 7.27E-14 | inter | 49.6 | No |
| Utp8 (483)-Utp9 (473) | 11 | 1.69E-17 | inter | NA |  |
| Utp4(767)-Utp5 (553) | 11 | 2.48E-20 | inter | 35.1 | No |
| Utp8 (483)-Utp9 (418) | 10 | 3.53E-18 | inter | NA |  |
| Utp4(21)-Utp8 (483) | 10 | 2.42E-14 | inter | NA |  |
| Utp15 (396)-Utp4(718) | 9 | 9.89E-12 | inter | NA |  |
| Utp4(24)-Utp5 (473) | 7 | 4.17E-11 | inter | NA |  |
| Utp4(17)-Utp5 (473) | 7 | 1.01E-05 | inter | NA |  |
| Utp5 (473)-Utp8 (483) | 6 | 5.31E-18 | inter | NA |  |
| Utp4(371)-Utp5 (473) | 6 | 3.18E-20 | inter | 88.3 | No |
| Utp15 (396)-Utp4(59) | 6 | 1.03E-12 | inter | 52.3 | No |
| Utp15 (396)-Utp4(371) | 6 | 9.92E-18 | inter | 31.3 | No |
| Utp15 (488)-Utp9 (417) | 6 | 2.13E-12 | inter | 20.1 | Yes |

**c. The inter-molecular site pairs identified from UtpA complex cross-linked with KArGO**

| **Linked Sites** | **Total Spectra** | **Best E-value** | **Type** | **Distance(Å)** | **< 32.2 Å?** |
| --- | --- | --- | --- | --- | --- |
| Utp5 (463)-Utp9 (511) | 340 | 1.67E-11 | inter | 12.4 | Yes |
| Utp5 (471)-Utp9 (511) | 130 | 6.39E-07 | inter | 16.6 | Yes |
| Utp5 (466)-Utp9 (511) | 71 | 2.72E-12 | inter | 11 | Yes |
| Utp15 (448)-Utp5 (490) | 32 | 1.41E-12 | inter | 15.7 | Yes |
| Utp15 (396)-Utp4(189) | 23 | 9.22E-13 | inter | 38 | No |
| Utp15 (497)-Utp5 (463) | 21 | 2.91E-08 | inter | 22.3 | Yes |
| Utp15 (396)-Utp5 (487) | 13 | 3.42E-08 | inter | 40.1 | No |
| Utp15 (492)-Utp9 (417) | 13 | 1.32E-14 | inter | 21.5 | Yes |
| Utp4(313)-Utp5 (490) | 12 | 1.06E-15 | inter | 73.5 | No |
| Utp8 (484)-Utp9 (417) | 10 | 6.69E-09 | inter | NA |  |
| Utp4(316)-Utp5 (490) | 8 | 1.16E-10 | inter | 76.9 | No |
| Utp8 (332)-Utp9 (417) | 8 | 1.89E-17 | inter | 50.7 | No |
| Utp15 (428)-Utp5 (553) | 6 | 3.48E-15 | inter | 23 | Yes |
| Utp15 (396)-Utp8 (332) | 6 | 2.61E-07 | inter | 70.1 | No |

# Supplementary Note

**Supplementary Note 1. Chemoselectivity evaluation of ArGO with synthetic peptides**

5 mM of *p*-OMe-PhGO (**1**) was added to 1 mM peptide in 50 mM HEPES and 50 mM borate buffer pH 7.5. The reaction products were analysed by LC-MS/MS.

Supplementary Table 5. Peptides tested for ArGO selectivity

| Peptide | Sequence | [M+H]^+^ | Containing R |
| --- | --- | --- | --- |
| HK-7 | HPVCAYK | 817.403 | No |
| DK-10 | DGMIKLWDLK | 1218.655 | No |
| VR-6 | VKTELR | 745.457 | Yes |
| LK-7 | LSQRFPK | 875.510 | Yes |
| FR-9 | FVKQQWNLR | 1218.674 | Yes |
| SR-14 | SDFKFSNLLGTVYR | 1646.854 | Yes |
| Ac-IR-7 | Ac-IEAEKGR | 844.452 | Yes |

Peptides are modified after reacting with *p*-OMe-PhGO. For each product, the mass shift from the intact peptide is indicated, and the relative abundance against the major product (+146.04) is calculated from the corresponding chromatographic peak areas.

Supplementary Table 6. Distribution of peptide products after *p*-OMe-PhGO treatment

* incubated with 5 mM TCEP at 56 ⁰C for 10 min after *p*-OMe-PhGO treatment.

| **Mass shift from intact peptide** | **Arginine-free peptide** | | **Arginine-containing peptide** | | | | | **Nature of modified species** | **Mod. site as localized by MS2** | **Note** |
| --- | --- | --- | --- | --- | --- | --- | --- | --- | --- | --- |
|  | HK-7 | DK-10 | VR-6  /VR-6* | LK-7  /LK-7* | FR-9 | SR-14 | Ac-IR-7 |  |  |  |
| **+146.04 Da** | - | - | 1  /1 | 1  /0.97 | 1 | 1 | 1 | Product **3** in Supplementary Figure 4 | Arginine |  |
| **+162.03 Da** | - | - | 0.08  /0.12 | 0.08  /0.06 | 0.08 | 0.04 | 0.11 | Oxidation product **2** in Supplementary Figure 4 | Arginine |  |
| **+310.09 Da** | - | - | 0.08  /0.02 | 0.18  /0.06 | - | - | - | Product **6** in Supplementary Figure 4 | Arginine |  |
| **+164.05 Da** | 0.05 | - | 0.26 /0.02 | 0.37 /0.01 | 0.47 | 0.12 | 0.74 | Non-covalent conjugate; *p*-OMe-PhGO readily dissociates as a neutral. | Not on any particular residue; MS2 the same as that of the intact peptide | Greatly reduced after TCEP treatment |
| **+134.04 Da** | - | - | 1.38 /0.74 | 0.37 /0.27 | - | 0.01 | - | Not determined; a strong 1+ peak of 135.04 m/z in MS2 | N- terminus | Elute after +134.04 on lysine; reduced after TCEP treatment |
| **+134.04 Da** | 1 | - | - | 0.04  /0.03 | 0.01 | - | 0.15 | Not determined; a strong 1+ peak of 135.04 m/z in MS2 | Lysine | Elute before +134.04 on the N-terminus |
| **+27.99 Da** | - | - | 0.45  /0.36 | 0.17  /0.20 | 0.18 | - | - | Formylation | N- terminus |  |

The chromatographic peaks and the MS2 spectra of intact or modified peptides were analysed manually, assisted by several software tools for spectrum labeling, de novo sequencing, expected or unexpected PTM search.

**
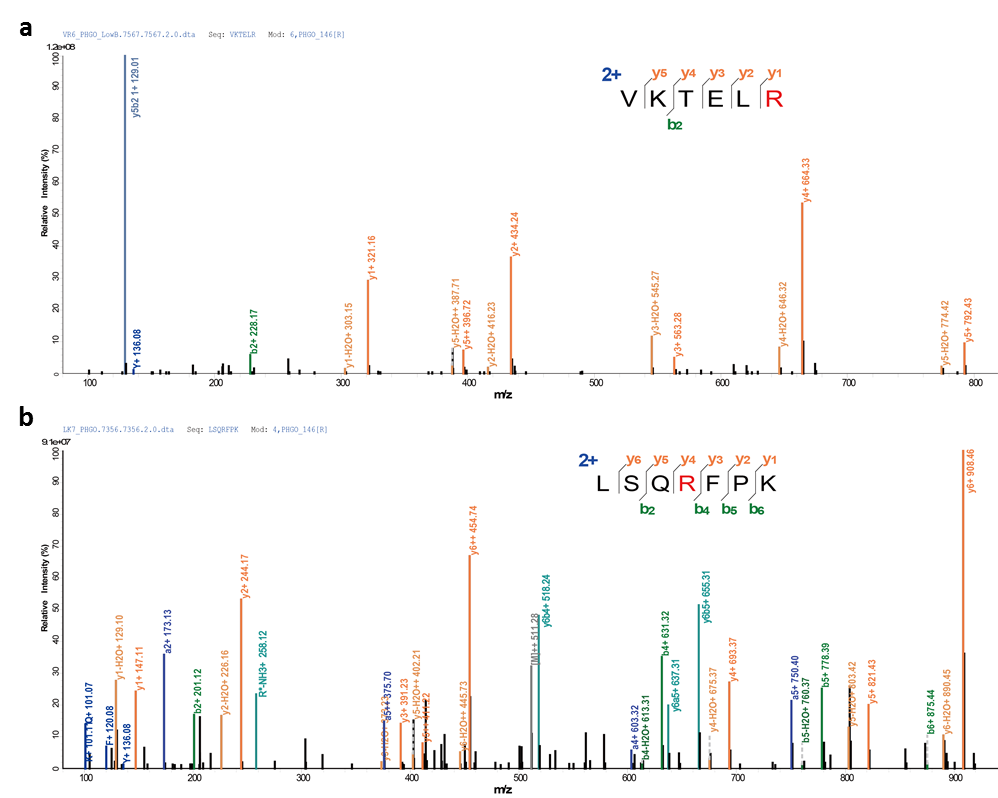
**

**
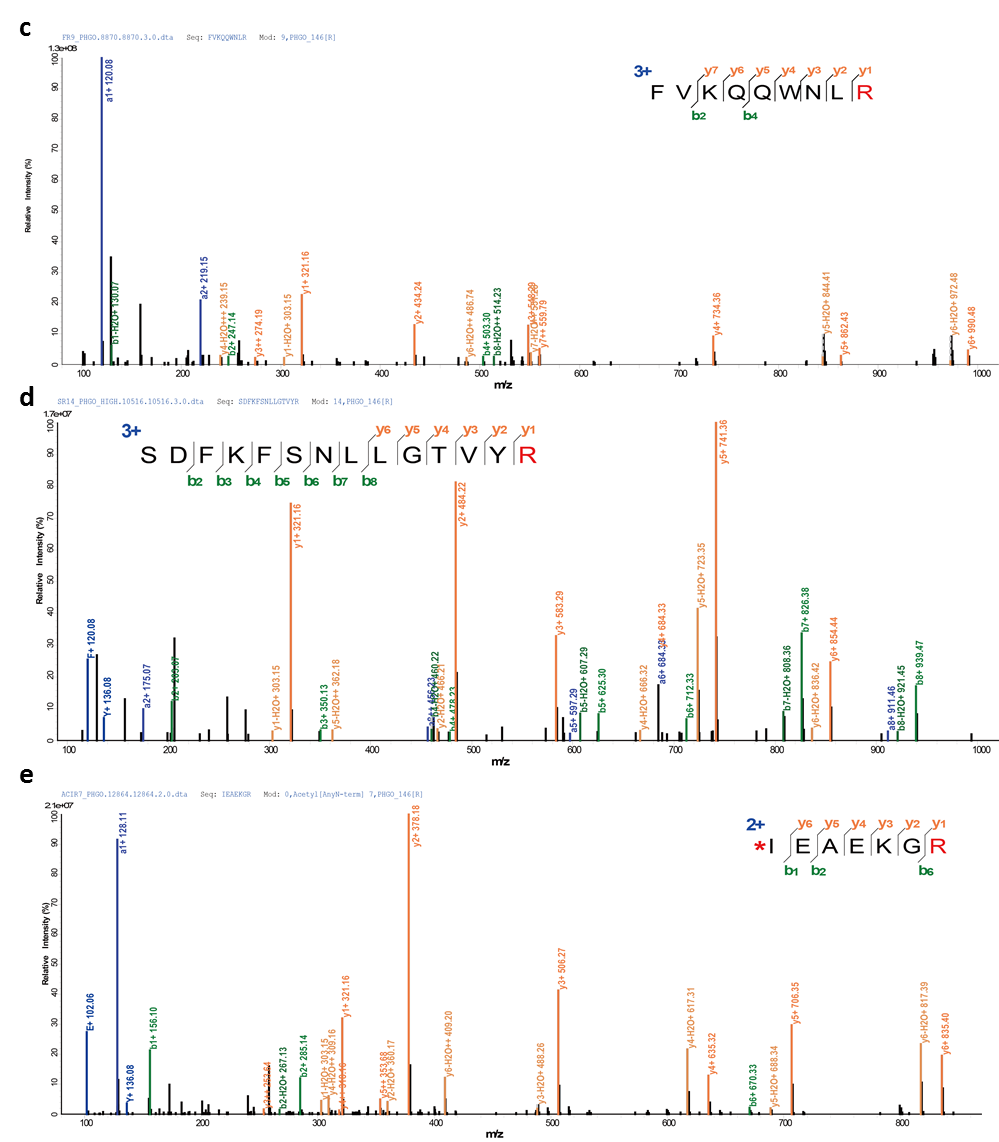
**

Supplementary Figure 14. MS2 of major product of each of the seven peptides (product 3 in Supplementary Figure 4)

**a)** MS/MS spectrum of VR-6 + 146.04 Da

**b)** MS/MS spectrum of LK-7 + 146.04 Da. (R*-NH3+ is the 1+ deaminated imonium ion of arginine to which *p*-OMe-PhGO is attached covalently.)

**c)** MS/MS spectrum of FR-9 + 146.04 Da

**d)** MS/MS spectrum of SR-14 + 146.04 Da

**e)** MS/MS spectrum of Ac-IR-7 + 146.04 Da

**Supplementary Note 2. Relax flag file (Rosetta 3.10 only)**

-nstruct 1

-in:file:s combine.pdb

-relax:constrain_relax_to_start_coords

-relax:ramp_constraints false

-ex1

-ex2

-flip_HNQ

-no_optH false

-overwrite

**Supplementary Note 3. Global docking flag file**

-s ../3u28_relaxed.pdb # input pdb name

-partners AB_C # docking partner

-dock_pert 3 8

-low_res_protocol_only

-randomize1

-randomize2

-spin

-constraints:cst_file ../LowRes_cng_KArGO

-no_filters

-run:debug

-run:use_time_as_seed true

-run:seed_offset 1 #add to random seed

-out:nstruct 500

-out:overwrite

-out:prefix sd1193_

-out:file:scorefile sd1193_LowRes_CNGP.fasc

-out:path:pdb ./

-out:path:score ./

**Supplementary Note 4.** **cts file**

cts file for docking with DSS restraint:

AtomPair CA 137A CA 18C BOUNDED 0 24 1 0.5 CNGP

AtomPair CA 134A CA 18C BOUNDED 0 24 1 0.5 CNGP

AtomPair CA 180A CA 19C BOUNDED 0 24 1 0.5 CNGP

cts file for docking with DSS + ArGO restraint:

AtomPair CA 81A CA 34C BOUNDED 0 29.4 1 0.5 CNGP

AtomPair CA 81A CA 43C BOUNDED 0 29.4 1 0.5 CNGP

AtomPair CA 247A CA 43C BOUNDED 0 29.4 1 0.5 CNGP

AtomPair CA 137A CA 18C BOUNDED 0 24 1 0.5 CNGP

AtomPair CA 134A CA 18C BOUNDED 0 24 1 0.5 CNGP

AtomPair CA 180A CA 19C BOUNDED 0 24 1 0.5 CNGP

cts file for docking with KArGO restraint:

AtomPair CA 81A CA 40C BOUNDED 0 32.2 1 0.5 CNGP

AtomPair CA 97A CA 34C BOUNDED 0 32.2 1 0.5 CNGP

AtomPair CA 128A CA 18C BOUNDED 0 32.2 1 0.5 CNGP

AtomPair CA 128A CA 19C BOUNDED 0 32.2 1 0.5 CNGP

AtomPair CA 181A CA 19C BOUNDED 0 32.2 1 0.5 CNGP

**Supplementary Note 5. Synthesis of ArGO1-3**

Supplementary Figure 15. **Synthesis Procedures of ArGO 1-3**

- 1. **General Procedure for Synthesis of pre-ArGO 1-3**

A mixture of the *p*-hydroxyacetophenone (408 mg, 3 mmol), dihalide linker (1 mmol) and Cs_2_CO_3_ (1.63 g, 5mmol) in MeCN (8 mL) were heated for 6 hours in a sealed flask at 80 ^°^C. After cooling to room temperature, the solvent was evaporated *in vacuo*. The concentrated material was dissolved in EtOAc and extracted sequentially with 1 M NaOH and brine. The organic layer was dried over Na_2_SO_4_, filtered, and the solvent was evaporated. The crude product was purified by flash column chromatography (gradient of 80:20 to 50:50 petroleum ether:EtOAc).

**pre-ArGO1**

Supplementary Figure 16. **Spectroscopic Data for pre-ArGO1**

White solid; 226 mg, 76% yield from 1 mmol dibromoethane and 3 mmol 4-hydroxyacetophenone: m.p. 167-169 $℃$; IR (neat) 1666, 1602, 1253 cm^-1^; ^1^H NMR (400 MHz, CDCl_3_)$\delta$ 7.95 (d, *J* = 8.8 Hz, 4H), 6.99 (d, *J* = 8.8 Hz, 4H), 4.41 (s, 4H), 2.56 (s, 6H); ^13^C NMR (100 MHz CDCl_3_)$\delta$26.5, 66.6, 114.4, 130.8, 130.9, 162.5, 196.9; MS (ESI) *m/z* 299 (M + H)^+^; HRMS (ESI) *m/z* calcd for C_18_H_19_O_4_ (M + H)^+^ 299.12779, found 299.12764.

**pre-ArGO2**

Supplementary Figure 17. **Spectroscopic Data for pre-ArGO2**

White solid; 278 mg, 81% yield from 1 mmol 2,2’-diiodoethyl ether and 3 mmol 4-hydroxyacetophenone: m.p. 113-115 $℃$; IR (neat) 1676, 1601, 1257, 1065 cm^-1^; ^1^H NMR (400 MHz, CDCl_3_) $\delta$ 7.91 (d, *J* = 8.8 Hz, 4H), 6.93 (d, *J* = 8.8 Hz, 4H), 4.21 (t, *J* = 4.6 Hz, 4H), 3.95 (t, *J* = 4.7 Hz, 4H), 2.54 (s, 6H); ^13^C NMR (100 MHz CDCl_3_)$\delta$26.5, 67.7, 69.9, 114.4, 130.6, 130.7, 162.7, 196.9; MS (ESI) *m/z* 343 (M + H)^+^; HRMS (ESI) *m/z* calcd for C_20_H_23_O_5_ (M + H)^+^ 343.15400, found 343.15400.

**pre-ArGO3**

Supplementary Figure 18. **Spectroscopic Data for pre-ArGO3**

White solid; 316 mg, 82% yield from 1 mmol 1,2-bis-(2-iodoethoxy)ethane and 3 mmol 4-hydroxyacetophenone: m.p. 173-175 $℃$; IR (neat) 1673, 1600, 1255 cm^-1^; ^1^H NMR (400 MHz, CDCl_3_) $\delta$ 7.91 (d, *J* = 8.8 Hz, 4H), 6.93 (d, *J* = 8.8 Hz, 4H), 4.18 (t, *J* = 4.7 Hz, 4H), 3.89 (t, *J* = 4.7 Hz, 4H), 3.77 (s, 4H), 2.54 (s, 6H); ^13^C NMR (100 MHz CDCl_3_)$\delta$26.5, 67.7, 69.7, 71.1, 114.4, 130.6, 130.7, 162.8, 196.9; MS (ESI) *m/z* 387 (M + H)^+^, 409 (M + Na)^+^; HRMS (ESI) *m/z* calcd for C_20_H_23_O_6_ (M + H)^+^ 387.18022, found 387.18094.

- 1. **General Procedure for Synthesis of ArGO1-3**

According to a modified literature procedure,^3^ SeO_2_ (60 mg, 0.54 mmol, 5.4 eq.) was added to a solution of pre-ArGO (0.1 mmol) in 450 μL:50 μL 1,4-dioxane:H_2_O. The clear mixture was heated using microwave irradiation to a set temperature of 100 ^°^C for 0.5-1 h, using a max. power of 150 W. After reaction completion (determined by HPLC-MS), the dark-green mixture was cooled to room temperature, filtered to remove Se and excess SeO_2_, and washed with 1,4-dioxane (0.5-1 mL). H_2_O (2 mL) was then added, the mixture was refluxed at 100 ^°^C for 0.25 h, filtered hot to remove further insoluble precipitates, and washed with hot water. MeCN (~0.5 mL) was added to ensure homogeneity of the filtrate, and the mixture was purified by reverse-phase C18 column chromatography using the CombiFlash automatic chromatography system (Gradient: 100:0 H_2_O:MeCN → 80:20 H_2_O:MeCN over 20 minutes). The pure product could be obtained after removal of H_2_O by freeze-drying.

The ArGO compounds exist as an equilibrium between hydrate and aldehyde forms. Resonances for both forms are given separately in the ^1^H NMR data. The characteristic ^13^C splitting pattern for the hydrate-aldehyde equilibrium is identified by three peaks between 180-200 ppm, corresponding to the ketone group for hydrate and aldehyde forms; and an aldehyde peak. The aldehyde peak is given in the ^13^C NMR where possible.

**ArGO1**

Supplementary Figure 19. **Spectroscopic Data for ArGO1**

White solid; 27 mg, 75% yield from 0.1 mmol pre-ArGO-1 (**1**): m.p. 135-137 $℃$; IR (neat) 3400-3100 (br), 1653, 1633 cm^-1^; Ratio of hydrate: aldehyde = 0.93: 0.07; ^1^H NMR for hydrate (400 MHz, (CD_3_)_2_SO) $\delta$ 8.06 (d, *J* = 8.9 Hz, 4H), 7.11 (d, *J* = 8.9 Hz, 4H), 6.66 (d, *J* = 7.2 Hz, 4H), 5.65 (t, *J* = 6.7 Hz, 2H), 4.46 (s, 4H); ^1^H NMR for aldehyde (400 MHz, (CD_3_)_2_SO) $\delta$ 9.51 (s, 2H), 7.90 (d, *J* = 8.9 Hz, 4H), 7.19 (d, *J* = 9.0 Hz, 4H), 4.46 (s, 4H); ^13^C NMR (100 MHz (CD_3_)_2_SO)$\delta$ 66.6, 89.1, 114.3, 126.7, 132.1, 162.3, 194.8; MS (ESI) *m/z* 385 (M + Na)^+^; HRMS (ESI) *m/z* calcd for C_18_H_18_NaO_8_ (M + Na)^+^ 385.08939, found 385.08882.

**ArGO2**

Supplementary Figure 20. **Spectroscopic Data for ArGO2**

White solid; 24 mg, 59% yield from 0.1 mmol pre-ArGO-2 (**2**): m.p. 147-148 $℃$; IR (neat) 3450-3100 (br), 1669, 1597 cm^-1^; Ratio of hydrate: aldehyde =0.84: 0.16; ^1^H NMR for hydrate (400 MHz, (CD_3_)_2_SO) $\delta$ 8.04 (d, *J* = 8.9 Hz, 4H), 7.06 (d, *J* = 8.9 Hz, 4H), 6.66 (d, *J* = 7.3 Hz, 4H), 5.64 (t, *J* = 7.0 Hz, 2H), 4.23 (t, *J* = 4.4 Hz, 4H), 3.85 (t, *J* = 4.4 Hz, 4H); ^1^H NMR for aldehyde (400 MHz, (CD_3_)_2_SO) $\delta$ 9.52 (s, 2H), 8.10 (d, *J* = 8.8 Hz, 4H), 7.13 (d, *J* = 9.0 Hz, 4H), 4.26 (t, *J* = 4.4 Hz, 4H), 3.85 (t, *J* = 4.4 Hz, 4H) [peaks at 4.26, 3.85 ppm overlapped with hydrate peaks]; ^13^C NMR (100 MHz (CD_3_)_2_SO)$\delta$ 67.5, 68.9, 89.0, 114.2, 126.4, 131.8, 162.4, 194.7; MS (ESI) *m/z* 407 (M + H)^+^ , 429 (M + Na)^+^; HRMS (ESI) *m/z* calcd for C_20_H_22_NaO_9_ (M + Na)^+^ 429.11560, found 429.11642.

**ArGO3**

Supplementary Figure 21. **Spectroscopic Data for ArGO3**

White solid; 30 mg, 67% yield from 0.1 mmol pre-ArGO-3 (**3**): m.p. 86-88 $℃$; IR (neat) 3500-3100 (br), 1678, 1599 cm^-1^; Ratio of hydrate: aldehyde =0.87: 0.13; ^1^H NMR for hydrate (400 MHz, (CD_3_)_2_SO) $\delta$ 8.03 (d, *J* = 8.9 Hz, 4H), 7.04 (d, *J* = 8.9 Hz, 4H), 6.65 (d, *J* = 7.4 Hz, 4H), 5.63 (t, *J* = 7.3 Hz, 2H), 4.18 (t, *J* = 4.5 Hz, 4H), 3.76 (t, *J* = 4.5 Hz, 4H), 3.61 (t, *J* = 4.5 Hz, 4H); ^1^H NMR for aldehyde (400 MHz, (CD_3_)_2_SO) $\delta$ 9.51 (s, 2H), 8.05 (d, *J* = 9.0 Hz, 4H), 7.11 (d, *J* = 9.0 Hz, 4H), 4.18 (t, *J* = 4.5 Hz, 4H), 3.76 (t, *J* = 4.5 Hz, 4H), 3.61 (t, *J* = 4.5 Hz, 4H) [peaks at 4.22, 3.77 ppm overlapped with hydrate peaks]; ^13^C NMR (100 MHz (CD_3_)_2_SO)$\delta$67.5, 68.8, 70.0, 89.0, 114.2, 126.4, 131.8, 162.5, 194.7; MS (ESI) *m/z* 473 (M + Na)^+­^; HRMS (ESI) *m/z* calcd for C_22_H_26_NaO_10_ (M + Na)^+^473.14182, found 473.14110.

**Supplementary Note 6. Synthetic Procedures and Spectroscopic Data Towards KArGO**

Supplementary Figure 22. Synthetic Procedures of KArGO

A mixture of *p-*hydroxyacetophenone (65 mg, 0.48 mmol), alkyl iodide **S1** (100 mg, 0.24 mmol) and Cs_2_CO_3_ (235 mg, 0.72 mmol) in dry MeCN (5 mL) were heated for six hours in a sealed flask at 80 ^°^C. After cooling to room temperature, the solvent was evaporated *in vacuo*. The concentrated material was dissolved in EtOAc and extracted with 1 M NaOH and brine. The organic layer was dried over Na_2_SO_4_, filtered, and the solvent was evaporated. The crude product was purified by flash column chromatography (gradient of 80:20 to 50:50 petroleum ether:EtOAc) to afford **S2** as a clear oil ((81 mg, 81**%** yield).

Supplementary Figure 23. **Spectroscopic Data for S2**

^1^H NMR (400 MHz, CDCl_3_) δ 7.92 (d, *J* = 8.8 Hz, 2H), 7.79 (d, *J* = 8.7 Hz, 1H), 7.09 (d, *J* = 2.5 Hz, 1H), 7.00 (dd, *J* = 8.7, 2.6 Hz, 1H), 6.94 (d, *J* = 8.8 Hz, 2H), 4.27 – 4.16 (m, 4H), 3.98 – 3.92 (m, 4H), 3.90 (s, 3H), 3.87 (s, 3H), 2.55 (s, 3H); ^13^C NMR (126 MHz, CDCl_3_) δ 196.8, 168.7, 166.8, 162.6, 161.2, 135.6, 131.5, 130.5, 122.5, 116.3, 114.3, 114.2, 69.8, 69.7, 67.9, 67.6, 53.5, 52.8, 52.4, 26.4; MS (ESI) *m/z* 417 (M + H)^+^; HRMS (ESI) *m/z* calcd for C_22_H_25_O_8_ (M + H)^+^ 417.1544, found 417.1551.

To a solution of bis-ester **S2** (55 mg, 0.13 mmol) in THF (4 mL), was added DIBAL-H (1M in hexane, 1.06 mL, 1.06 mmol). The solution was stirred at room temperature for 24 h and 50 ^°^C for 2 h, until LC-MS analysis indicated full reduction of the ester groups to the aldehyde, and reduction of the ketone to the 2^°^ alcohol. The reaction was quenched with sodium potassium tartrate (5 mL) and extracted with ethyl acetate. The organic layer was concentrated *in vacuo* and dissolved in CH_2_Cl_2_ (9 mL). Dess-Martin periodinane (360 mg, 0.85 mmol) was added, and the white suspension was stirred at room temperature for 12 h until LC-MS analysis indicated oxidation of the 2^°^ alcohol to the ketone. The reaction was quenched with sat. aq. Na_2_S_2_O_3 ­_and NaHO_3_ and extracted with CH_2_Cl_2_. The organic phase was concentrated in *vacuo* and purified by flash column chromatography (20% Ethyl acetate in petroleum ether) to afford the product **S3** as a white solid (31 mg, 65% yield) over two steps.

Supplementary Figure 24. **Spectroscopic Data for S3**

^1^H NMR (400 MHz, CDCl_3_) $\delta$ 10.64 (s, 1H), 10.32 (s, 1H), 7.93-7.90 (m, 3H), 7.47 (d, *J* = 2.5 Hz, 1H), 7.24 (dd, *J* = 8.5, 2.5 Hz, 1H), 6.94 (d, *J* = 8.8 Hz, 2H), 4.31 (t, *J* = 4.6 Hz, 2H), 4.23 (t, *J* = 4.6 Hz, 2H), 4.01-3.94 (m, 4H), 2.55 (s, 3H); ^13^C NMR (100 MHz, CDCl_3_) $\delta$ 196.7, 191.8, 190.1, 163.1, 162.6, 138.6, 134.6, 130.6 (x2), 129.7, 119.3, 115.3, 114.3, 69.9, 69.7, 68.2, 67.6, 26.3; MS (ESI) *m/z* 357 (M + H)^+^; HRMS (ESI) *m/z* calcd for C_20_H_21_O_7_ (M + H)^+^ 357.1333, found 357.1335.

Selenium dioxide (17 mg,0.15 mmol) was added to a solution of ketone **S3** (18 mg, 0.05 mmol) in 1,4-dioxane (200 μL) and H_2_O (20 μL) in a microwave vessel. The solution was heated using microwave irradiation to a set temperature of 100 ^°^C for 1 h, using a max. power of 100 W. After reaction completion (determined by HPLC-MS), the dark-green mixture was cooled to room temperature, filtered to remove Se and excess SeO_2_, and washed with 1,4-dioxane (0.5 mL). H_2_O (0.5 mL) was added to the filtrate, ensuring that the solution remained homogeneous. The mixture was purified by reverse-phase C18 column chromatography using the CombiFlash automatic chromatography system (Gradient: 100:0 H_2_O:MeCN (0 →3 mins); 100:0 → 85:15 H_2_O:MeCN (3 →12 mins); 85:15 H_2_O:MeCN (12 →14 mins);85:15 → 80:20 H_2_O:MeCN (14→20 mins)). The pure product **KArGO** could be obtained as a white solid (12 mg, 62% yield) after removal of H_2_O by freeze-drying.

Supplementary Figure 25. **Spectroscopic Data for KArGO**

^1^H NMR (500 MHz, CD_3_CN) $\delta$ 10.48 (s, 1H), 10.28 (s, 1H), 8.04 (d, *J* = 9.0 Hz, 2H), 7.97 (d, *J* = 8.5 Hz, 1H), 7.43 (d, *J* = 2.6 Hz, 1H), 7.31 (dd, *J* = 8.5, 2.6 Hz, 1H), 7.02 (d, *J* = 9.0 Hz, 2H), 5.84 (s, 1H), 4.30 (t, *J* = 4.3 Hz, 2H), 4.23 (t, *J* = 4.3 Hz, 2H), 4.03 (m, 4H); ^13^C NMR (125 MHz, CD_3_CN) $\delta$ 194.6, 193.1, 192.0, 163.4, 163.2, 138.9, 134.0, 132.0, 126.2, 119.1, 115.7, 114.5, 87.0, 69.2, 69.1, 68.33, 67.8 (missing 1 13C peak); MS (ESI) *m/z* 371 (M – H_2_O + H)^+^; HRMS (ESI) *m/z* calcd for C_20_H_19_O_7_ (M – H_2_O + H)^+^ 371.1125, found 371.1127.

**Supplementary Note 7. Isolation and Characterisation of Arginine Ligation Adducts**

*N*-Acetyl arginine methyl ester (571 mg, 2.5 mmol) was dissolved in 200 mM boric acid (50 mL) and the pH was adjusted to pH 8 with 2M NaOH. *p*-OMe-Phenyl glyoxal (300 mg, 1.65 mmol) was dissolved in 15 mL MeCN and 10 mL 100 mM borate buffer pH 8. The glyoxal solution was added to the arginine solution, and the reaction mixture was stirred in a sealed flask for 1 h. Although LC-MS indicated incomplete conversion after 1 h, further reaction time resulted in formation of several side-products. Therefore, after 1 h, the mixture was concentrated *in vacuo* to a total volume of ~ 40 mL. The crude concentrate was purified by reverse-phase C18 column chromatography using the CombiFlash automatic chromatography system (eluting with 0 → 20 % MeCN in H_2_O + 0.1% TFA) to remove unreacted arginine and glyoxal monomers/oligomers. Product containing fractions were freeze-dried and further purified by preparative HPLC (eluting with 0 → 20 % MeCN in H_2_O + 0.1% TFA) to yield the desired product **3** (124 mg, 20%, 1:1 mixture of diastereomers), and **4** (31 mg, 5%) after lyophilisation.

Supplementary Figure 26. Arginine Ligation Adducts

**3**: White solid; ^1^H NMR (500 MHz, MeOD) δ 7.29 (*app* dd, *J* = 8.8, 2.5 Hz, 2H), 7.00 (d, *J* = 8.8 Hz, 2H), 5.49 (s, 0.12H), 5.35 (s, 0.88H), 4.49-4.44 (m, 1H), 3.81 (s, 3H), 3.77-3.67 (m, 2H), 3.69 (*app* d, 3H), 1.98 (*app* d, 3H), 1.90-1.81 (m, 1H), 1.76-1.64 (m, 3H); ^13^C NMR (125 MHz, MeOD) δ 172.18, 172.09, 172.07, 160.73, 158.62, 128.06, 124.63, 114.29, 61.08, 54.46, 51.67 (51.61), 51.41, 39.10 (39.01), 28.22 (28.17), 23.80 (23.75), 20.92; HRMS (ESI) *m/z* calcd for C_18_H_24_N_4_O_5_ (M + H)^+^ 377.1825, found 377.1805.

**4**: White solid; ^1^H NMR (500 MHz, MeOD) δ 7.99 (d, *J* = 8.6 Hz, 2H), 7.08 (d, *J* = 8.6 Hz, 2H), 4.46-4.41 (m, 1H), 3.89 (s, 3H), 3.48-3.41 (m, 2H), 2.02 (s, 3H), 1.97-1.91 (m, 1H) 1.86-1.73 (m, 2H); ^13^C NMR (126 MHz, MeOD) δ 172.39, 167.97, 167.75, 167.72, 164.59, 154.48, 130.21, 123.11, 113.97, 54.84, 40.71, 35.56, 28.34, 24.22, 20.98; HRMS (ESI) *m/z* calcd for C_17_H_20_N_4_O_5_ (M + H)^+^ 360.1434, found 360.1446.

Supplementary Figure 27. **Assigned Experimental NMR Chemical Shifts Products 3 and 4**

- 1. **Characteristics of NMR Spectra of 3**

In MeOD-d_4_, **3** exists as an 88:12 mixture of guanidine double bond stereoisomers, observed by two separate peaks for *H*4 at 5.49 and 5.35 ppm.

**3** is also present as a 1:1 mixture of diastereomers. Signals for *H*2’, *H*6’’, *H*8’’ and *C*1’’-*C*4’’ are split to apparent doublets. The diastereomer peaks in the ^13^C NMR spectrum of3 are given in parentheses.

The acidic proton *H*4 undergoes H-D exchange in MeOD-d_4_, as observed by a decrease in the integral of the signal at 5.35 ppm:


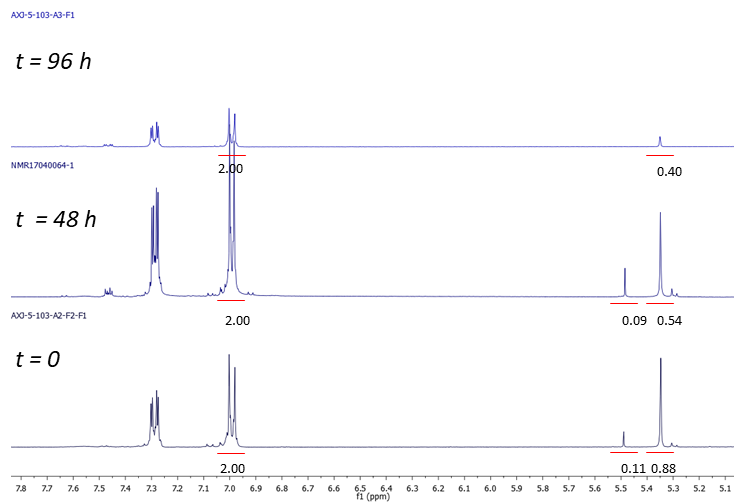


**Supplementary Note 8. General Strategy for Synthesis of ArGO Compounds**

Supplementary Figure 28. **Summary of ArGO Analogue Structures**

The ArGO compounds were synthesised by selenium-dioxide mediated oxidation of a bis-acetophenone precursor, followed by reflux in water to convert the aldehyde to the crystalline hydrate form.

Supplementary Figure 29. Synthetic Procedures of **ArGO Compounds**

**Supplementary Note 9. Strategy for Synthesis of bis-Acetophenone (­pre-ArGO) Compounds**

A mixture of the requisite hydroxyacetophenone (3 mmol), dihalide linker (1 mmol) and Cs_2_CO_3_ (1.63 g, 5mmol) in MeCN (6 mL) were heated overnight in a sealed flask at 80 ^°^C. After cooling to room temperature, the solvent was evaporated *in vacuo*. The concentrated material was dissolved in EtOAc and extracted with 1 M NaOH and brine. The organic layer was dried over Na_2_SO_4_, filtered, and the solvent was evaporated. The crude product was purified by flash column chromatography (gradient of 80:20 to 50:50 petroleum ether:EtOAc).

Supplementary Figure 30. Synthesis of bis-acetophenone Compounds with Ether Linkers

Supplementary Figure 31. Synthesis of bis-acetophenone Compounds with Amide Linkers

EDCI.HCl (461 mg, 2.4 mmol) and NEt_3_ (0.826 mL, 5 mmol) were added to a solution of *p*-acetylbenzoic acid (492 mg, 3mmol), diamine (1 mmol) and HOBt (324 mg, 2.4 mmol) in dry DMF. The homogeneous solution was stirred at room temperature under argon for 24 h. The reaction mixture was filtered to remove NEt_3_.HCl. The filtrate was concentrated under reduced pressure and re-dissolved in CH_2_Cl_2._  Pre-amide-ArGO-2 was insoluble in CH_2_Cl_2_ and could be obtained pure directly by filtration and washing with CH_2_Cl_2._ For pre-amide-ArGO-1 , unreacted carboxylic acid and urea by-products were removed from the CH_2_Cl_2_ solution by extraction with 20% NaHCO_3_ (aq.) and brine. The organic layer was dried over Na_2_SO_4_, filtered, and the solvent was evaporated. The crude product was purified by flash column chromatography (gradient of 50:50 to 0:100 petroleum ether: EtOAc).

**Supplementary Note 10**. **General Procedure for SeO_2_ Oxidation**

According to a modified literature procedure,^4^ SeO_2_ (60 mg, 0.54 mmol, 5.4 eq.) was added to a solution of bis-acetophenone (0.1 mmol) in 450 μL:50 μL 1,4-dioxane:H_2_O. The clear mixture was heated using microwave irradiation to a set temperature of 100 ^°^C for 0.5-1 h, using a max. power of 150 W. After reaction completion (determined by HPLC-MS), the dark-green mixture was cooled to room temperature, filtered to remove Se and excess SeO_2_, and washed with 1,4-dioxane (0.5-1 mL). The solvent was concentrated under reduced pressure to a final volume of 0.3-0.5 mL [complete evaporation caused polymerisation of the product]. H_2_O (2 mL) was then added, the mixture was refluxed at 100 ^°^C for 0.5 h, filtered hot to remove further insoluble precipitates, and washed with hot water. The aqueous filtrate was cooled to 0 ^°^C. If product crystals precipitated from solution (OMe-ArGO1, *meta*-ArGO1), these were collected by filtration, washed with water and dried under vacuum. Alternatively, the product was purified by reverse-phase chromatography.

The solution obtained from hot filtration was concentrated under reduced pressure to a total volume of ~ 2 mL. MeCN (~0.5 mL) was added to ensure homogeneity, and the mixture was purified by reverse-phase C18 column chromatography using the CombiFlash automatic chromatography system (Gradient: 100:0 H_2_O:MeCN (0 →3 mins); 100:0 → 85:15 H_2_O:MeCN (3 →12 mins); 85:15 H_2_O:MeCN (12 →14 mins);85:15 → 80:20 H_2_O:MeCN (14→20 mins)). The pure product could be obtained after removal of H_2_O by freeze-drying.

**Supplementary Note 11. Synthesis of BDG**

According to the literature procedure,^3^ HBr (0.68 mL, 6mmol) was added to a solution of bis-acetophenone (1 mmol) in DMSO dropwise. The clear mixture was heated to 60 ^°^C for 18 h. After reaction completion (determined by HPLC-MS), the dark-green mixture was cooled to room temperature, poured into ice and filtered to get the precipitate, which was used directly.

**Supplementary Note 12.** **Spectroscopic Data of Bis Acetophenone (pre-ArGO) Compounds**

**pre-OMe-ArGO1**

Supplementary Figure 32. **Spectroscopic Data of pre-OMe-ArGO1**

White solid; 251 mg, 70% yield from 1 mmol dibromoethane and 3 mmol 4-hydroxy-3-methoxyacetophenone: m.p. 92-94 $℃$; IR (neat) 1673, 1592, 1269 cm^-1^; ^1^H NMR (400 MHz, (CD_3_)_2_SO) $\delta$ 7.64 (dd, *J* = 8.4, 1.7 Hz, 2H), 7.46 (d, *J* = 1.6 Hz, 2H), 7.16 (d, *J* = 8.4 Hz, 2H), 4.44 (s, 4H), 3.81 (s, 6H), 2.54 (s, 6H); ^13^C NMR (100 MHz CDCl_3_)$\delta$26.4, 56.2, 67.4, 110.8, 112.2, 123.2, 131.2, 149.5, 152.4, 196.9; MS (ESI) *m/z* 359 (M + H)^+^; HRMS (ESI) *m/z* calcd for C_20_H_23_O_6_ (M + H)^+^ 359.14892, found 359.14914.

**pre-OMe-ArGO2**

Supplementary Figure 33. **Spectroscopic Data of pre-OMe-ArGO2**

White solid; 340 mg, 84% yield from 1 mmol 2,2’-diiodoethyl ether and 3 mmol 4-hydroxy-3-methoxyacetophenone: m.p. 85-87 $℃$; IR (neat) 1672, 1592, 1269 cm^-1^; ^1^H NMR (400 MHz, CD_3_Cl) $\delta$ 7.51 (d, *J* = 8.1 Hz, 2H), 7.50 (s, 2H), 6.90 (d, *J* = 8.1 Hz, 2H), 4.26 (t, *J* = 4.9 Hz, 4H), 3.98 (t, *J* = 4.9 Hz, 4H), 3.89 (s, 6H), 2.55 (s, 6H); ^13^C NMR (100 MHz CDCl_3_)$\delta$26.3, 56.1, 68.6, 69.9, 110.6, 111.8, 123.2, 130.8, 149.4, 152.7, 196.9; MS (ESI) *m/z* 403 (M + H)^+^; HRMS (ESI) *m/z* calcd for C_22_H_27_O_7_ (M + H)^+^ 403.17513, found 403.17539.

**pre-*meta*-ArGO1**

Supplementary Figure 34. **Spectroscopic Data of pre-*meta*-ArGO1**

White solid; 234 mg, 73% yield from 1 mmol dibromoethane and 3 mmol 3-hydroxyacetophenone: m.p.122-124 $℃$; IR (neat) 1680, 1600, 1269, 1066 cm^-1^; ^1^H NMR (400 MHz, CD_3_Cl) $\delta$ 7.58-7.55 (m, 4H), 7.41 (t, *J* = 7.9 Hz, 2H), 7.18 (dd, *J* = 8.2, 2.5 Hz, 2H), 4.41 (s, 4H), 2.60 (s, 6H); ^13^C NMR (100 MHz CDCl_3_)$\delta$26.9, 66.8, 113.3, 120.5, 121.8, 129.8, 138.7, 158.9, 198.0; MS (ESI) *m/z* 321 (M + Na)^+^; HRMS (ESI) *m/z* calcd for C_18_H_18_NaO_4_ (M + Na)^+^ 321.10973, found 321.10971.

**pre-*meta*-ArGO2**

Supplementary Figure 35. **Spectroscopic Data of pre-meta-ArGO2**

White solid; 256 mg, 75% yield from 1 mmol 2,2’-diiodoethyl ether and 3 mmol 3-hydroxyacetophenone: m.p. 65-67 $℃$; IR (neat) 1672, 1592, 1269 cm^-1^; ^1^H NMR (400 MHz, CD_3_Cl) $\delta$ 7.55-7.51 (m, 4H), 7.36 (t, *J* = 7.9 Hz, 2H), 7.14 (dd, *J* = 8.2, 2.2 Hz, 2H), 4.22 (t, *J* = 4.7 Hz, 4H), 3.96 (t, *J* = 4.7 Hz, 4H), 2.59 (s, 6H); ^13^C NMR (100 MHz CDCl_3_)$\delta$26.9, 67.8, 70.0, 113.4, 120.3, 121.5, 129.7, 138.6, 159.1, 198.1; MS (ESI) *m/z* 343 (M + H)^+^; HRMS (ESI) *m/z* calcd for C_20_H_23_O_5_ (M + H)^+^ 343.15400, found 343.15474.

**pre-*ortho*-ArGO-1**

Supplementary Figure 36. **Spectroscopic Data of pre-*ortho*-ArGO-1**

White solid; 138 mg, 46% yield from 1 mmol dibromoethane and 3 mmol 2-hydroxyacetophenone: m.p. 133-135 $℃$; IR (neat) 1667, 1294, 1235 cm^-1^; ^1^H NMR (400 MHz, CDCl_3_) $\delta$ 7.74 (dd, *J* = 7.7, 1.8 Hz, 2H), 7.48 (td, *J =* 7.7, 1.8 Hz, 2H), 7.05 (td, *J* = 7.5, 0.8 Hz, 2H), 7.00 (d, *J* = 8.3 Hz, 2H), 4.50 (s, 4H), 2.58 (s, 6H); ^13^C NMR (100 MHz CDCl_3_)$\delta$32.0, 67.0, 112.5, 121.5, 128.9, 130.7, 133.8, 157.6, 199.7; MS (ESI) *m/z* 299 (M + H)^+^ , 321 (M + Na)^+^; HRMS (ESI) *m/z* calcd for C_18_H_19_O_4_ (M + H)^+^ 299.12779, found 299.12755.

**pre-Amide-ArGO-1**

Supplementary Figure 37. **Spectroscopic Data of pre-Amide-ArGO-1**

White solid; 357 mg, 94% yield from 1 mmol *N,N’-*dimethylethylene diamine and 3 mmol 4-acetylbenzoic acid: m.p. 139-141 $℃$; IR (neat) 1685, 1633, 1402, 1267 cm^-1^; ^1^H NMR (400 MHz, CD_3_Cl) $\delta$ 7.94 (d, *J* = 8.3 Hz, 4H), 7.46 (d, *J* = 8.3 Hz, 4H), 3.92 (s, 4H), 3.08 (s, 6H), 2.59 (s, 6H); ^13^C NMR (100 MHz, (CD_3_)_2_SO)$\delta$26.8, 37.2, 44.0, 126.6, 128.3, 137.0, 141.0, 169.7, 197.5; MS (ESI) *m/z* 381 (M + H)^+^; HRMS (ESI) *m/z* calcd for C_22_H_25_N_2_O_4_ (M + H)^+^ 381.18088, found 381.18210.

**pre-Amide-ArGO-2**

Supplementary Figure 38. **Spectroscopic Data of pre-Amide-ArGO-2**

White solid; 372 mg, 94% yield from 1 mmol 1,5-diamino-3-oxapentane and 3 mmol 4-acetylbenzoic acid: m.p. 198-200 $℃$; IR (neat) 1679, 1634 cm^-1^; ^1^H NMR (400 MHz, DMSO-d_6_) $\delta$ 8.66 (t, *J* = 5.8 Hz, 2H), 7.98 (d, *J* = 8.4 Hz, 4H), 7.92 (d, *J* = 8.4 Hz, 4H), 3.59 (t, *J* = 5.8 Hz, 4H), 3.46 (q, *J* = 5.8 Hz, 4H), 2.61 (s, 6H); ^13^C NMR (100 MHz DMSO-d_6_)$\delta$26.9, 68.5, 127.5, 128.0, 138.2, 138.5, 165.6, 197.7 [H_2_C-NH not observed- overlapped with DMSO multiplet]; MS (ESI) *m/z* 397 (M + H)^+^ , 419 (M + Na)^+^; HRMS (ESI) *m/z* calcd for C_22_H_25_N_2_O_5_ (M + H)^+^ 397.17580, found 397.17568.

**Supplementary Note 13. Spectroscopic Data of ArGO Analogues**

The ArGO compounds exist as an equilibrium between hydrate and aldehyde forms. Resonances for both forms are given separately in the ^1^H NMR data. The peaks for the aldehyde form are given in the ^13^C NMR data where possible. The characteristic ^13^C splitting pattern for the hydrate-aldehyde equilibrium is identified by three peaks between 180-200 ppm, corresponding to the ketone group for hydrate and aldehyde forms; and an aldehyde peak.

**BDG**

Supplementary Figure 39. **Spectroscopic Data of BDG**

White solid: m.p.166-168 $℃$; IR (neat) 3500-3200 (br), 1695, 760 cm^-1^; Ratio of hydrate: aldehyde =0.97: 0.03; ^1^H NMR for hydrate (400 MHz, (CD_3_)_2_SO) $\delta$ 8.15 (d, *J* = 8.3 Hz, 4H), 7.87 (d, *J* = 8.3 Hz, 4H), 5.67 (s, 2H); ^13^C NMR (100 MHz (CD_3_)_2_SO)$\delta$ 89.5, 127.1, 130.2, 133.3, 143.4, 195.9; MS (ESI) *m/z* 325 (M + Na)^+^; HRMS (ESI) *m/z* calcd for C_17_H_17_O_6_ (M + MeOH+H)^+^ 317.10251, found 317.24108.

**OMe-ArGO1**

Supplementary Figure 40. **Spectroscopic Data of OMe-ArGO1**

White solid; 18 mg, 42% yield from 0.1 mmol pre-OMe-ArGO-1: m.p.214-216 $℃$; IR (neat) 3600-3000 (br), 1450, 1268 cm^-1^; Ratio of hydrate: aldehyde =0.92: 0.08; ^1^H NMR for hydrate (400 MHz, (CD_3_)_2_SO) $\delta$ 7.78 (d, *J* = 8.5 Hz, 2H), 7.56 (s, 2H), 7.18 (d, *J* = 8.5 Hz, 2H), 6.63 (d, *J* = 7.4 Hz, 4H), 5.68 (t, *J* = 6.5 Hz, 2H), 4.45 (s, 4H), 3.81 (s, 6H); ^1^H NMR for aldehyde (400 MHz, (CD_3_)_2_SO) $\delta$ 9.59 (s, 2H), 7.78 (d, *J* = 8.5 Hz, 2H), 7.56 (s, 2H), 7.18 (d, *J* = 8.5 Hz, 2H), 4.45 (s, 4H), 3.81 (s, 6H); ^13^C NMR (100 MHz (CD_3_)_2_SO)$\delta$ 55.5, 67.1, 88.8, 111.8, 112.0, 124.3, 126.7, 148.2, 152.2, 194.8; MS (ESI) *m/z* 445 (M + Na)^+^; HRMS (ESI) *m/z* calcd for C_20_H_22_NaO_10_ (M + Na)^+^445.11052, found 445.11038.

**OMe-ArGO2**

Supplementary Figure 41. **Spectroscopic Data of OMe-ArGO2**

White solid; 26 mg, 56% yield from 0.1 mmol pre-OMe-ArGO-2: m.p.75-77 $℃$; IR (neat) 3600-3100 (br), 1658, 1267 cm^-1^; Ratio of hydrate: aldehyde =0.49: 0.51; ^1^H NMR for hydrate (400 MHz, (CD_3_)_2_SO) $\delta$ 7.75 (d, *J* = 8.3 Hz, 2H), 7.55 (s, 2H), 7.09 (d, *J* = 8.5 Hz, 2H), 6.61 (d, *J* = 6.5 Hz, 4H), 5.67 (t, *J* = 7.5 Hz, 2H), 4.21 (t, *J* = 4.3 Hz, 4H), 3.91-3.85 (m, 4H), 3.80 (s, 6H); ^1^H NMR for aldehyde (400 MHz, (CD_3_)_2_SO) $\delta$ 9.57 (s, 2H), 7.80 (d, *J* = 8.4 Hz, 2H), 7.55 (s, 2H), 7.17 (d, *J* = 8.5 Hz, 2H), 4.25 (t, *J* = 4.3 Hz, 4H), 3.91-3.85 (m, 4H), 3.82 (s, 6H); ^13^C NMR (100 MHz (CD_3_)_2_SO)$\delta$ 55.6, 68.0, 68.9, 88.7, 111.7, 112.0, 124.3, 126.5, 148.5, 153.7, 194.7; MS (ESI) *m/z* 489 (M + Na)^+^; HRMS (ESI) *m/z* calcd for C_22_H_26_NaO_11_ (M + Na)^+^489.13673, found 489.13729.

**HP-ArGO**

Supplementary Figure 42. **Spectroscopic Data of HP-ArGO**

White solid; 33 mg, 83% yield from 0.1 mmol 1,5-bis(4-acetylphenoxy)pentane: m.p.97-99 $℃$; IR (neat) 3600-3000 (br), 1682, 1603, 1033 cm^-1^; Ratio of hydrate: aldehyde =0.58: 0.42; ^1^H NMR for hydrate (400 MHz, (CD_3_)_2_SO) $\delta$ 8.04 (d, *J* = 8.8 Hz, 4H), 7.04 (d, *J* = 8.8 Hz, 4H), 6.62 (d, *J* = 7.3 Hz, 4H), 5.65 (t, *J* = 7.0 Hz, 2H), 4.21-4.00 (m, 4H), 1.89-1.75 (m, 4H), 1.67-1.49 (m, 2H); ^1^H NMR for aldehyde (400 MHz, (CD_3_)_2_SO) $\delta$ 9.53 (s, 2H), 8.07 (d, *J* = 8.9 Hz, 4H), 7.11 (d, *J* = 8.8 Hz, 4H), 4.21-4.00 (m, 4H), 1.89-1.75 (m, 4H), 1.67-1.49 (m, 2H); ^13^C NMR for hydrate (100 MHz (CD_3_)_2_SO)$\delta$22.0, 28.2, 67.7, 89.0, 114.1, 124.6, 131.7, 162.2, 186.6; ^13^C NMR for aldehyde (100 MHz (CD_3_)_2_SO)$\delta$22.0, 28.2, 68.0, 114.7, 126.2, 132.6, 163.7, 190.2, 194.6; MS (ESI) *m/z* 427 (M + Na)^+^; HRMS (ESI) *m/z* calcd for C_21_H_24_NaO_8_ (M + Na)^+^427.13634, found 427.13620.

***meta*-ArGO1**

Supplementary Figure 43. **Spectroscopic Data of *meta*-ArGO1**

White solid; 27 mg, 76% yield from 0.1 mmol pre-*m*-ArGO-1: m.p.115-117 $℃$; IR (neat) 3500-3100 (br), 1698, 1587, 1044 cm^-1^; Ratio of hydrate: aldehyde =0.66: 0.34; ^1^H NMR for hydrate (400 MHz, (CD_3_)_2_SO) $\delta$ 7.68 (d, *J* = 7.8 Hz, 2H), 7.63 (s, 2H), 7.46 (t, *J* = 8.0 Hz, 2H), 7.28 (dd, *J* = 8.2, 2.2 Hz, 2H), 5.73-5.64 (m, 2H), 4.52-4.31 (m, 4H); ^1^H NMR for aldehyde (400 MHz, (CD_3_)_2_SO) $\delta$ 9.53 (s, 2H), 7.71 (d, *J* = 8.0 Hz, 2H), 7.64 (s, 2H), 7.53 (t, *J* = 8.0 Hz, 2H), 7.38 (dd, *J* = 7.9, 2,2 Hz, 2H), 4.52-4.31 (m, 4H); ^13^C NMR for hydrate (100 MHz (CD_3_)_2_SO)$\delta$ 66.5, 89.1, 115.0, 121.0, 122.9, 130.1, 135.1, 158.3, 189.2; ^13^C NMR for aldehyde (100 MHz (CD_3_)_2_SO)$\delta$66.7, 114.9, 119.6, 122.0, 129.7, 133.4, 158.2, 187.7, 195.9; MS (ESI) *m/z* 385 (M + Na)^+^; HRMS (ESI) *m/z* calcd for C_18_H_18_NaO_8_ (M + Na)^+^ 385.08939, found 385.09002.

***meta*-ArGO2**

Supplementary Figure 44. **Spectroscopic Data of *meta*-ArGO2**

White solid; 16 mg, 39% yield from 0.1 mmol pre-*m*-ArGO-2: m.p.59-61 $℃$; IR (neat) 3600-3000 (br), 1691, 1581, 1264 cm^-1^; Ratio of hydrate: aldehyde =0.82: 0.18; ^1^H NMR for hydrate (400 MHz, (CD_3_)_2_SO) $\delta$ 7.66 (d, *J* = 7.8 Hz, 2H), 7.59 (s, 2H), 7.43 (t, *J* = 7.9 Hz, 2H), 7.23 (dd, *J* = 8.2, 2.2 Hz, 2H), 6.74 (d, *J* = 3.8 Hz, 4H), 5.72-5.64 (m, 2H), 4.18 (t, *J* = 4.2 Hz, 4H), 3.85 (t, *J* = 4.2 Hz, 4H); ^1^H NMR for aldehyde (400 MHz, (CD_3_)_2_SO) $\delta$ 9.54 (s, 2H), 7.68 (d, *J* = 7.8 Hz, 2H), 7.59 (s, 2H), 7.50 (t, *J* = 7.9 Hz, 2H), 7.32 (dd, *J* = 8.2, 2.0 Hz, 2H), 4.18 (t, *J* = 4.2 Hz, 4H), 3.85 (t, *J* = 4.2 Hz, 4H); ^13^C NMR for hydrate (100 MHz (CD_3_)_2_SO)$\delta$ 67.3, 68.9, 89.1, 114.9, 119.5, 121.8, 129.6, 133.3, 158.3, 187.7; ^13^C NMR for aldehyde (100 MHz (CD_3_)_2_SO)$\delta$67.5, 69.0, 115.0, 121.0, 122.8, 130.1, 135.0, 158.5, 189.3, 195.9; MS (ESI) *m/z* 429 (M + Na)^+^; HRMS (ESI) *m/z* calcd for C_20_H_22_NaO_9_ (M + Na)^+^429.11560, found 429.11558.

***ortho*-ArGO1**

Supplementary Figure 45. **Spectroscopic Data of *ortho*-ArGO1**

White solid; 12 mg, 30% yield from 0.1 mmol pre-*o*-ArGO-1: m.p.144-146 $℃$; IR (neat) 3600-3100 (br), 1678 cm^-1^; Ratio of hydrate: aldehyde =0.97:0.03; ^1^H NMR for hydrate (400 MHz, (CD_3_)_2_SO) $\delta$ 7.59 (d, *J* = 7.6 Hz, 2H), 7.55 (t, *J* = 8.3 Hz, 2H), 7.22 (d, *J* = 8.3 Hz, 2H), 7.07 (t, *J* = 7.3 Hz, 2H), 6.34 (d, *J* = 8.2 Hz, 4H), 5.75 (t, *J* = 8.2 Hz, 2H), 4.45 (s, 4H); ^1^H NMR for aldehyde (400 MHz, (CD_3_)_2_SO) $\delta$ 9.72 (s, 2H), 7.60 (d, *J* = 7.6 Hz, 2H), 7.55 (t, *J* = 8.3 Hz, 2H), 7.22 (d, *J* = 8.3 Hz, 2H), 7.07 (t, *J* = 7.3 Hz, 2H), 4.45 (s, 4H); ^13^C NMR for hydrate (100 MHz (CD_3_)_2_SO)$\delta$ 67.6, 90.2, 114.0, 121.3, 126.7, 130.8, 134.1, 157.8, 199.7; MS (ESI) *m/z* 385 (M + Na)^+^, 399 (M + H+2H_2_O)^+^, 314 (M + Na+2MeOH)^+^; HRMS (ESI) *m/z* calcd for C_18_H_18_NaO_8_ (M + Na)^+^385.08939, found 385.08947.

**Amide-ArGO-1**

Supplementary Figure 46. **Spectroscopic Data of Amide-ArGO-1**

White solid; 16 mg, 36% yield from 0.1 mmol pre-amide-ArGO-1: m.p.132-134 $℃$; IR (neat) 1685, 1633 cm^-1^; Ratio of hydrate: aldehyde =0.91: 0.09; ^1^H NMR for hydrate (500 MHz, (CD_3_)_2_SO) $\delta$ 8.10 (d, *J* = 7.6 Hz, 4H), 7.46 (t, *J* = 8.0 Hz, 4H), 6.82 (d, *J* = 7.1 Hz, 4H), 5.67 (t, *J* = 7.1 Hz, 2H), 3.80 (s, 4H), 2.95 (s, 6H); ^1^H NMR for aldehyde (500 MHz, (CD_3_)_2_SO) $\delta$ 9.51 (s, 2H), 8.10 (d, *J* = 7.6 Hz, 4H), 7.46 (t, *J* = 8.0 Hz, 4H), 3.80 (s, 4H), 2.95 (s, 6H); ^13^C NMR for hydrate (125 MHz (CD_3_)_2_SO)$\delta$ 37.3, 44.1, 89.4, 126.4, 129.5, 134.0, 141.1, 169.8, 195.7; MS (ESI) *m/z* 445 (M + H)^+^ ,473 (M + H+2MeOH)^+^; HRMS (ESI) *m/z* calcd for C_22_H_25_N_2_O_8_ (M + H)^+^445.16054, found 445.16129.

**Amide-ArGO-2**

Supplementary Figure 47. **Spectroscopic Data of Amide-ArGO-2**

White solid; 25 mg, 54% yield from 0.1 mmol pre-amide-ArGO-1: m.p.117-118 $℃$; IR (neat) 1696, 1635 cm^-1^; Ratio of hydrate: aldehyde =0.97: 0.03; ^1^H NMR for hydrate (400 MHz, (CD_3_)_2_SO) $\delta$ 8.68 ((t, *J* = 5.0 Hz, 2H), 8.11 (d, *J* = 7.9 Hz, 4H), 7.94 (d, *J* = 7.9 Hz, 4H), 6.83 (d, *J* = 7.2 Hz, 4H), 5.68 (t, *J* = 7.0 Hz, 2H), 3.59 (t, *J* = 5.8 Hz, 4H), 3.46 (t, *J* = 5.8 Hz, 4H); ^13^C NMR for hydrate (100 MHz (CD_3_)_2_SO)$\delta$ 68.6, 89.5, 92.4, 127.0, 129.3, 135.0, 138.2, 165.5, 194.3; MS (ESI) *m/z* 461 (M + H)^+^, 483 (M + Na)^+^; HRMS (ESI) *m/z* calcd for C_22_H_25_N_2_O_9_ (M + H)^+^ 461.15565, found 461.15546.

**Supplementary Note 14. ^1^H and ^13^C NMR Spectra of New Compounds**

**
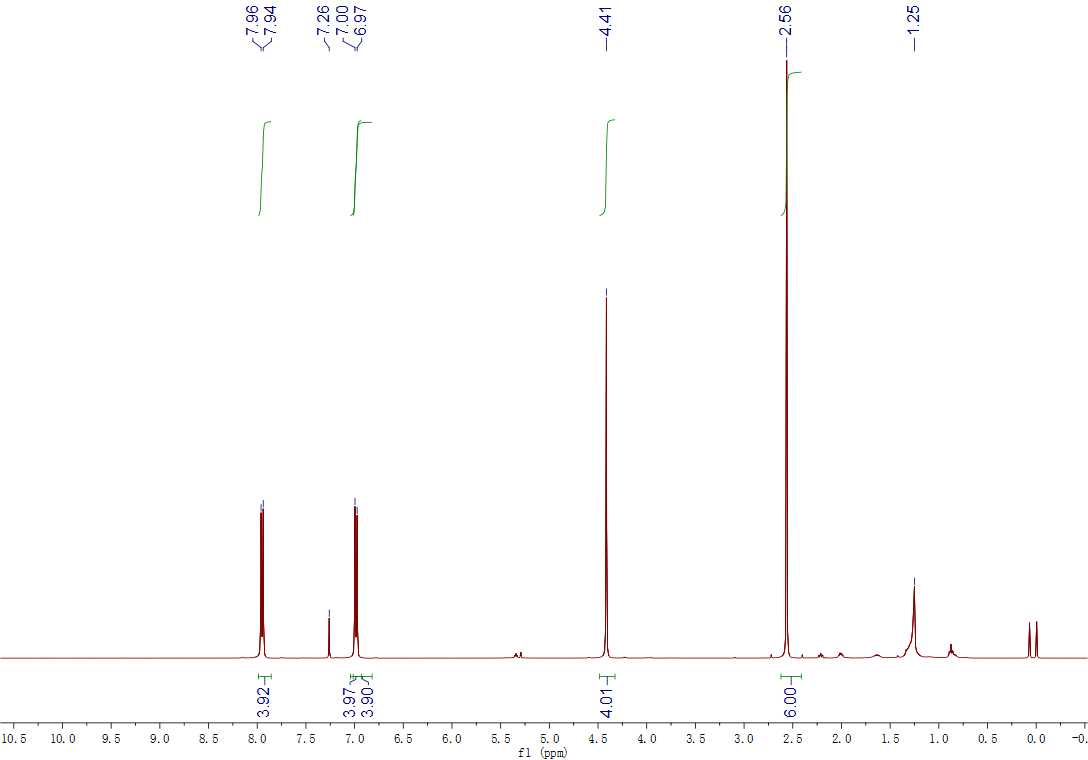
** Supplementary Figure 48. **^1^H NMR spectrum of pre-ArGO1 (400 MHz, CDCl_3_)**

**
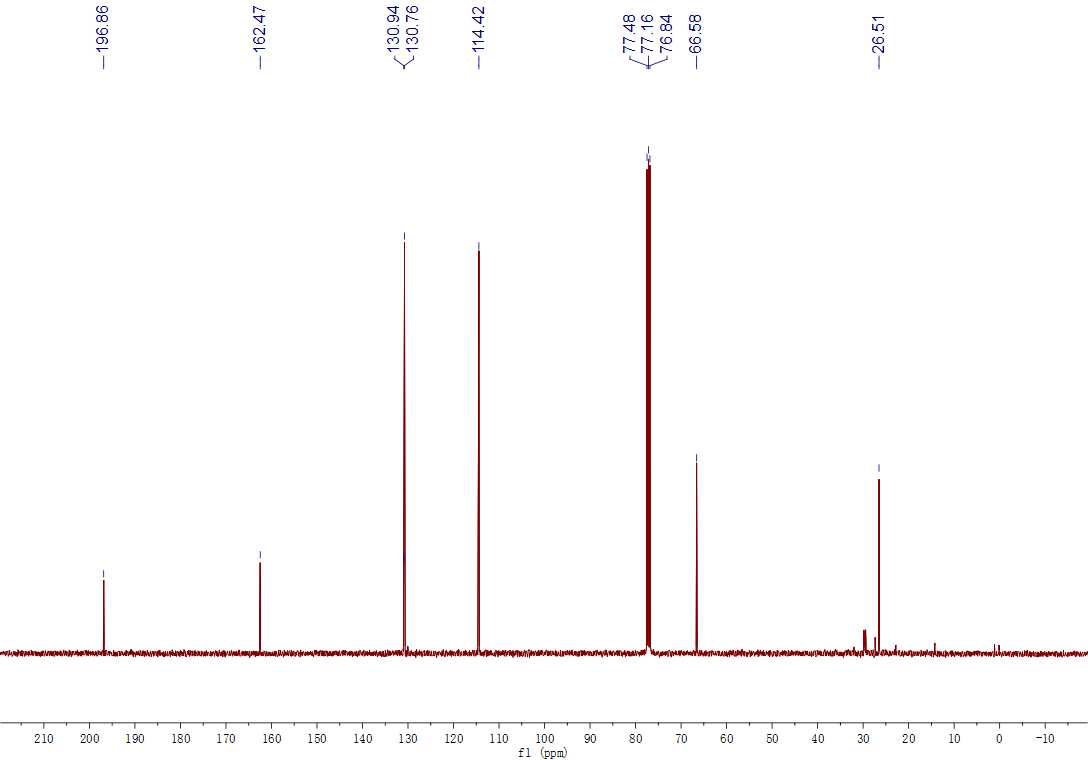
**

Supplementary Figure 49. **^13^C NMR spectrum of pre-ArGO-1 (100 MHz, CDCl_3_)**


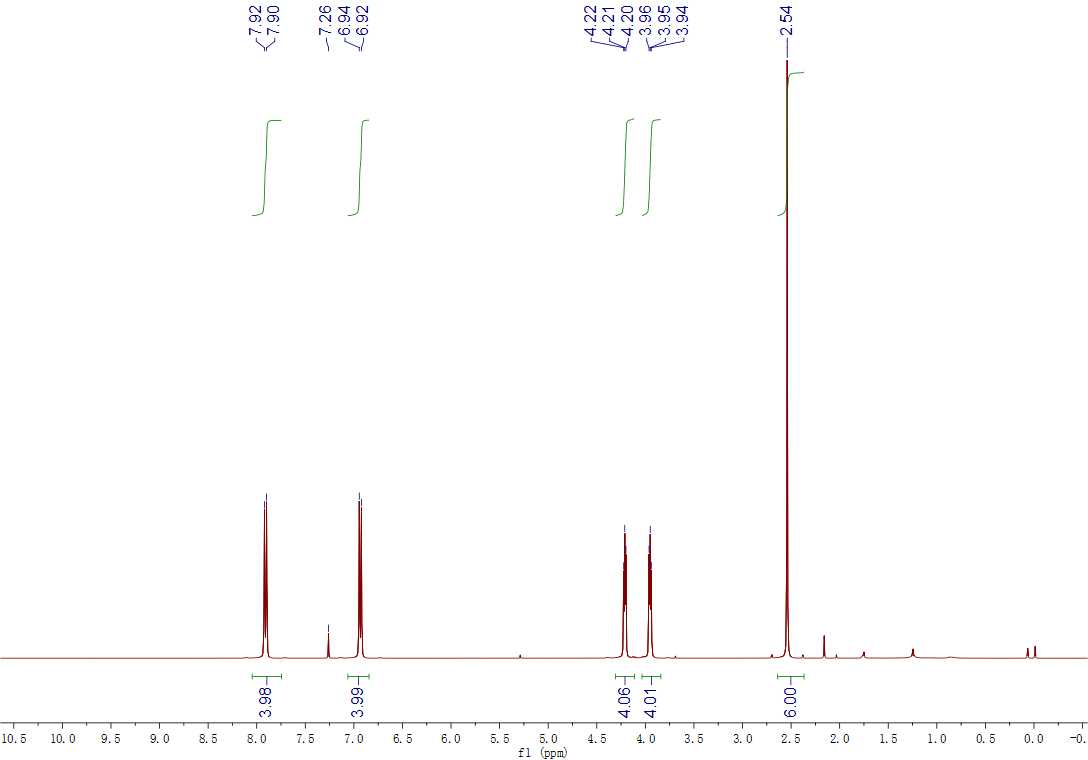
Supplementary Figure 50. **^1^H NMR spectrum of pre-ArGO2 (400 MHz, CDCl_3_)**


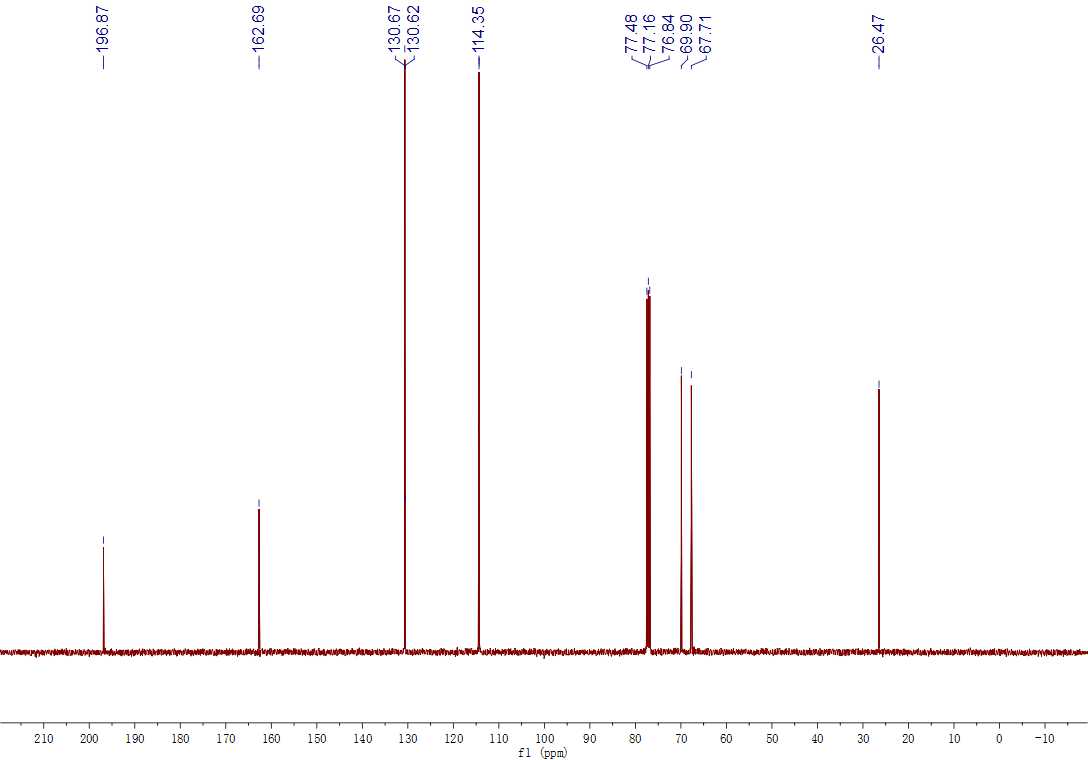


Supplementary Figure 51. **^13^C NMR spectrum of pre-ArGO2 (100 MHz, CDCl_3_)**


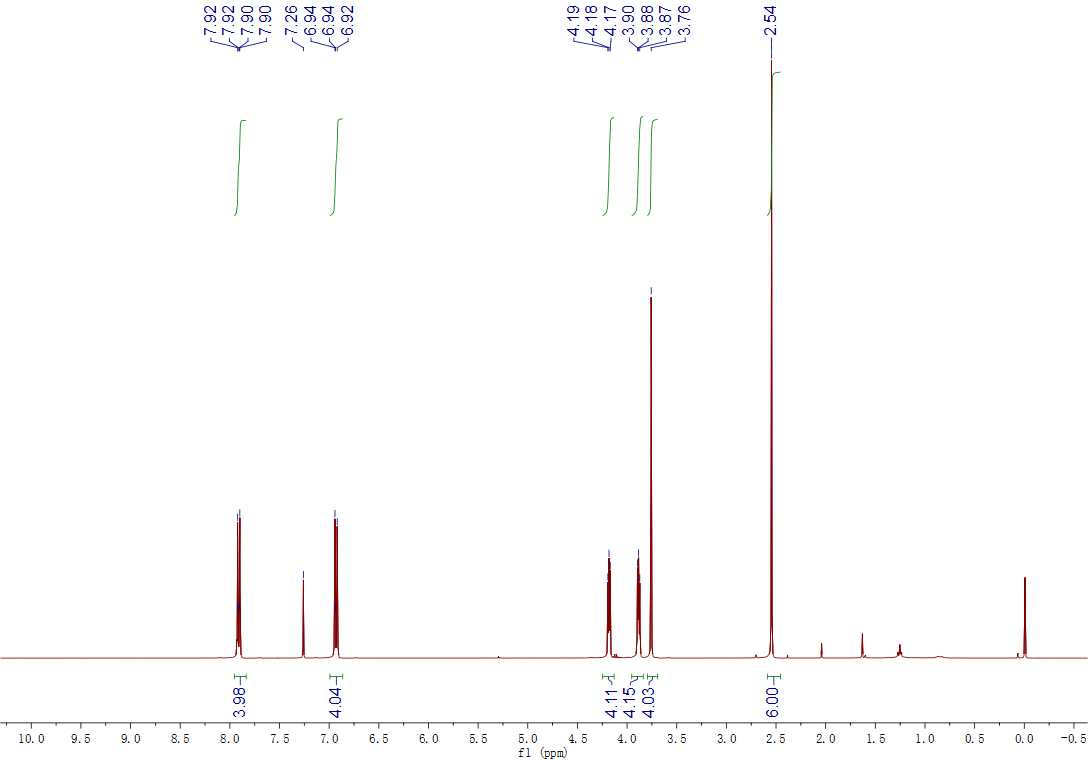
Supplementary Figure 52. **^1^H NMR spectrum of pre-ArGO3 (400 MHz, CDCl_3_)**


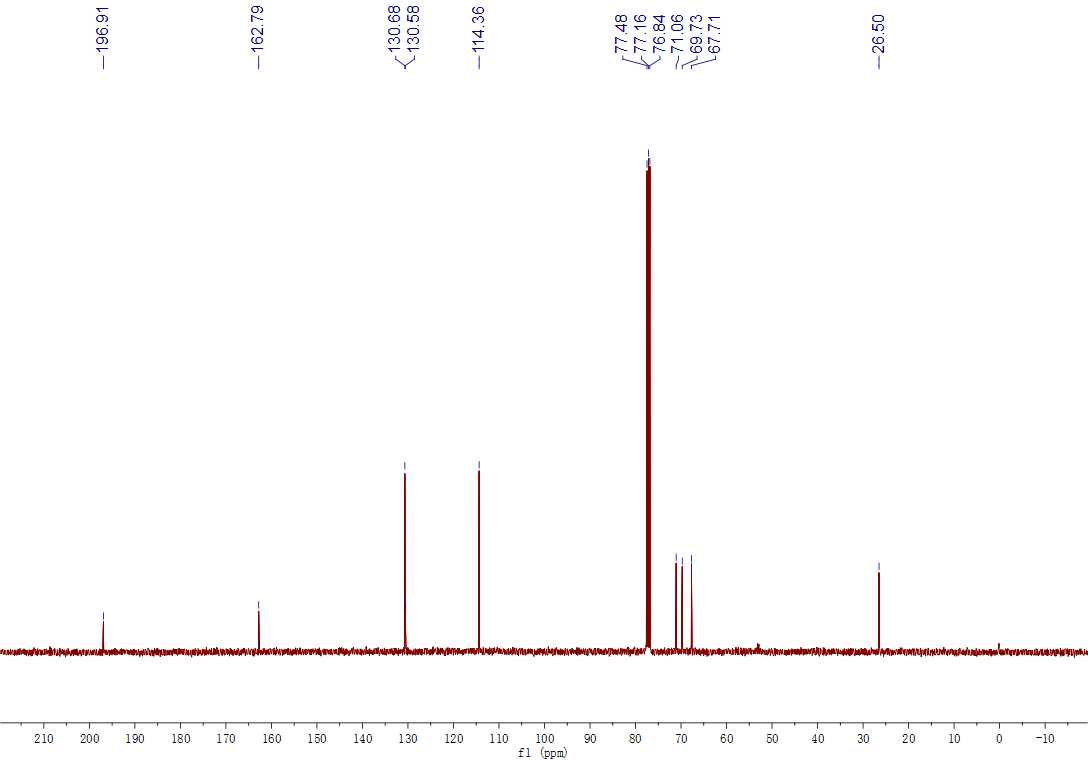
Supplementary Figure 53. **^13^C NMR spectrum of pre-ArGO3 (100 MHz, CDCl_3_)**


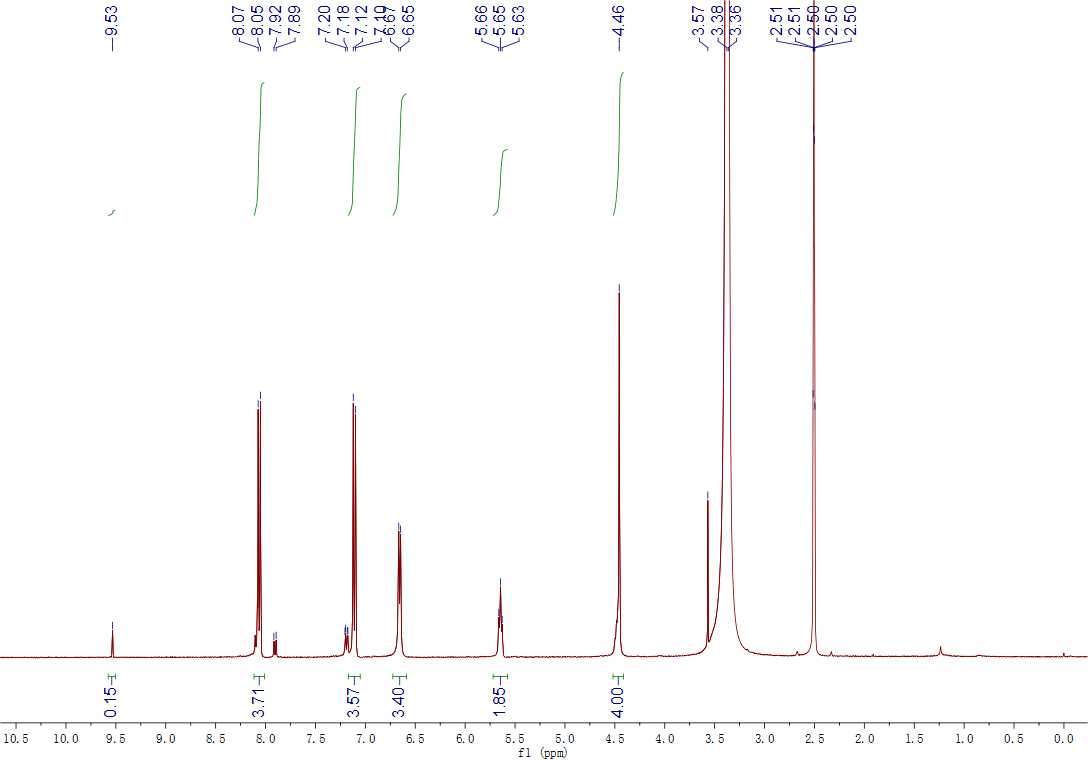


Supplementary Figure 54. **^1^H NMR spectrum of ArGO1 (400 MHz, (CD_3_)_2_SO)**


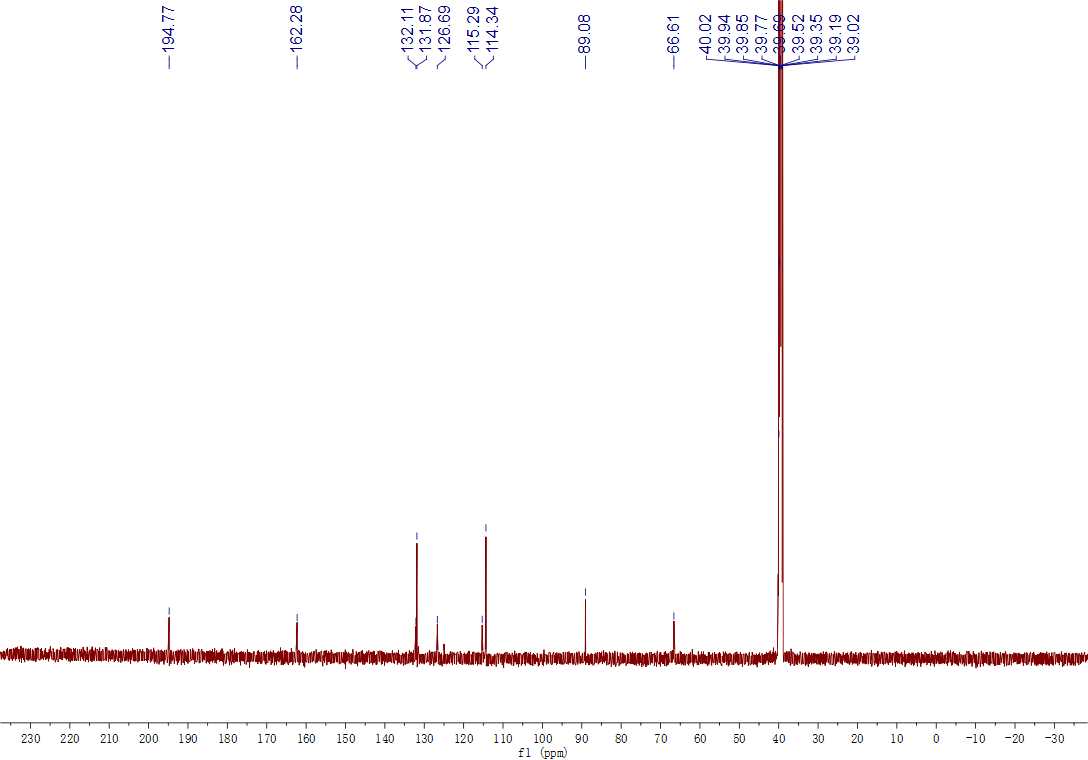


Supplementary Figure 55. **^13^C NMR spectrum of ArGO1 (100 MHz, (CD_3_)_2_SO)**


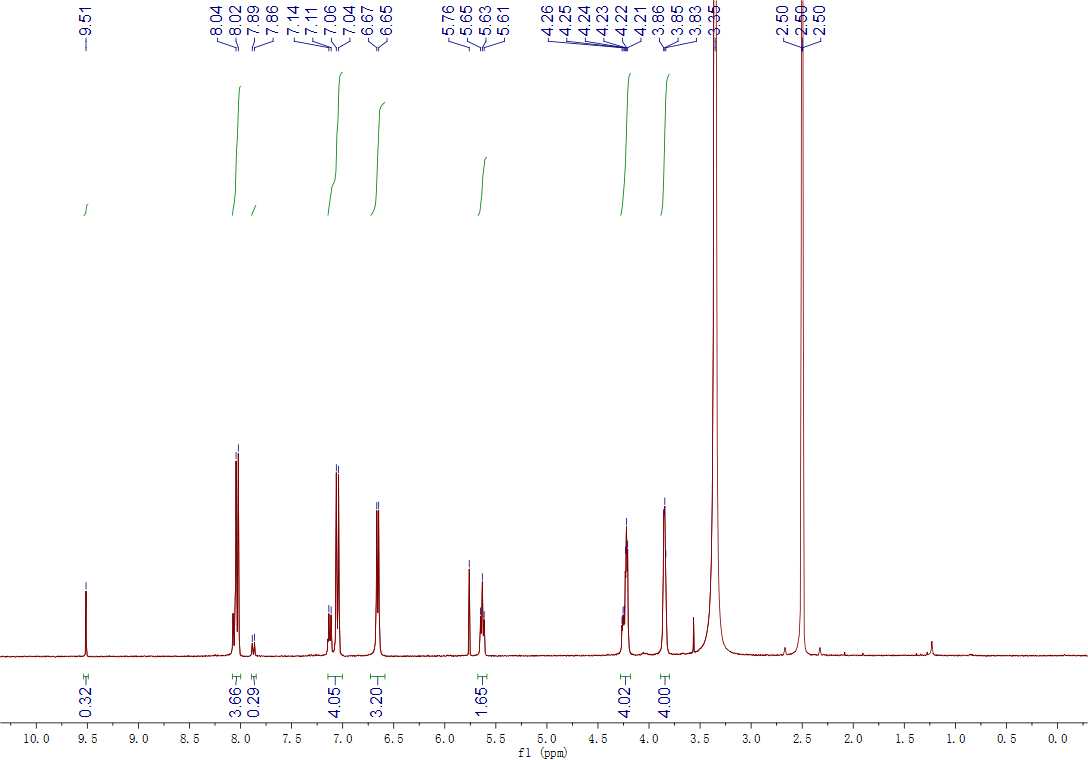


Supplementary Figure 56. **^1^H NMR spectrum of ArGO2 (400 MHz, (CD_3_)_2_SO)**


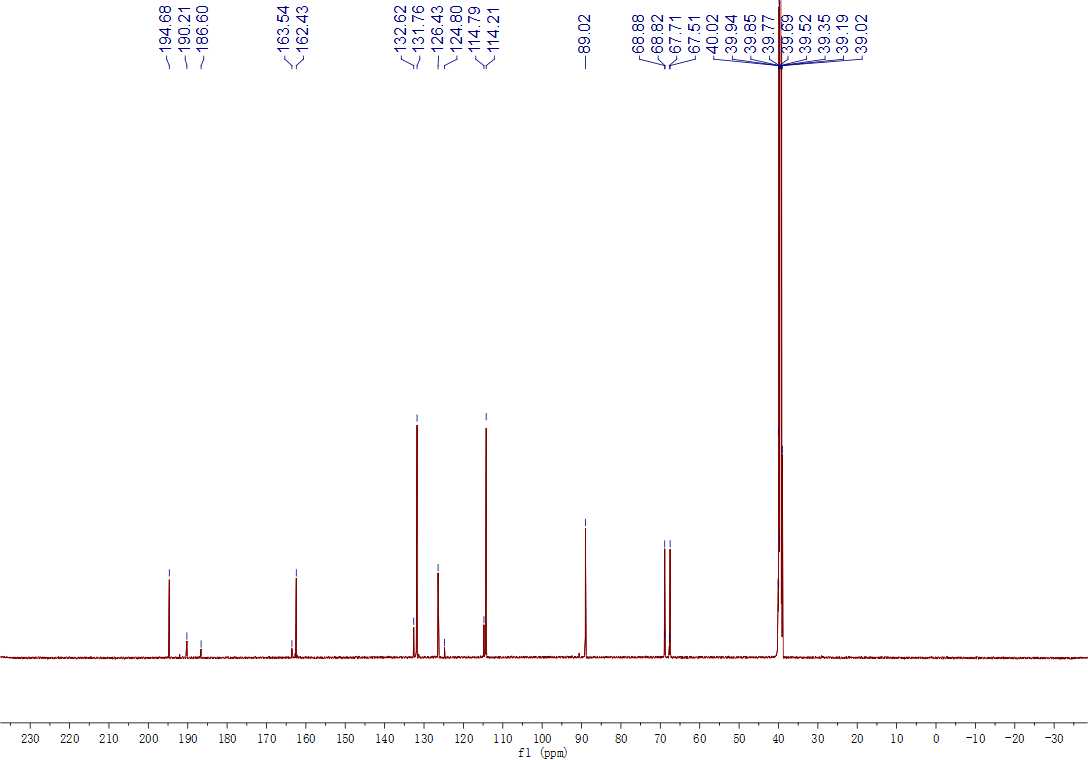


Supplementary Figure 57. **^13^C NMR spectrum of ArGO2 (100 MHz, (CD_3_)_2_SO)**


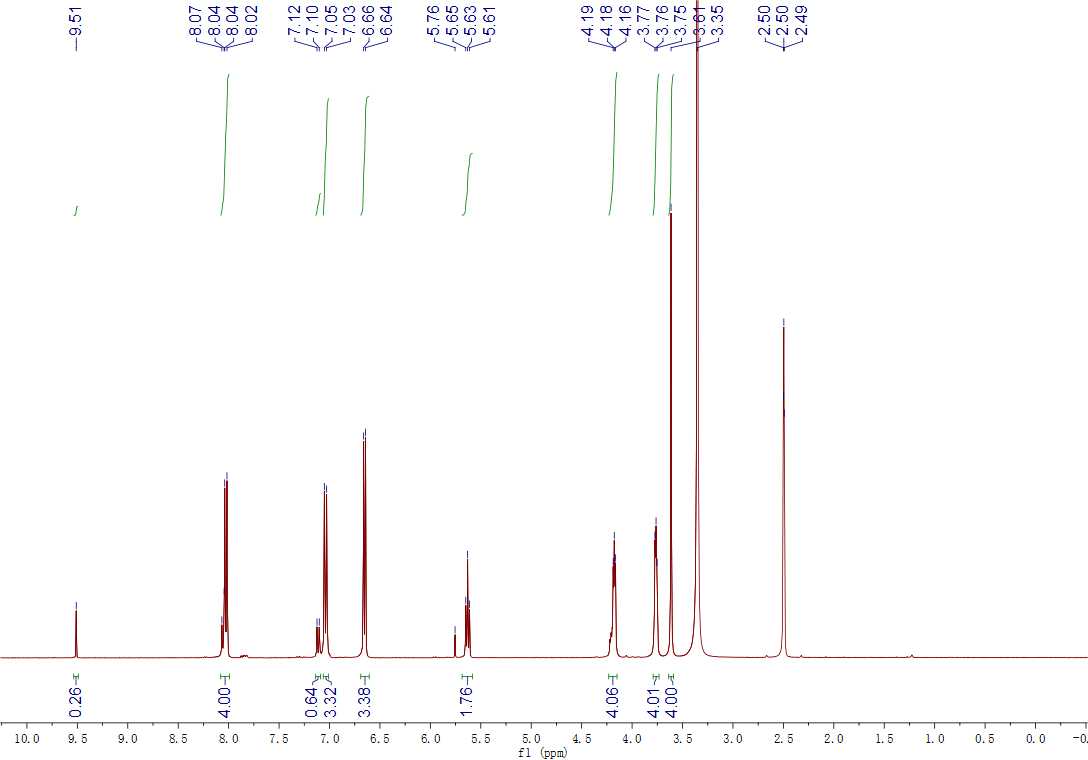


Supplementary Figure 58. **^1^H NMR spectrum of ArGO3(400 MHz, (CD_3_)_2_SO)**


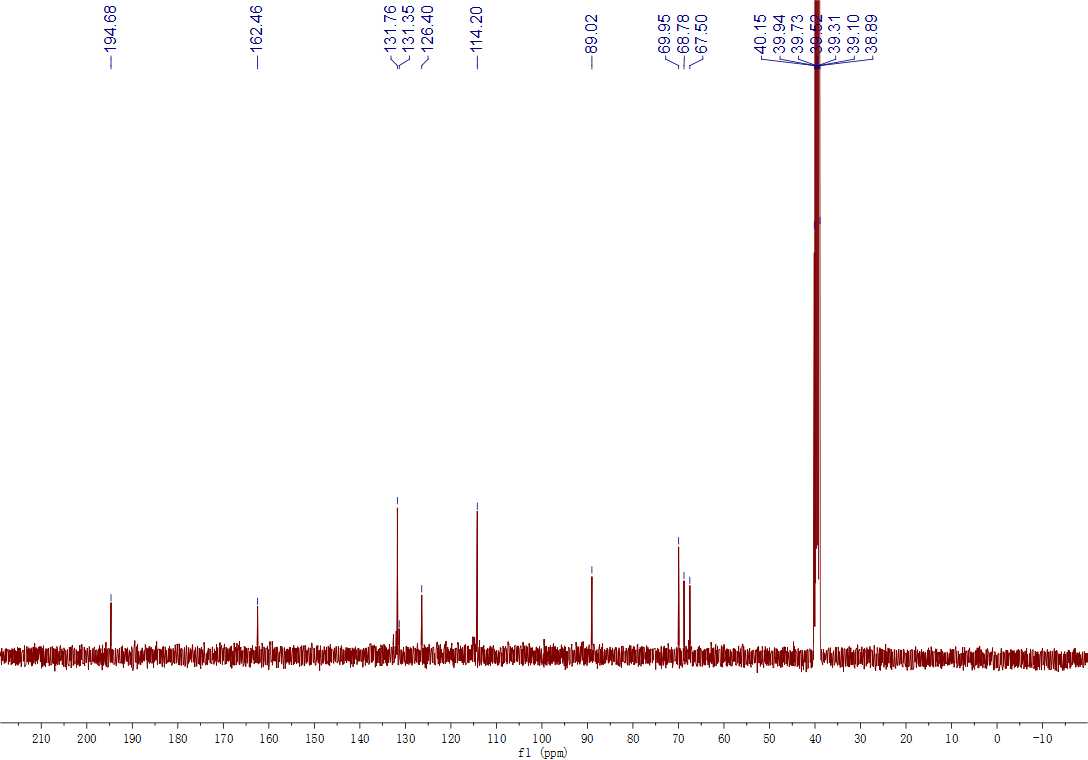


Supplementary Figure 59. **^13^C NMR spectrum of ArGO3 (100 MHz, (CD_3_)_2_SO)**


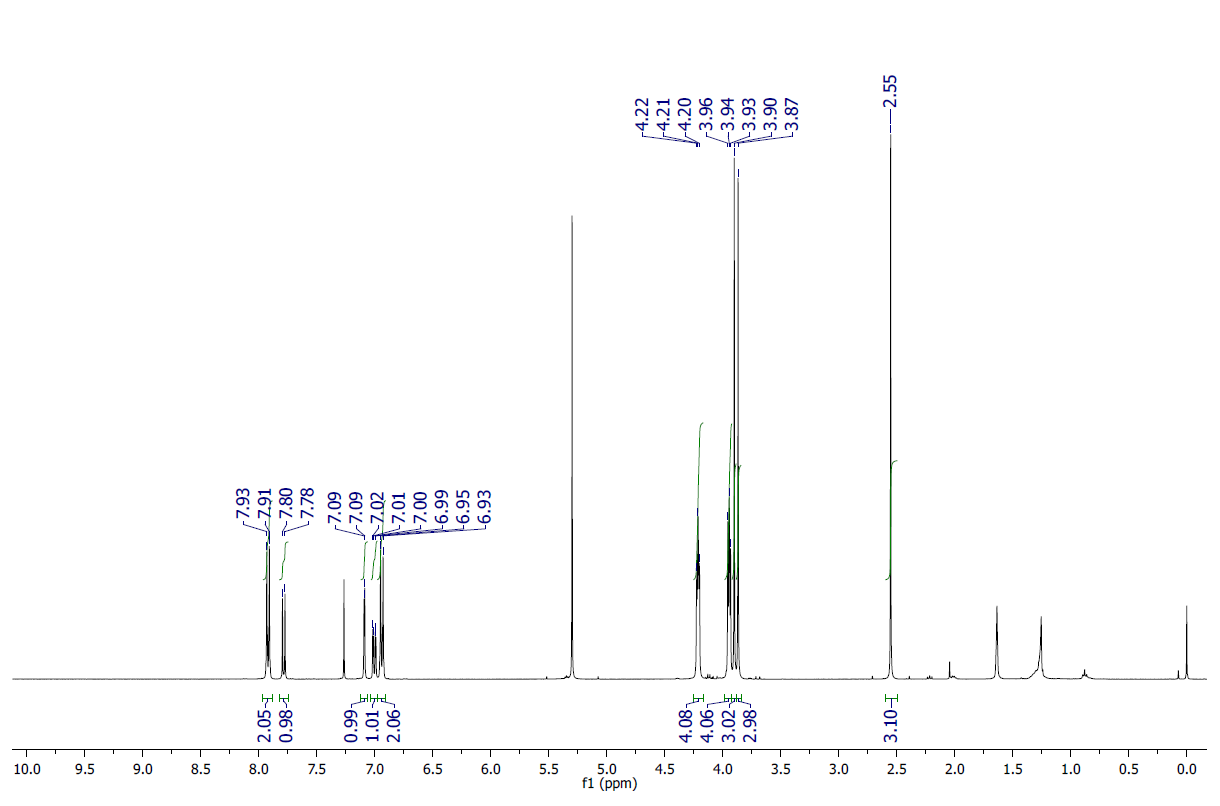


Supplementary Figure 60. **^1^H NMR spectrum of S2 (400 MHz, CDCl_3_)**


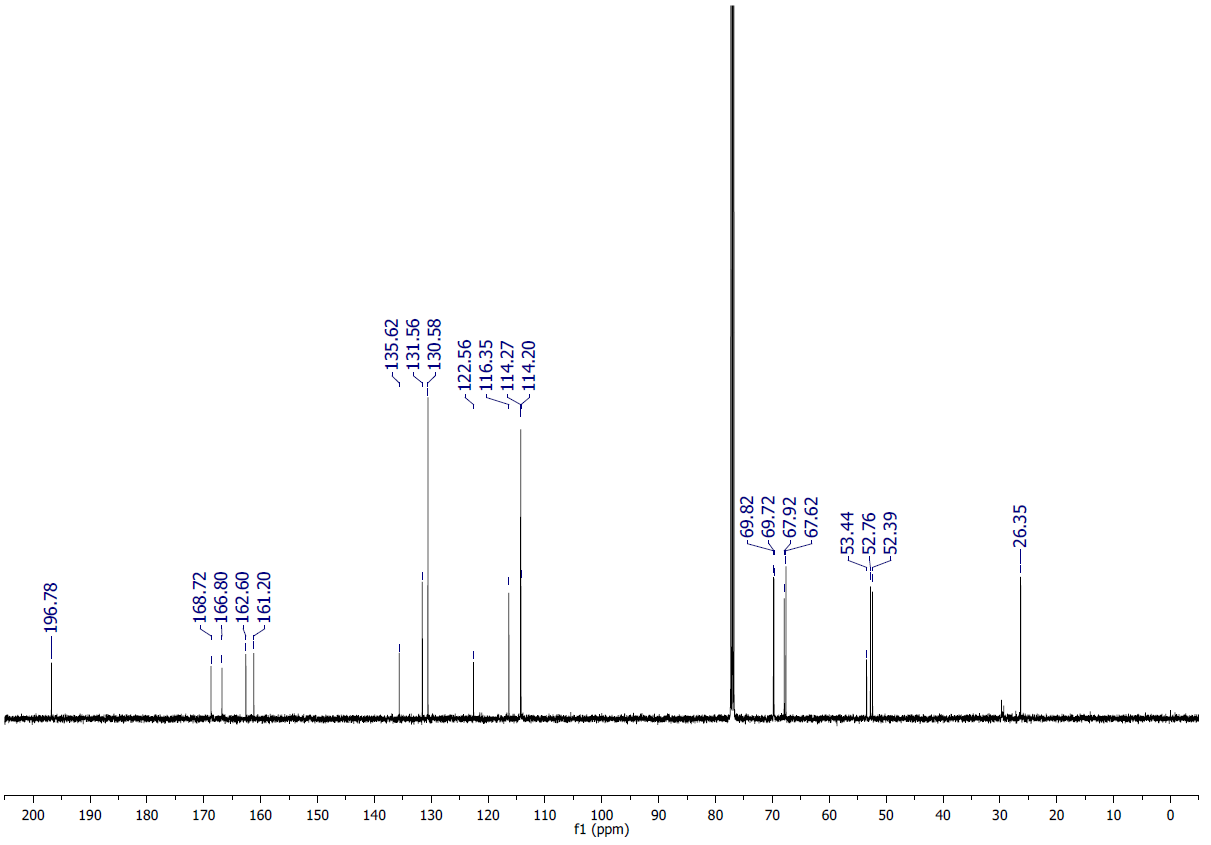


Supplementary Figure 61. **^13^C NMR spectrum of S2 (100 MHz, CDCl_3_)**

Supplementary Figure 62. **^1^H NMR spectrum of S3 (400 MHz, CDCl_3_)**


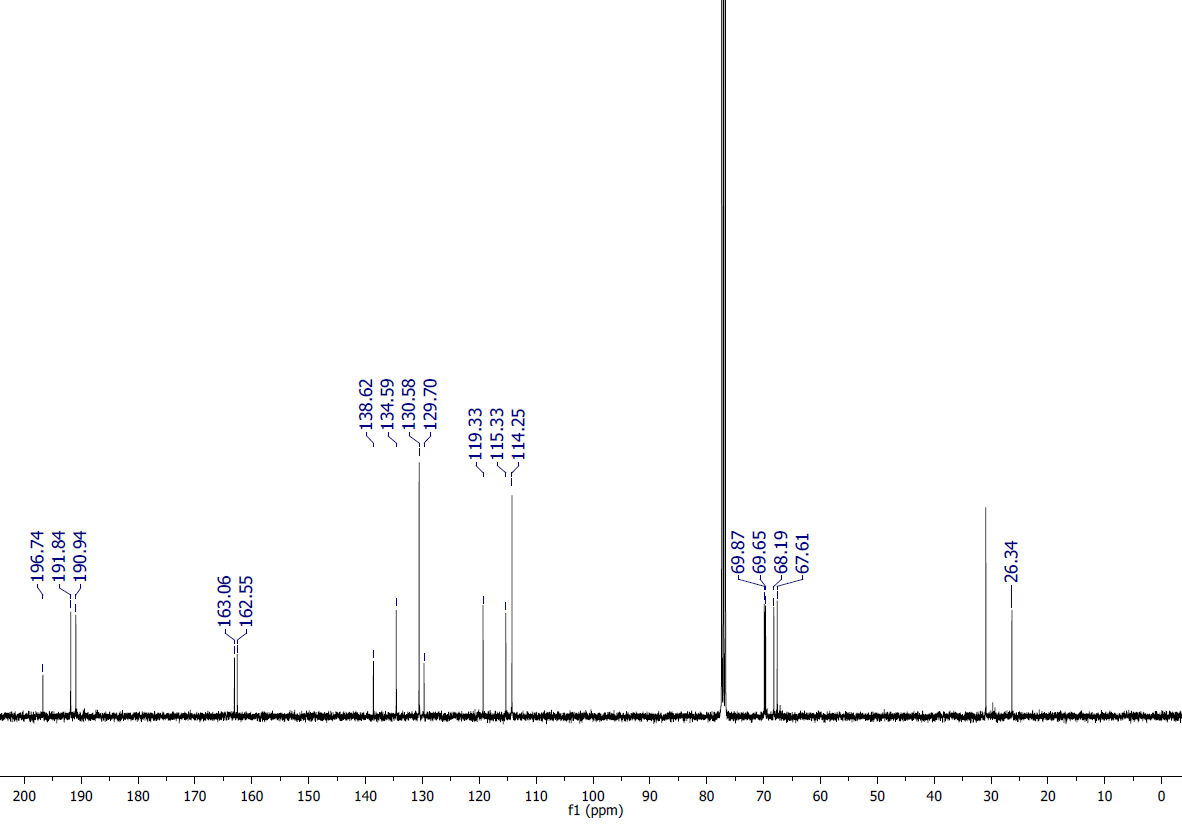
Supplementary Figure 63. **^13^C NMR spectrum of S3 (100 MHz, CDCl­_3_)**

Supplementary Figure 64. **^1^H NMR spectrum of KArGO (400 MHz, MeCN-d_3_)**


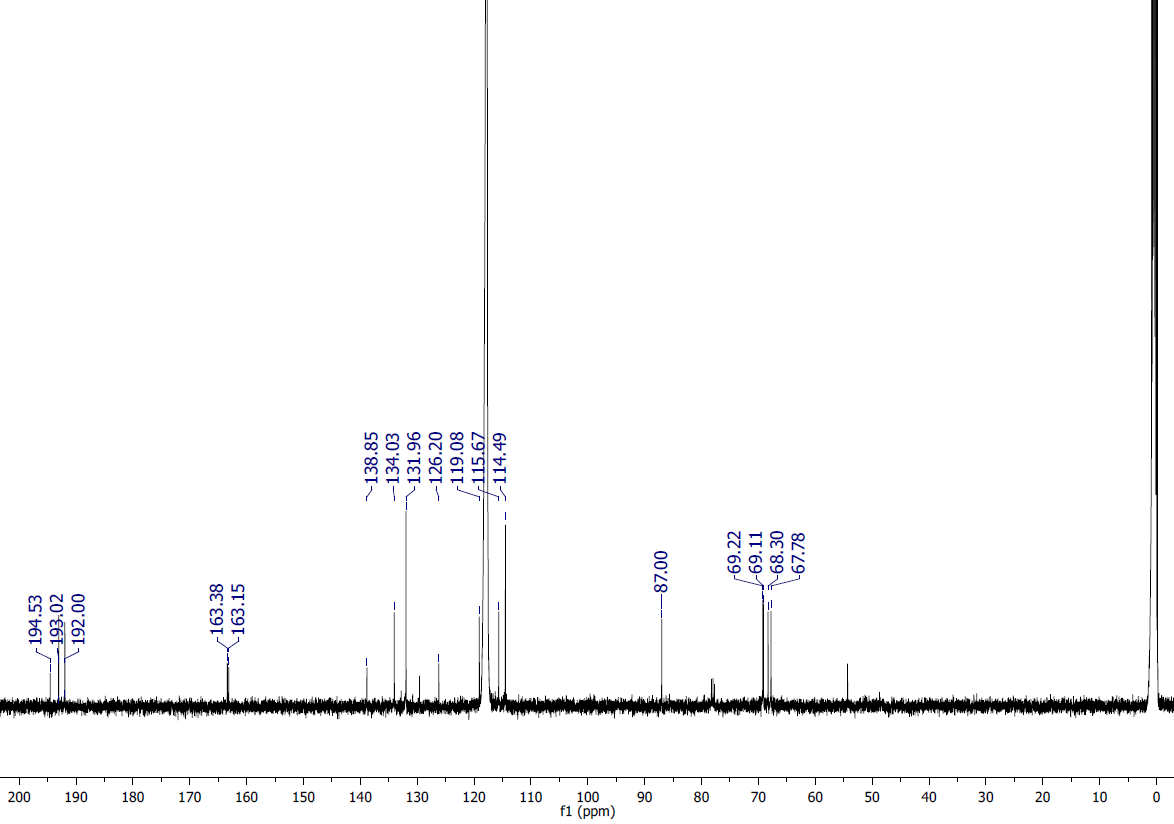


Supplementary Figure 65. **^13^C NMR spectrum of KArGO (400 MHz, MeCN-d_3_)**


Supplementary Figure 66. **^1^H NMR spectrum Adduct 3 (400 MHz, MeOH-d_4_)**

Supplementary Figure 67. **^13^C NMR spectrum of adduct 3 (100 MHz, MeOH-d_4_)**


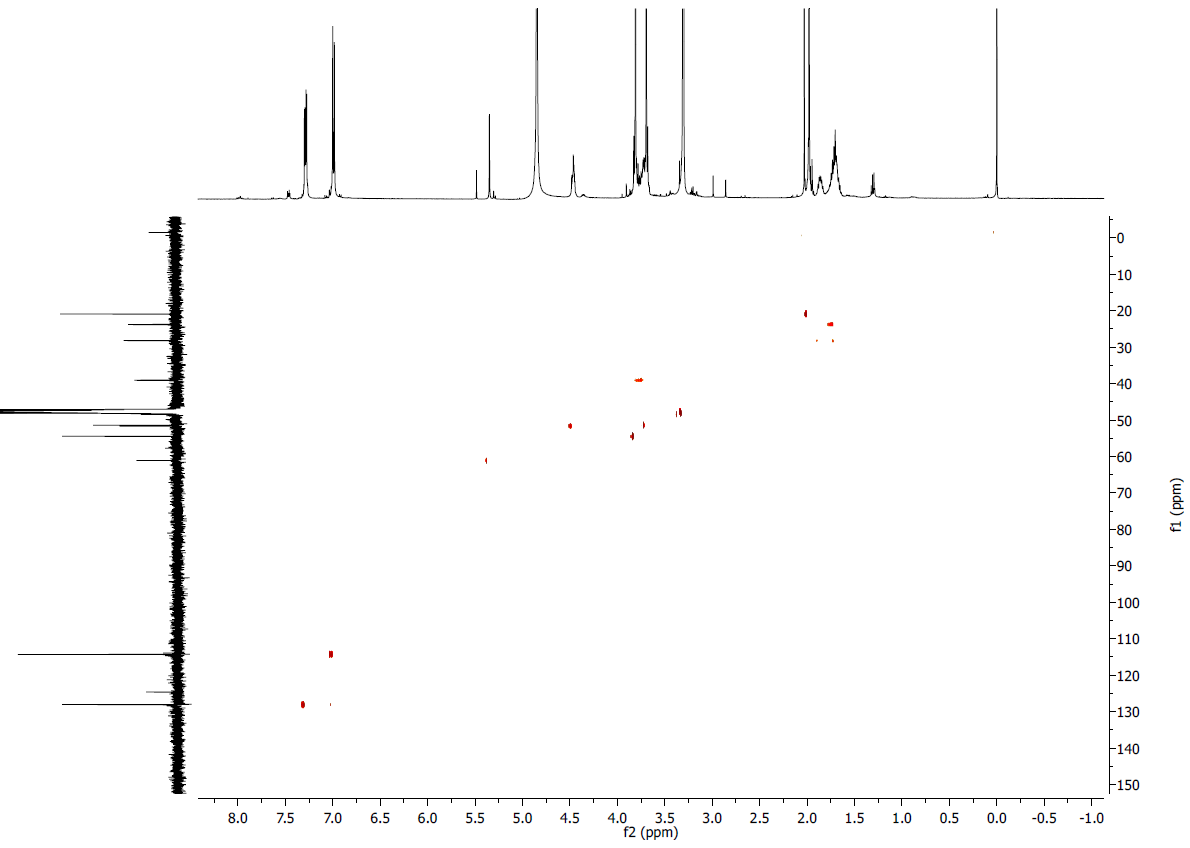
Supplementary Figure 68. **^1^H-^13^C HSQC NMR spectrum Adduct 3 (400 MHz, MeOH-d_4_)**


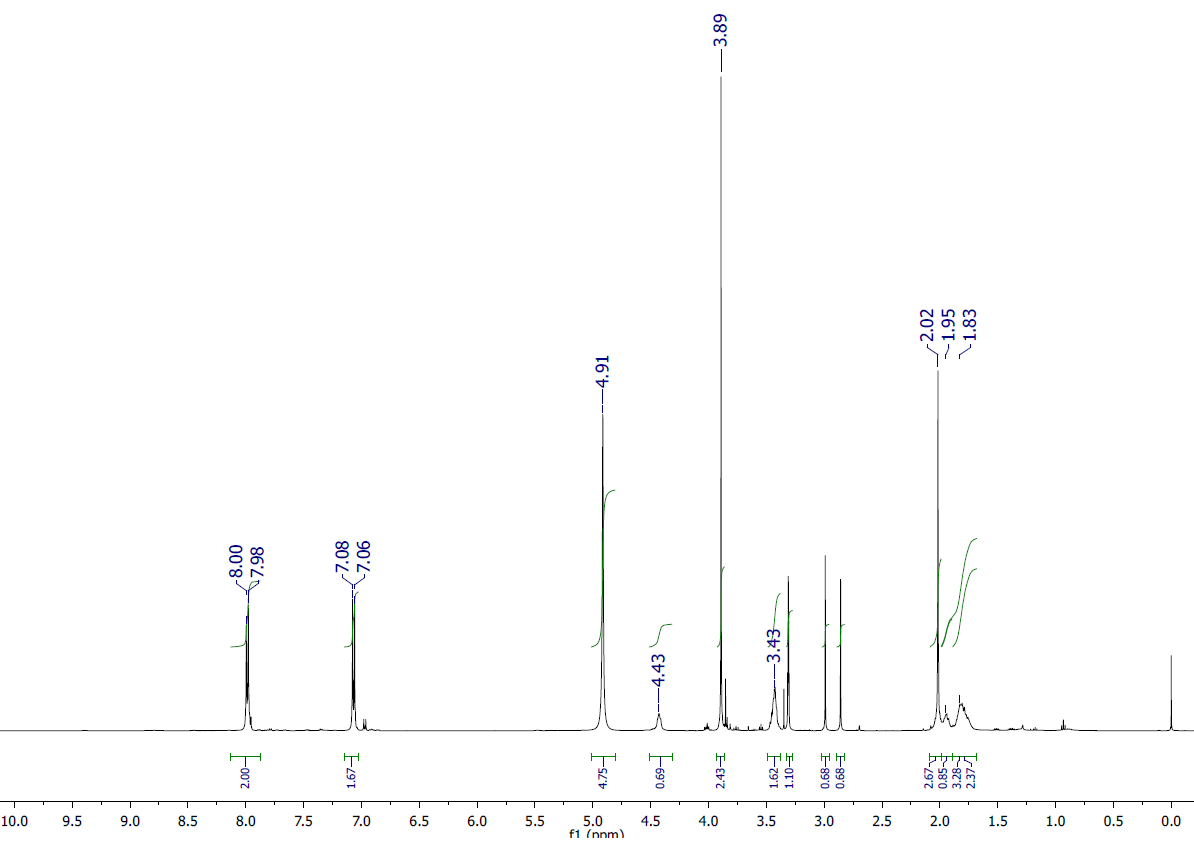


Supplementary Figure 69. **^1^H NMR spectrum Adduct 4 (500 MHz, MeOH-d_4_)**

Supplementary Figure 70. **^13^C NMR spectrum Adduct 4 (125 MHz, MeOH-d_4_)**


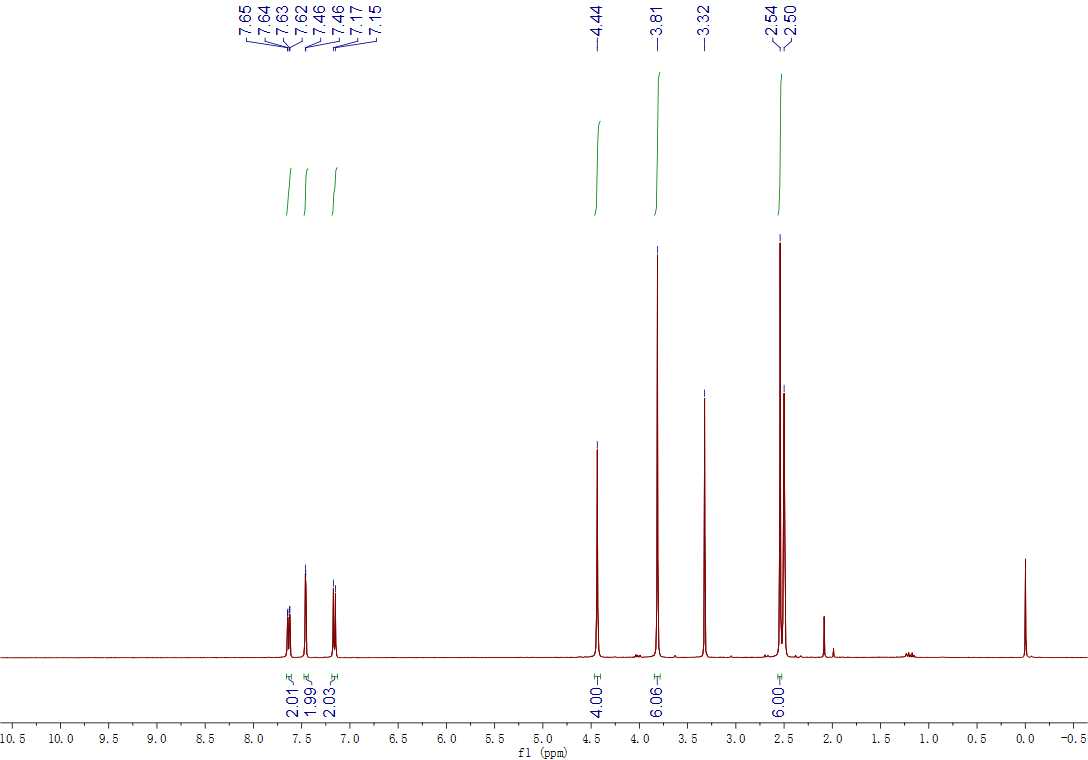


Supplementary Figure 71. **^1^H NMR spectrum of pre-OMe-ArGO-1 (400 MHz, (CD_3_)_2_SO)**


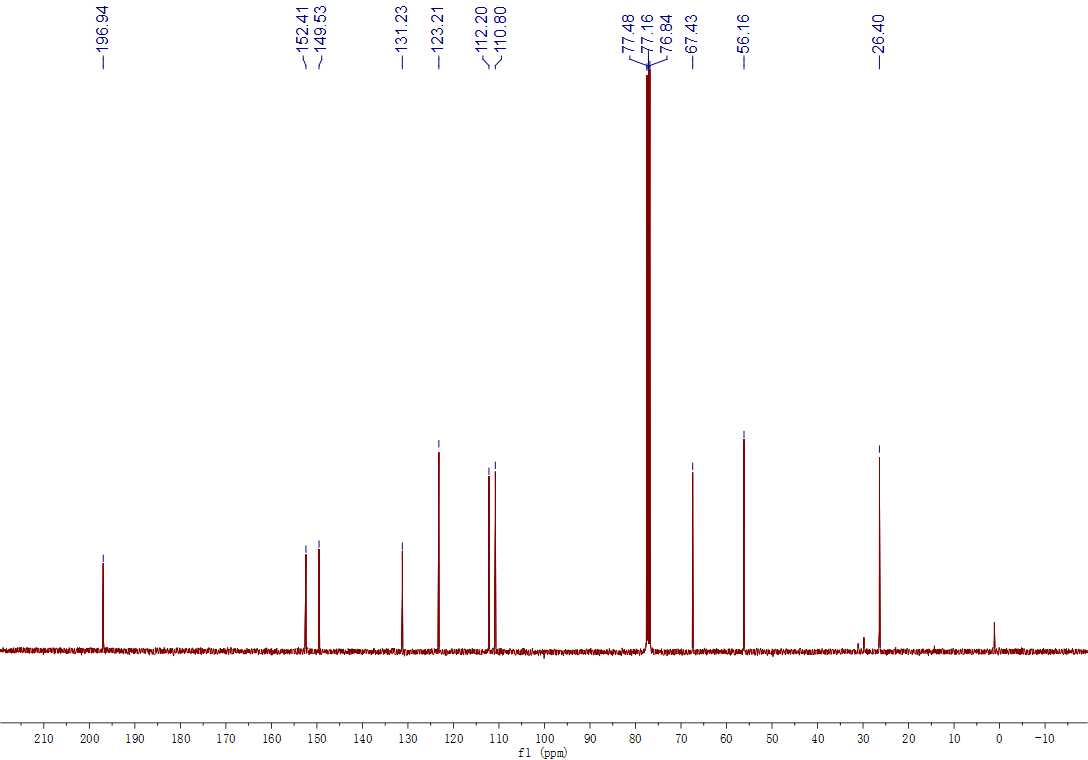


Supplementary Figure 72. **^13^C NMR spectrum of pre-OMe-ArGO-1 (100 MHz, CDCl_3_)**


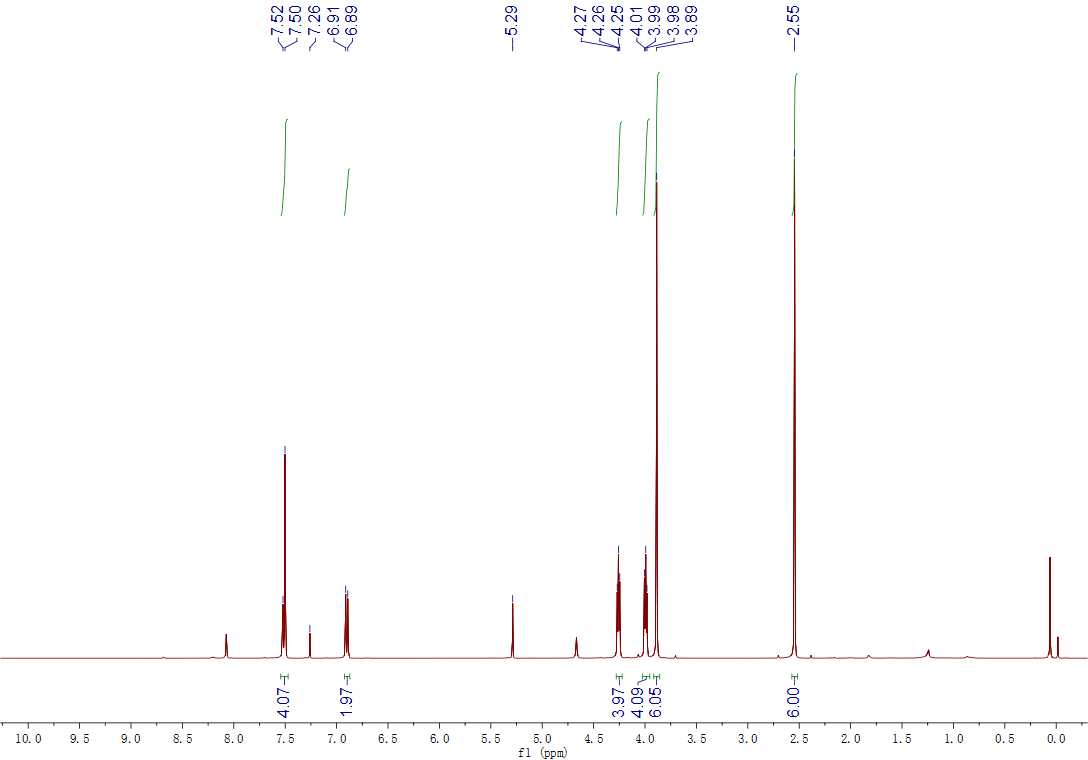


Supplementary Figure 73. **^1^H NMR spectrum of pre-OMe-ArGO2 (400 MHz, CDCl_3_)**


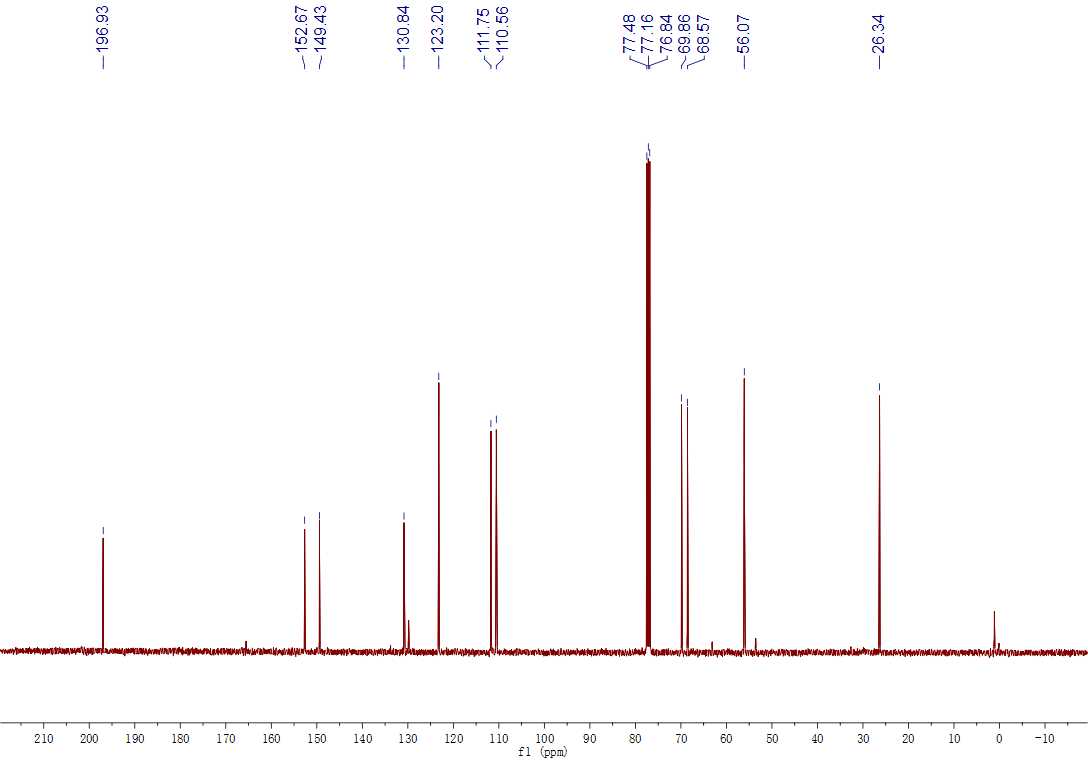
Supplementary Figure 74. **^13^C NMR spectrum of pre-OMe-ArGO2 (100 MHz, CDCl_3_)**


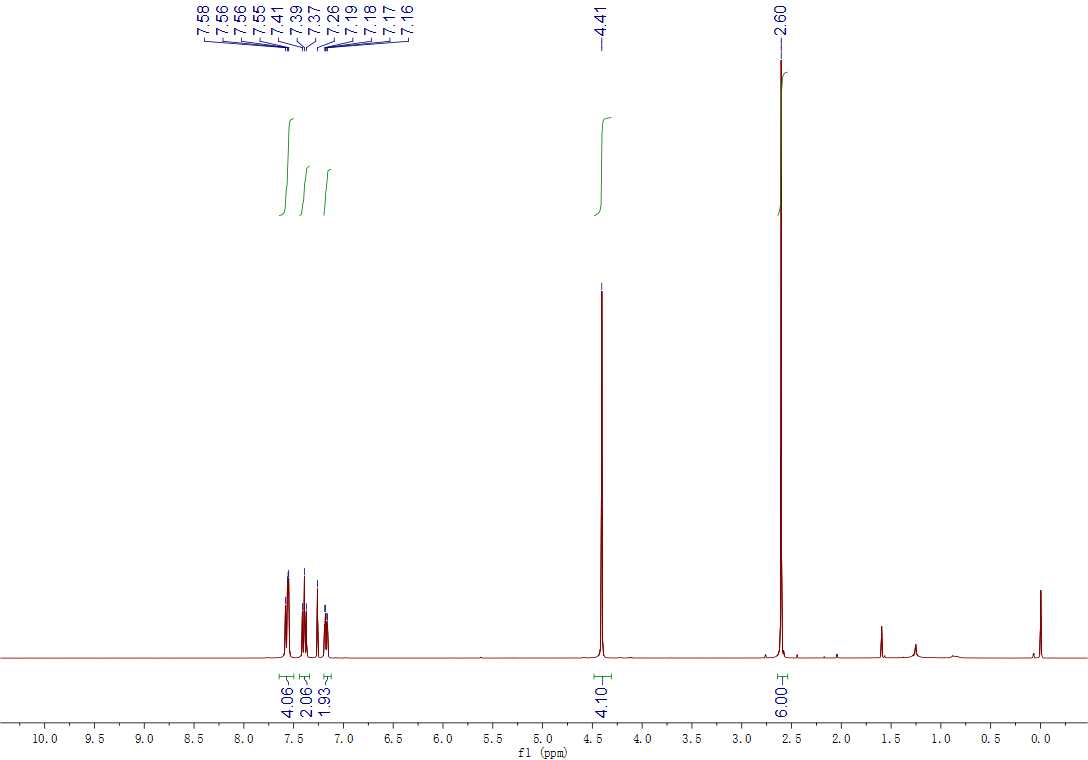


Supplementary Figure 75. **^1^H NMR spectrum of pre-*meta*-ArGO1 (400 MHz, CDCl_3_)**


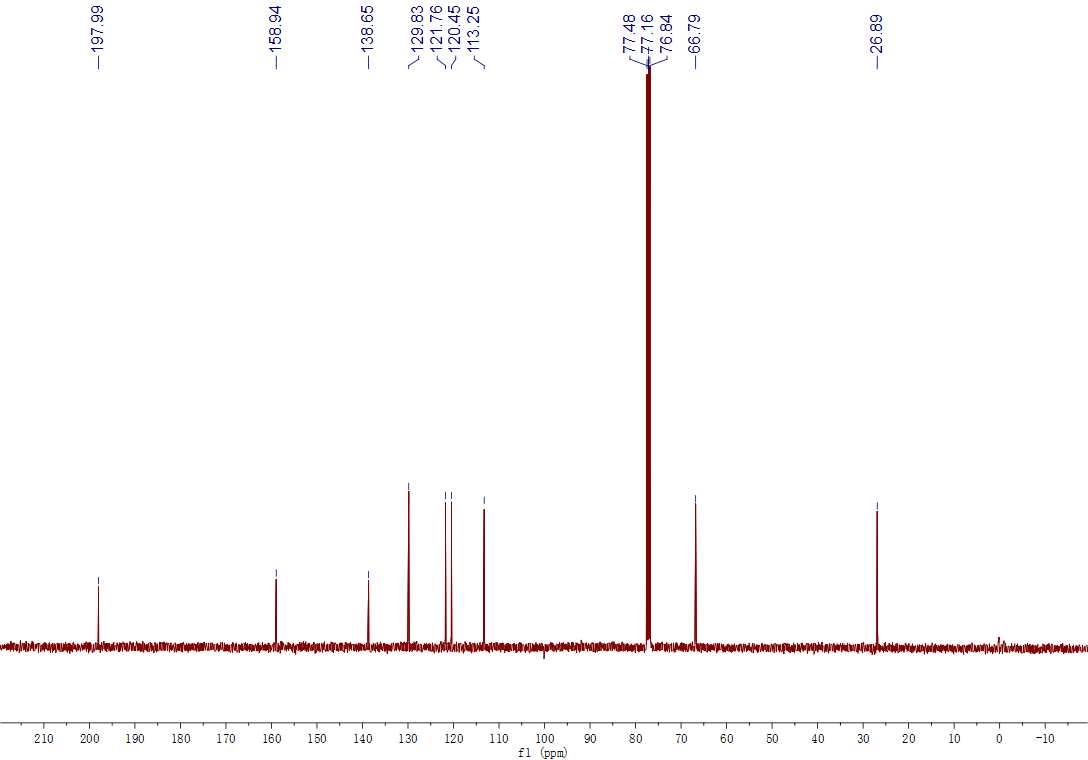


Supplementary Figure 76. **^13^C NMR spectrum of pre-*meta*-ArGO1 (100 MHz, CDCl_3_)**


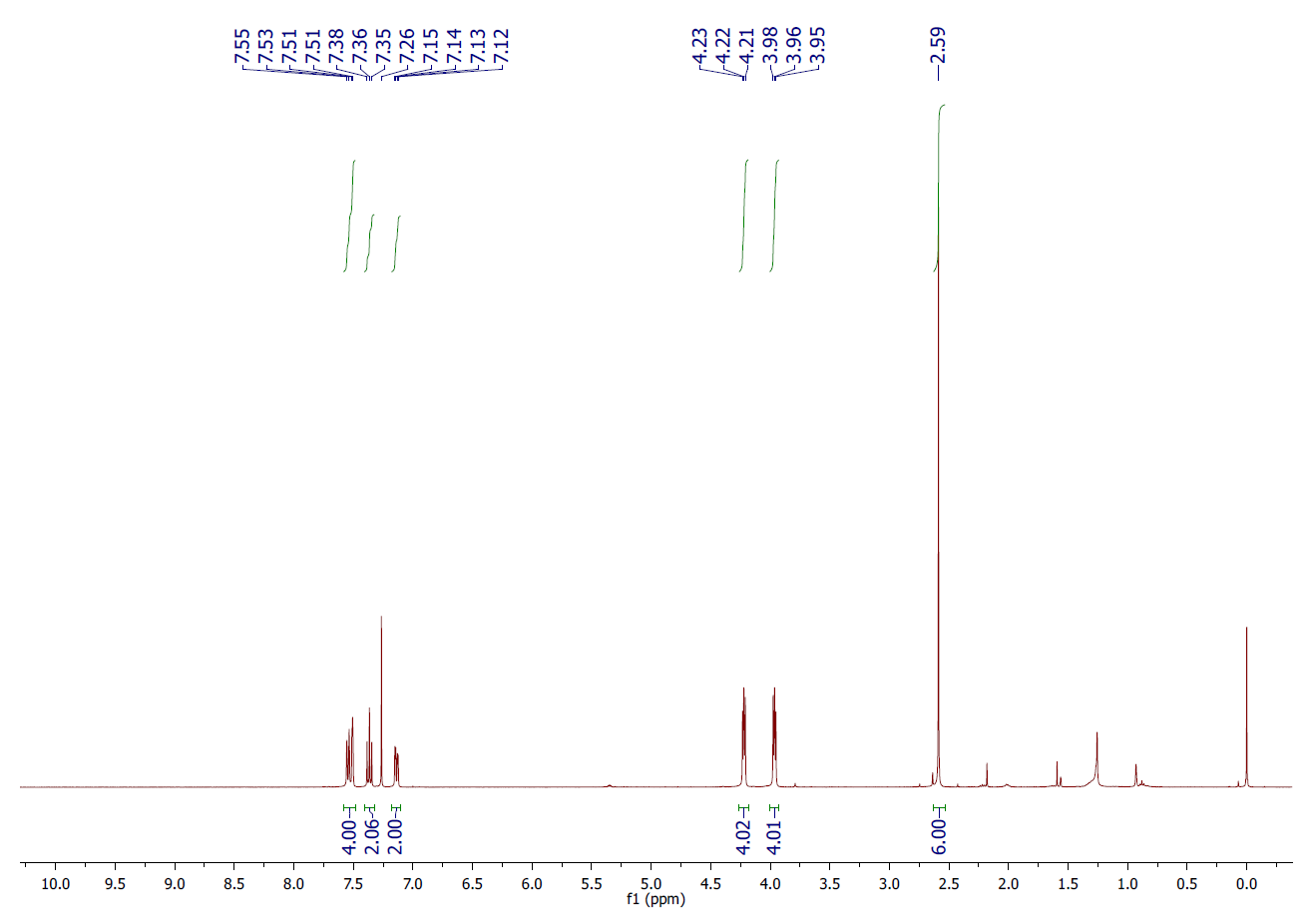


Supplementary Figure 77. **^1^H NMR spectrum of pre-*meta*-ArGO2 (400 MHz, CDCl_3_)**


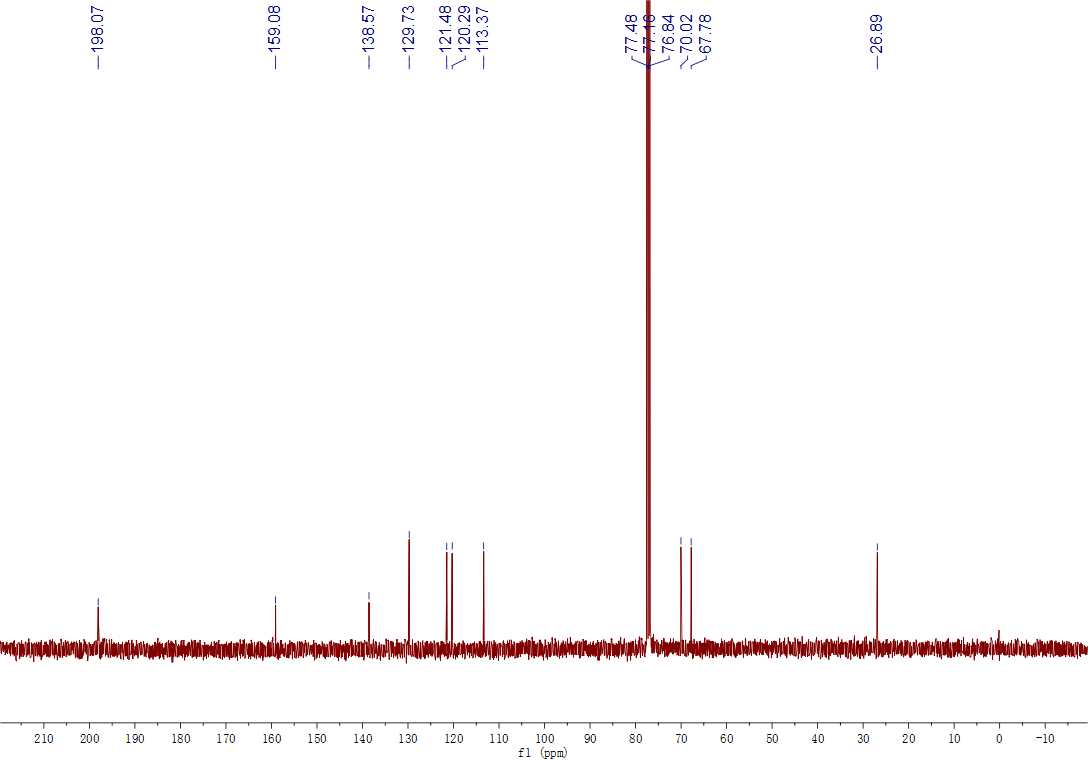


Supplementary Figure 78. **^13^C NMR spectrum of pre-*meta*-ArGO2 (100 MHz, CDCl_3_)**


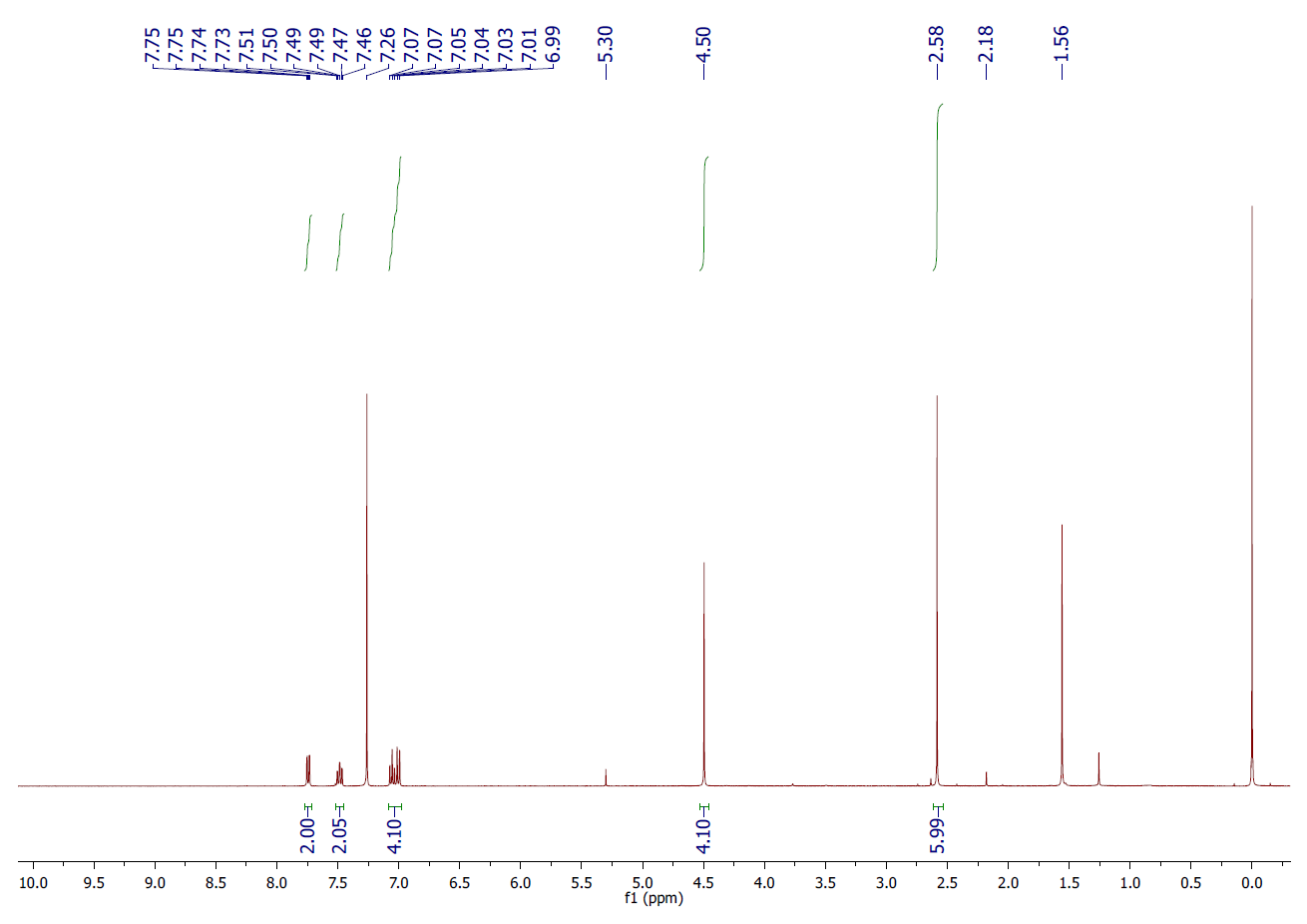


Supplementary Figure 79. **^1^H NMR spectrum of pre-*ortho*-ArGO-1 (400 MHz, CDCl_3_)**


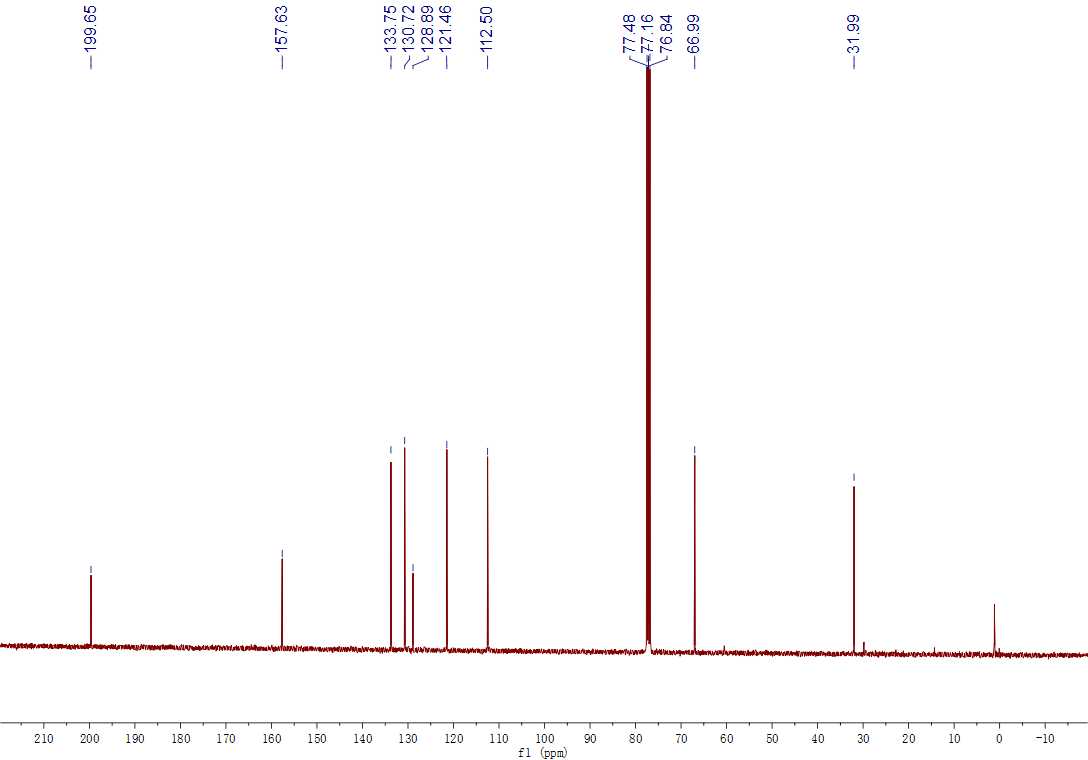


Supplementary Figure 80. **^13^C NMR spectrum of pre-*ortho*-ArGO1 (100 MHz, CDCl_3_)**


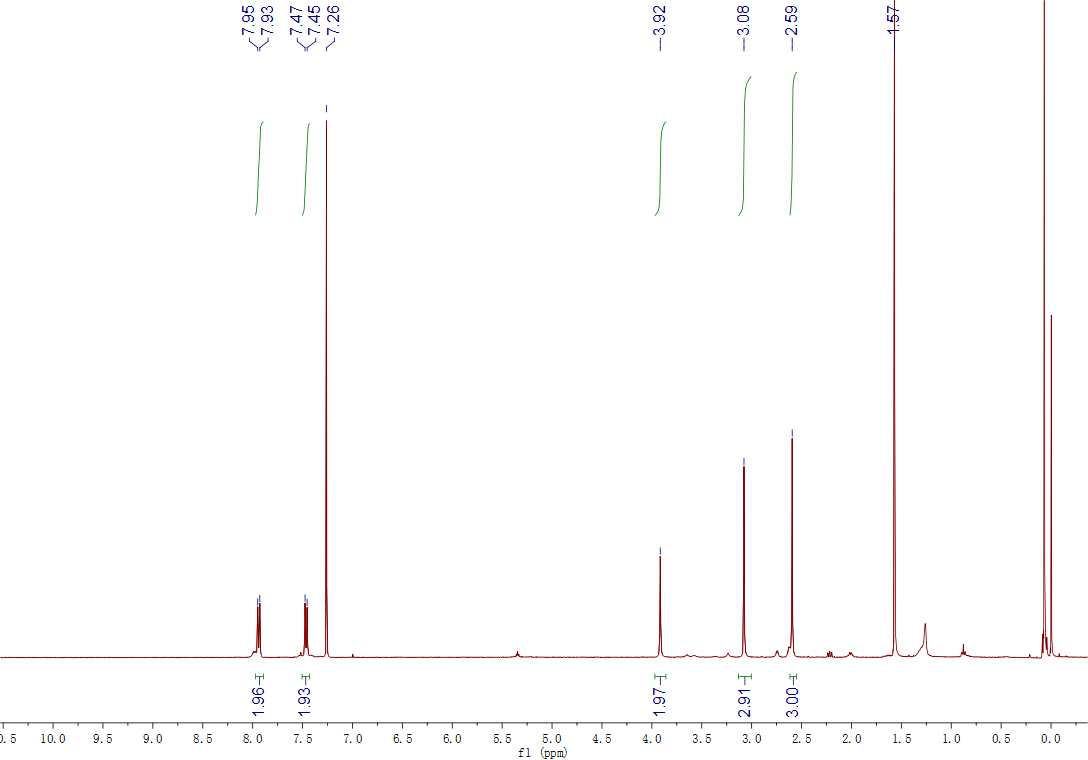


Supplementary Figure 81. **^1^H NMR spectrum of pre-N-Me-amide-ArGO1 (400 MHz, CDCl_3_)**


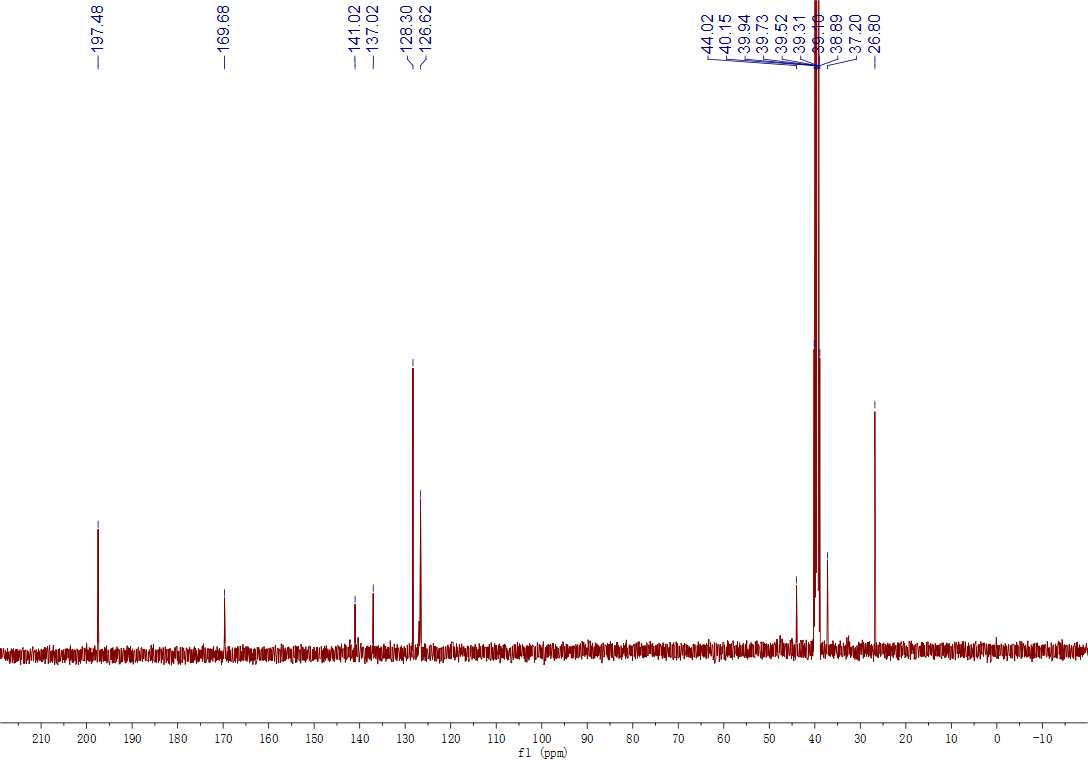
Supplementary Figure 82. **^13^C NMR spectrum of pre-N-Me-amide-ArGO1 (100 MHz, (CD_3_)_2_SO)**


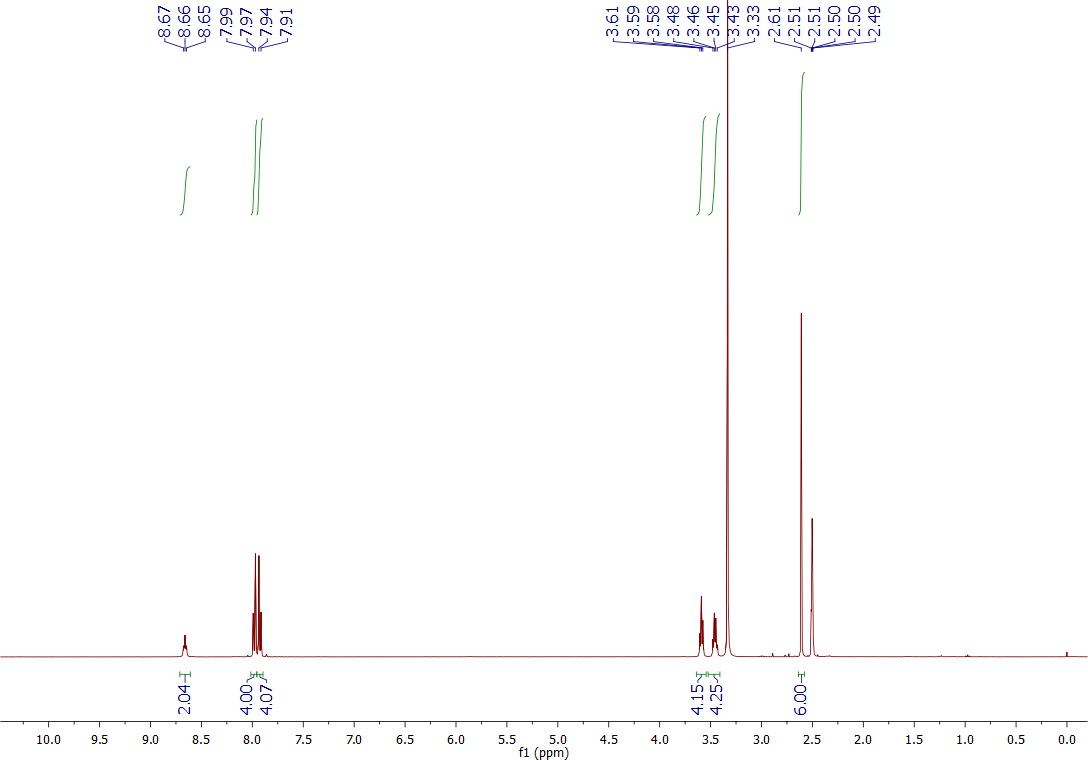


Supplementary Figure 83. **^1^H NMR spectrum of pre-Amide-ArGO2 (400 MHz, CDCl_3_)**


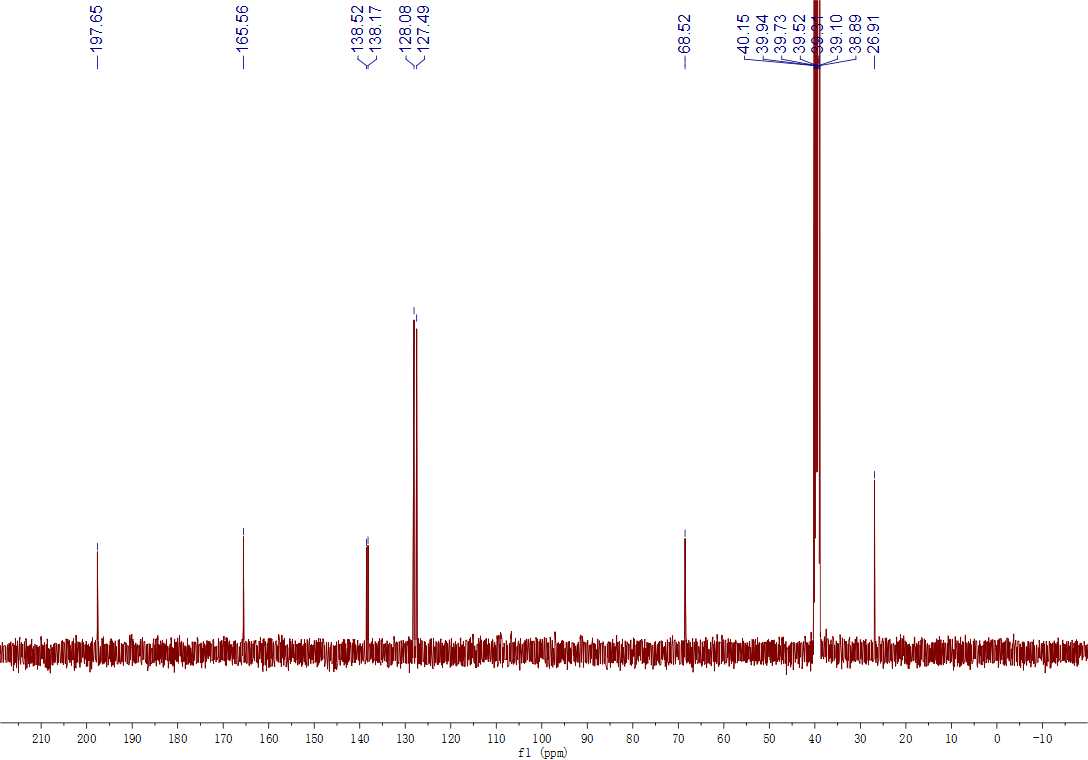


Supplementary Figure 84. **^13^C NMR spectrum of pre-Amide-ArGO2 (100 MHz, CDCl_3_)**


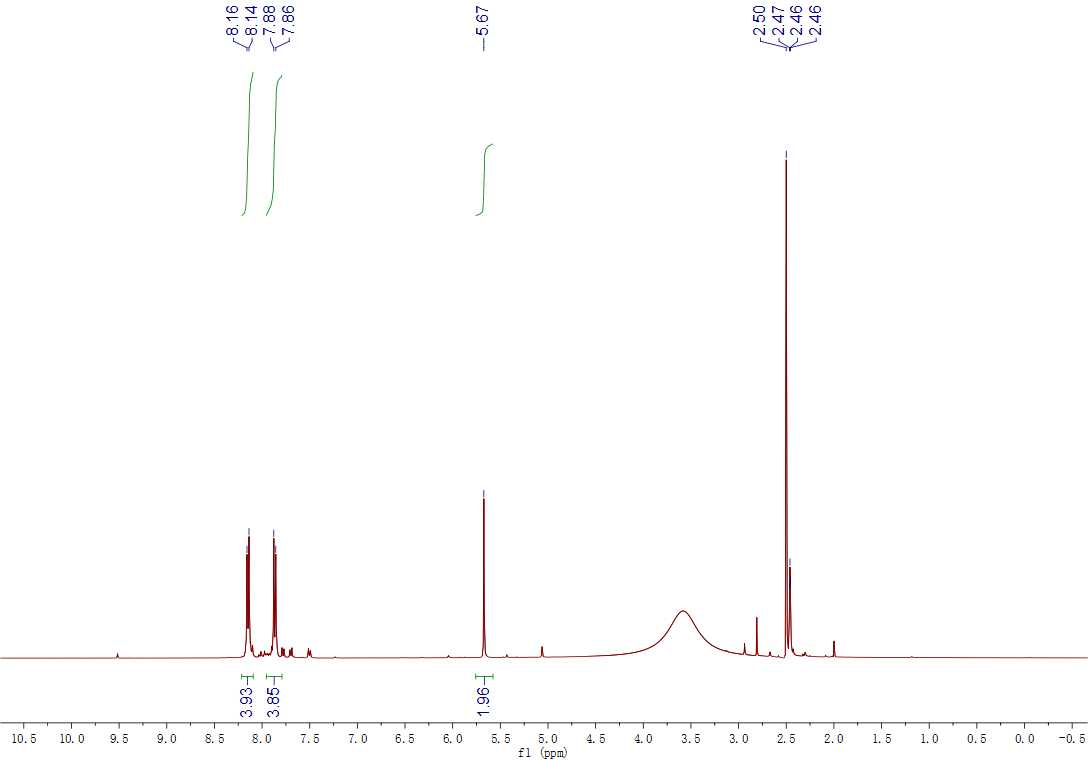


Supplementary Figure 85 **^1^H NMR spectrum of BDG (400 MHz, (CD_3_)_2_SO)**


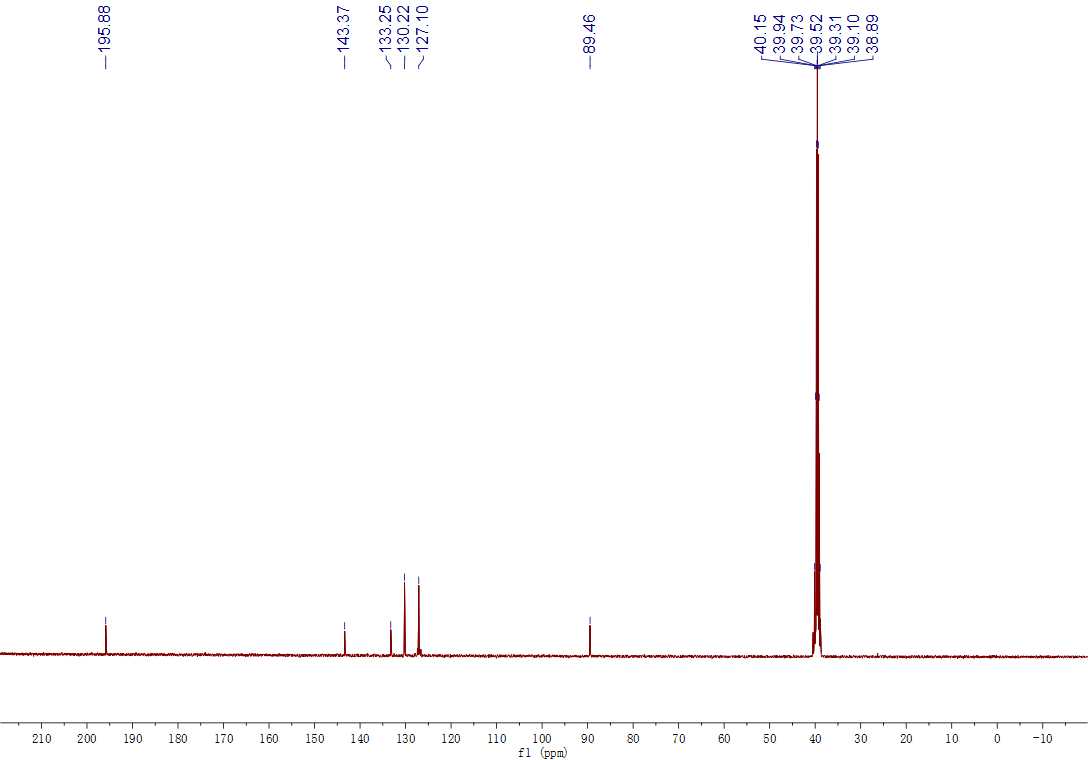
Supplementary Figure 86. **^13^C NMR spectrum of BDG (100 MHz, (CD_3_)_2_SO)**


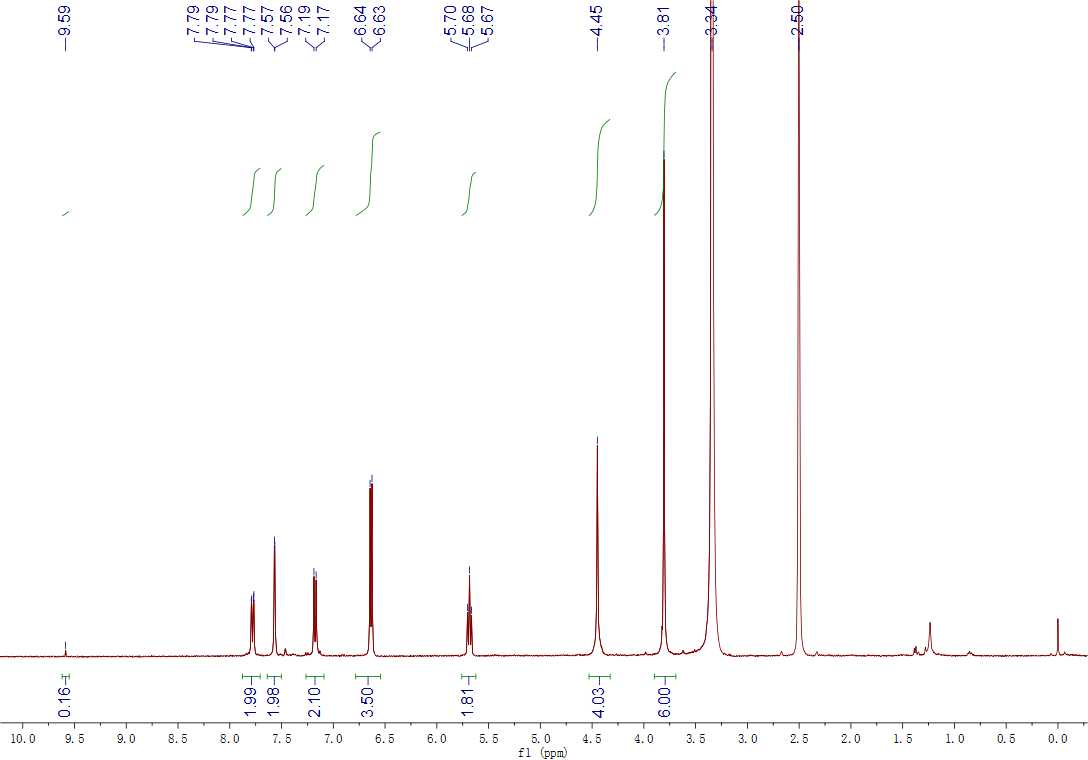


Supplementary Figure 87. **^1^H NMR spectrum of OMe-ArGO-1 (400 MHz, (CD_3_)_2_SO)**


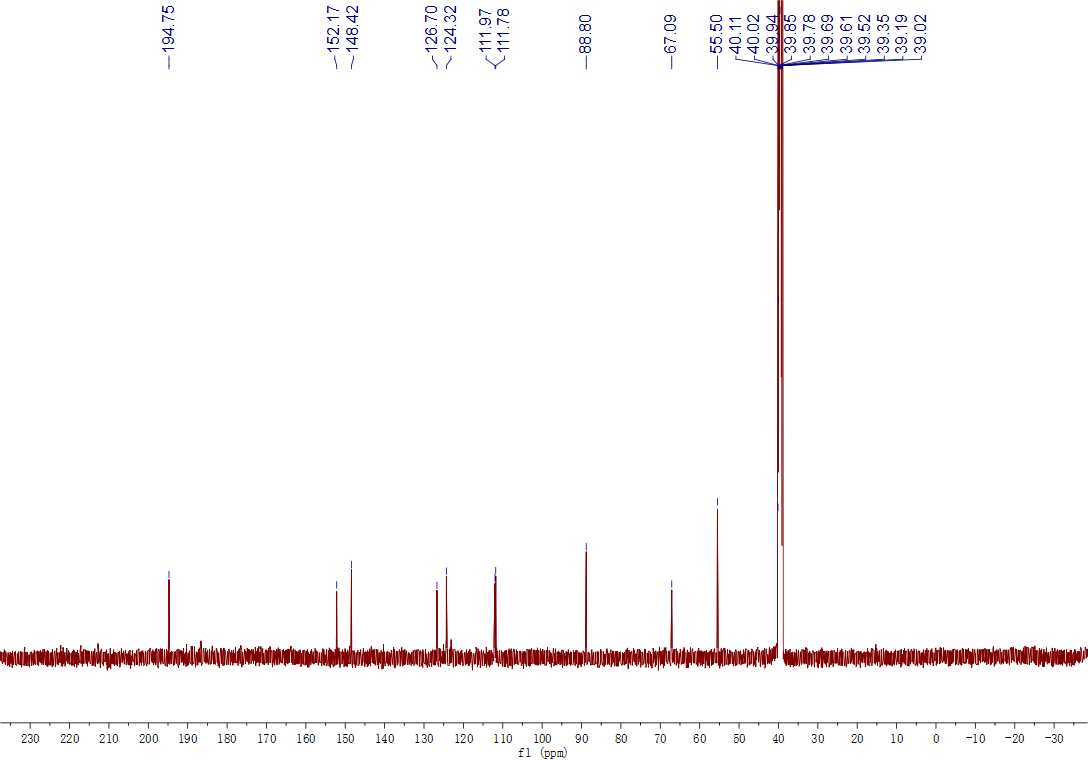


Supplementary Figure 88. **^13^C NMR spectrum of OMe-ArGO-1 (100 MHz, (CD_3_)_2_SO)**


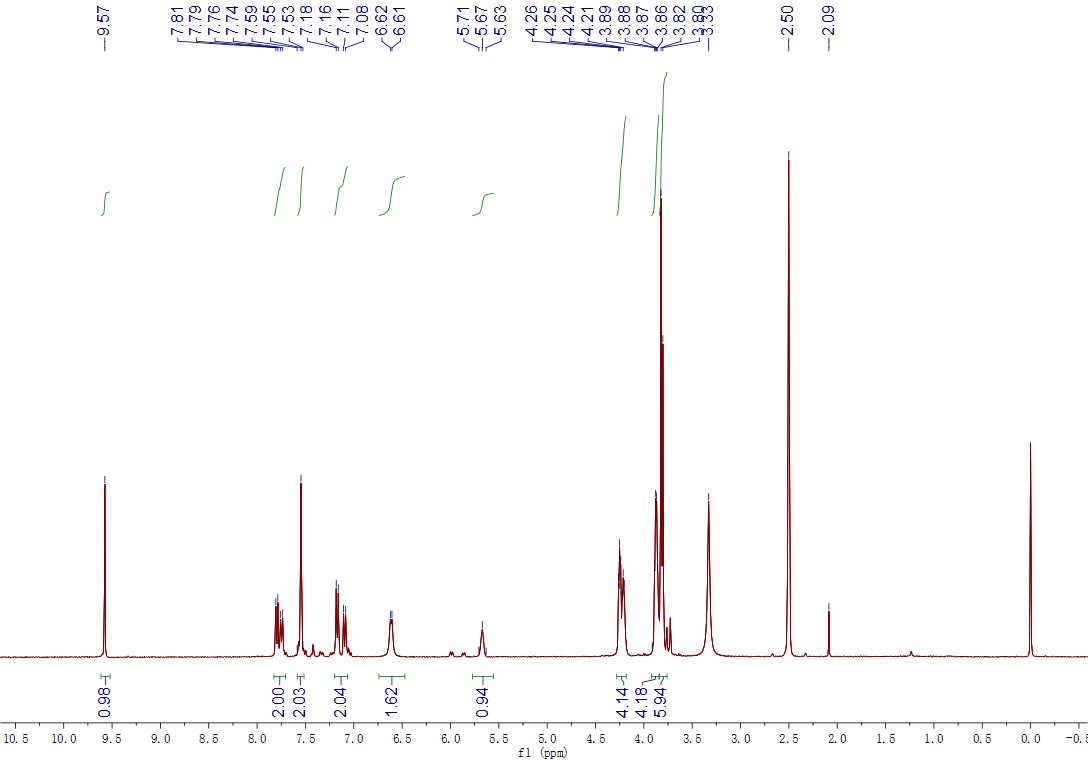


Supplementary Figure 89. **^1^H NMR spectrum of OMe-ArGO2 (400 MHz, (CD_3_)_2_SO)**


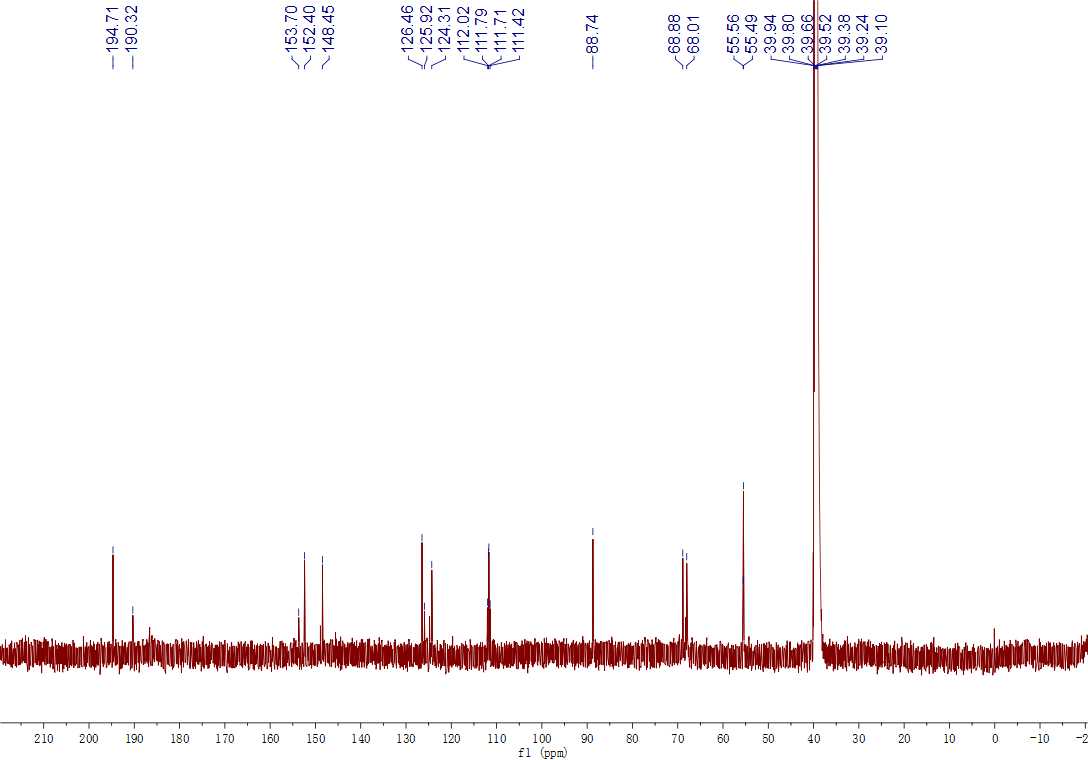


Supplementary Figure 90. **^13^C NMR spectrum of OMe-ArGO2 (100 MHz, (CD_3_)_2_SO)**


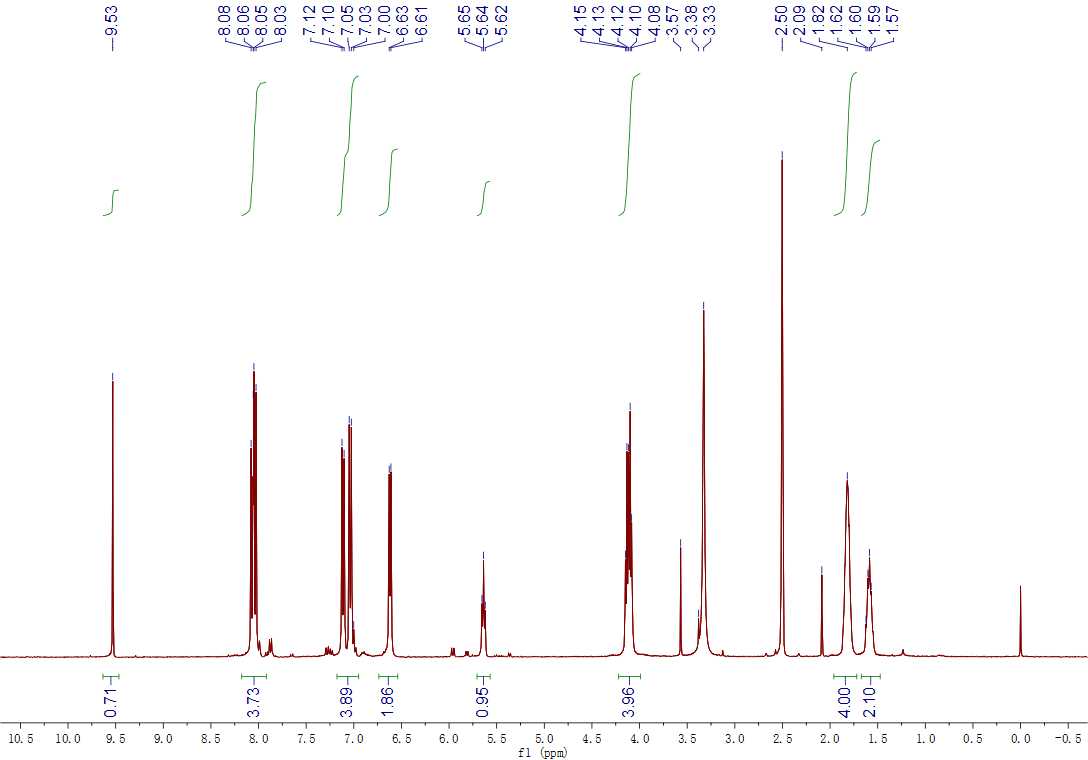


Supplementary Figure 91. **^1^H NMR spectrum of HP-ArGO (400 MHz, (CD_3_)_2_SO)**


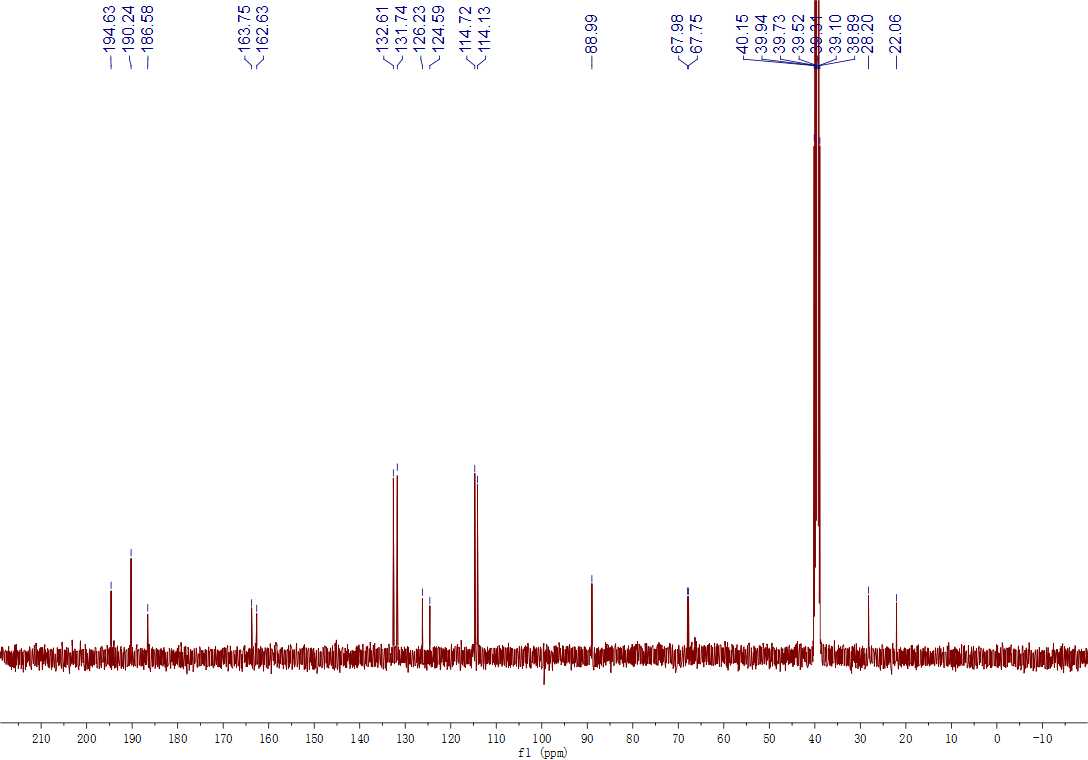


Supplementary Figure 92. **^13^C NMR spectrum of HP-ArGO (100 MHz, (CD_3_)_2_SO)**


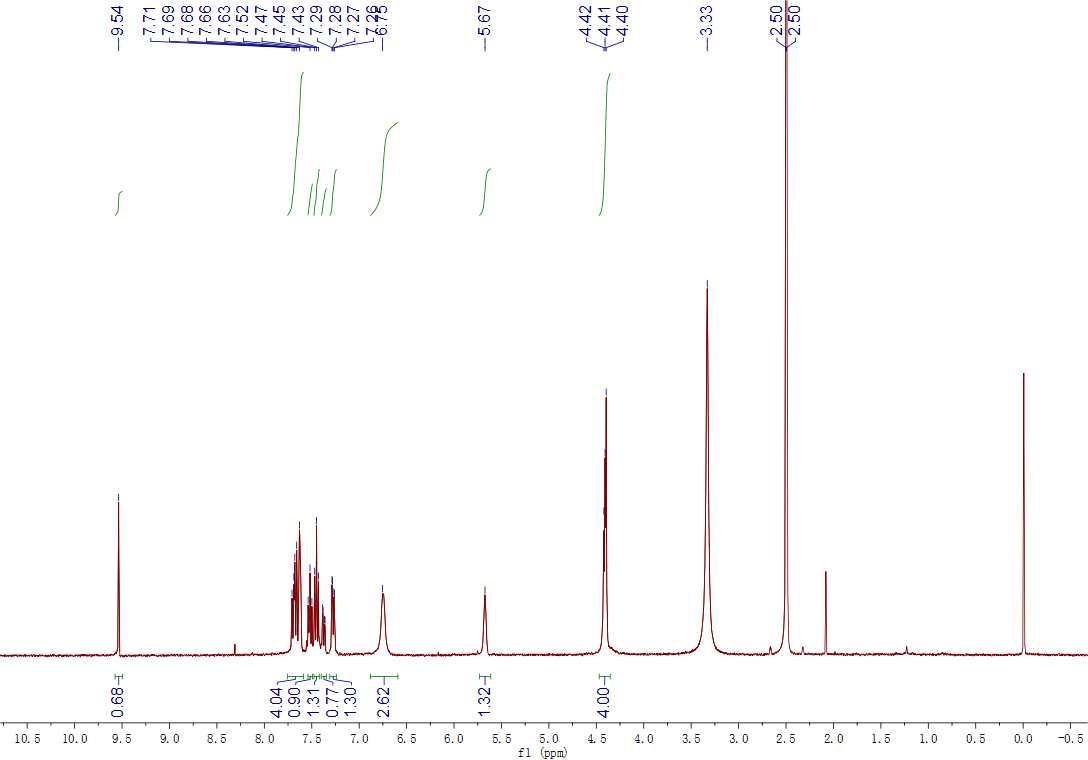


Supplementary Figure 93. **^1^H NMR spectrum of *meta*-ArGO1 (400 MHz, (CD_3_)_2_SO)**


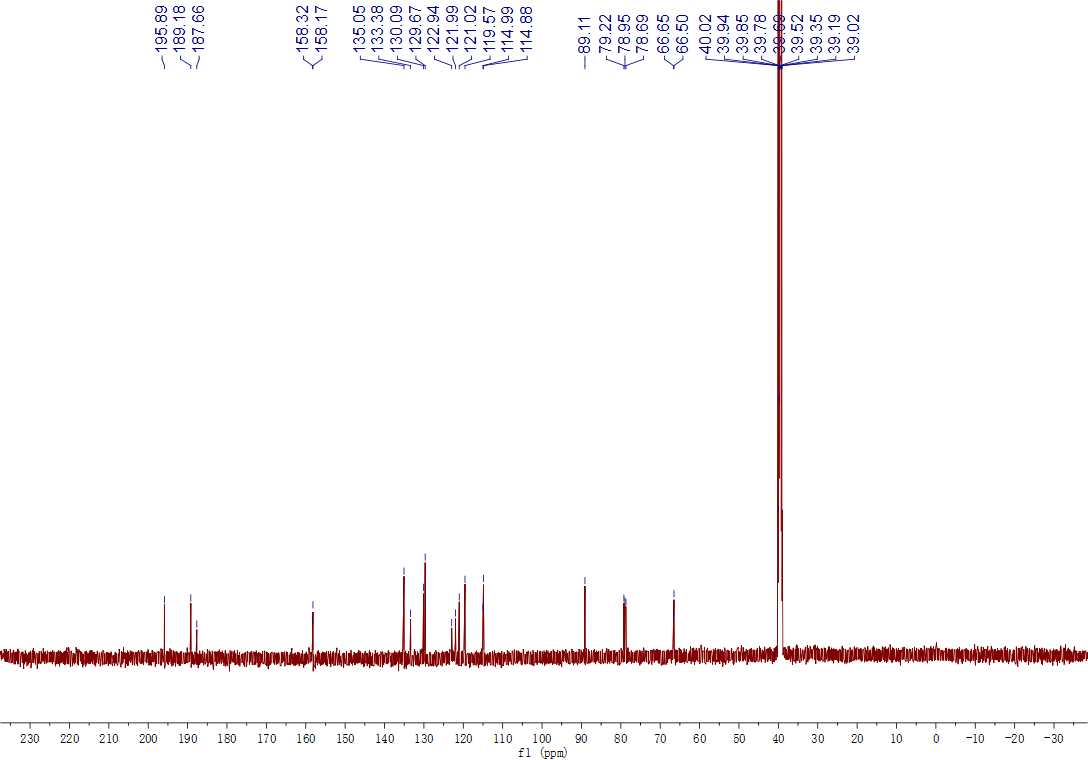


Supplementary Figure 94. **^13^C NMR spectrum of *meta*-ArGO-1 (100 MHz, (CD_3_)_2_SO)**


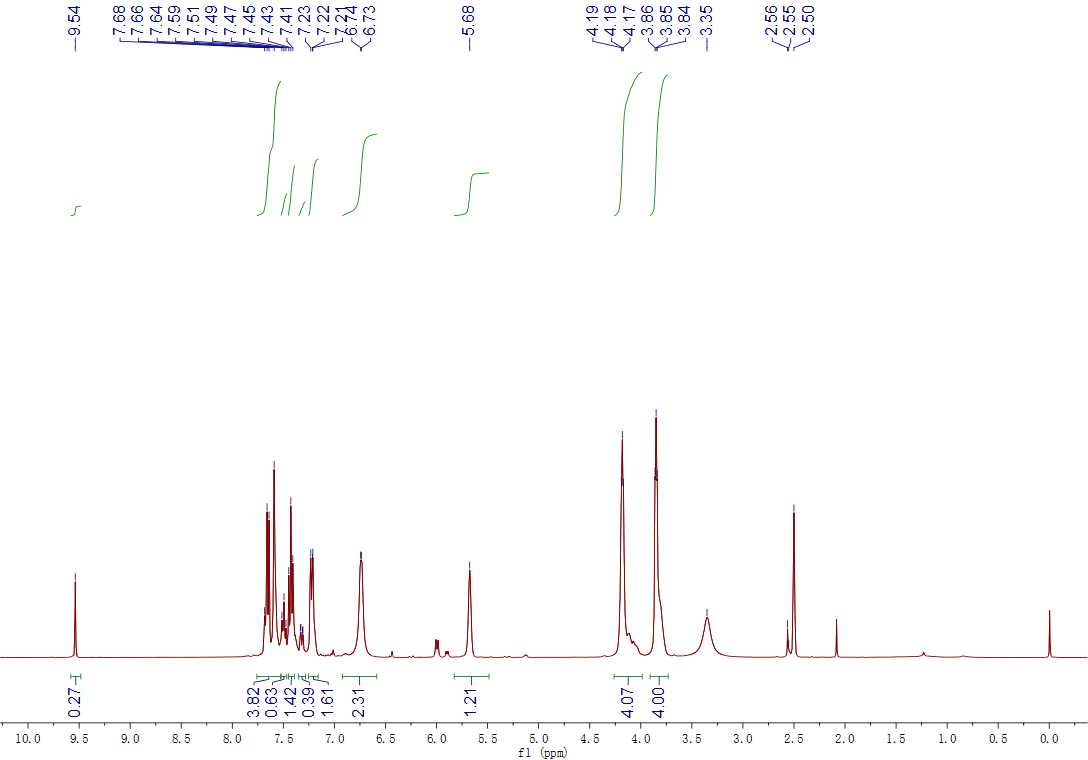


Supplementary Figure 95. **^1^H NMR spectrum of meta-ArGO-2 (400 MHz, (CD_3_)_2_SO)**


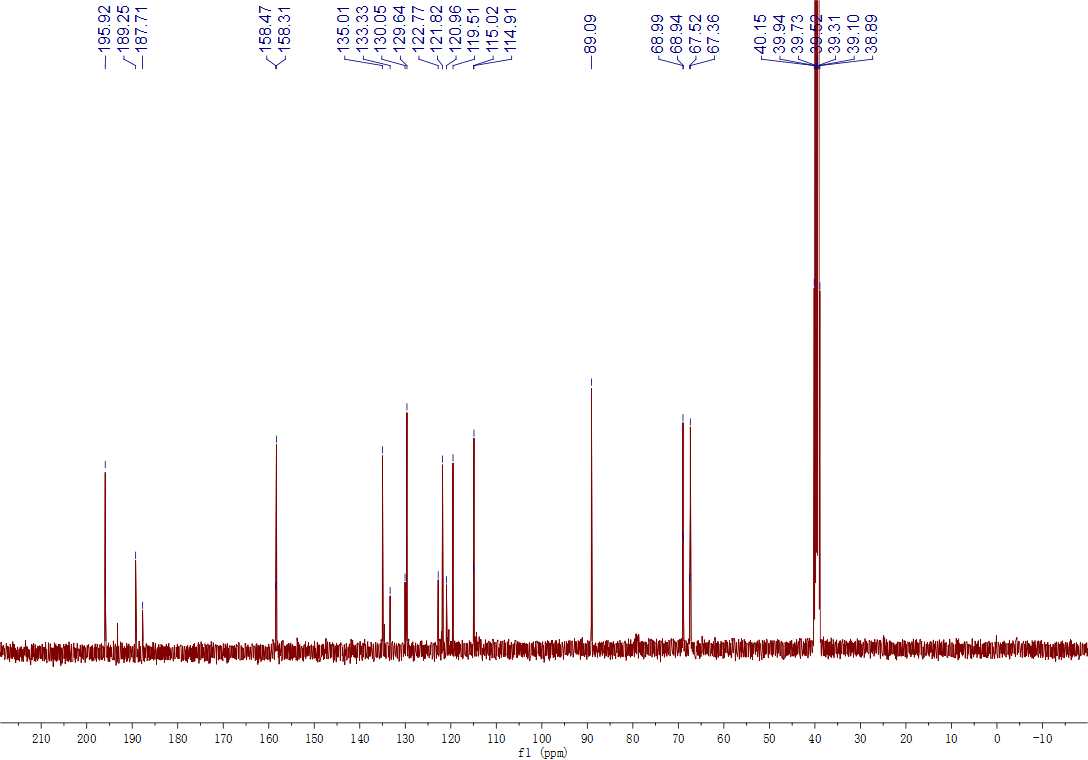


Supplementary Figure 96. **^13^C NMR spectrum of meta-ArGO-2 (100 MHz, (CD_3_)_2_SO)**


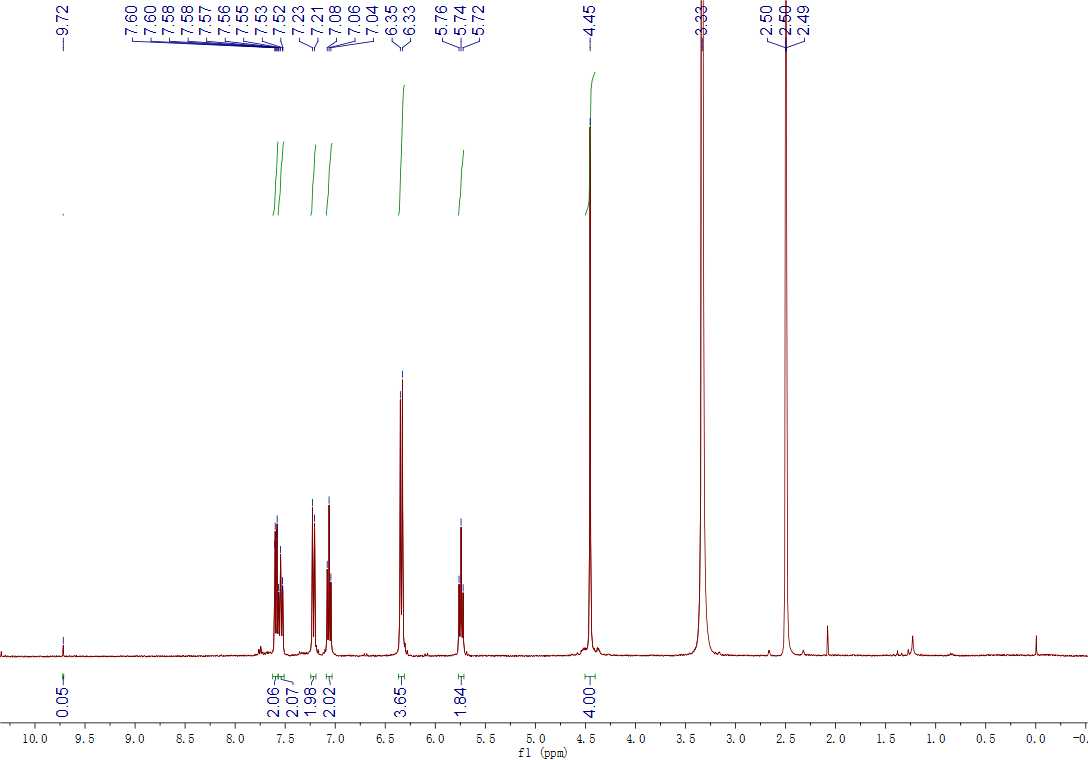


Supplementary Figure 97. **^1^H NMR spectrum of ortho-ArGO-1 (400 MHz, (CD_3_)_2_SO)**


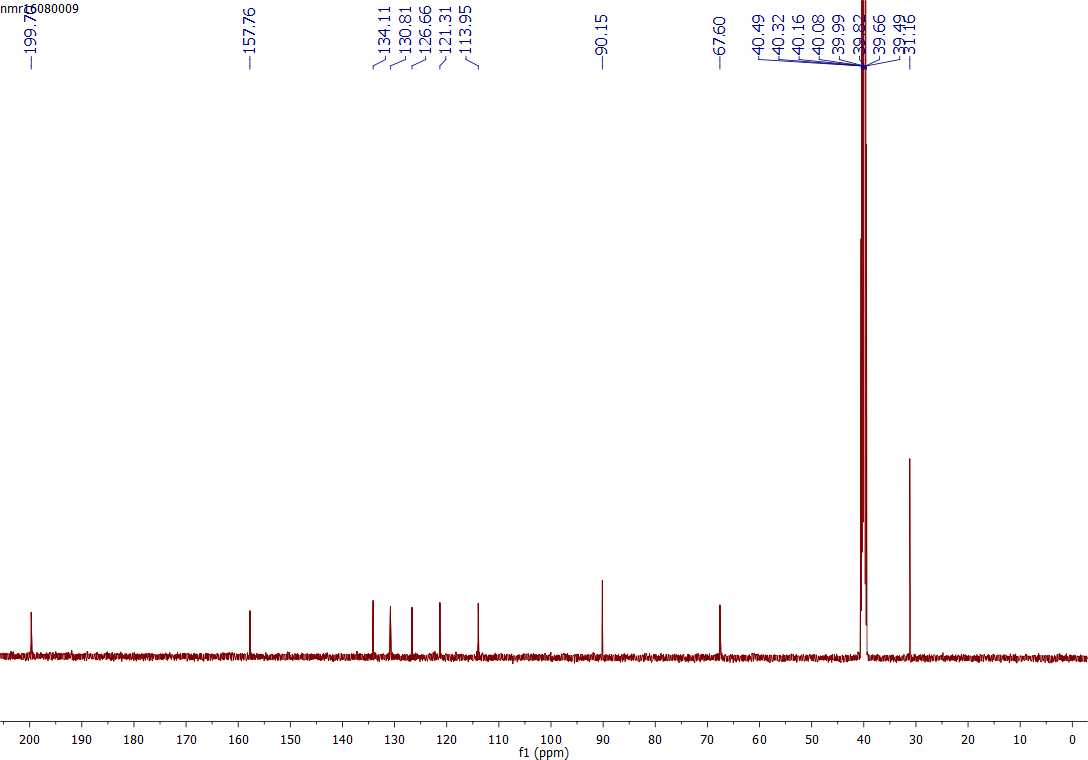


Supplementary Figure 98. **^13^C NMR spectrum of ortho-ArGO-1 (125 MHz, (CD_3_)_2_SO)**

^^
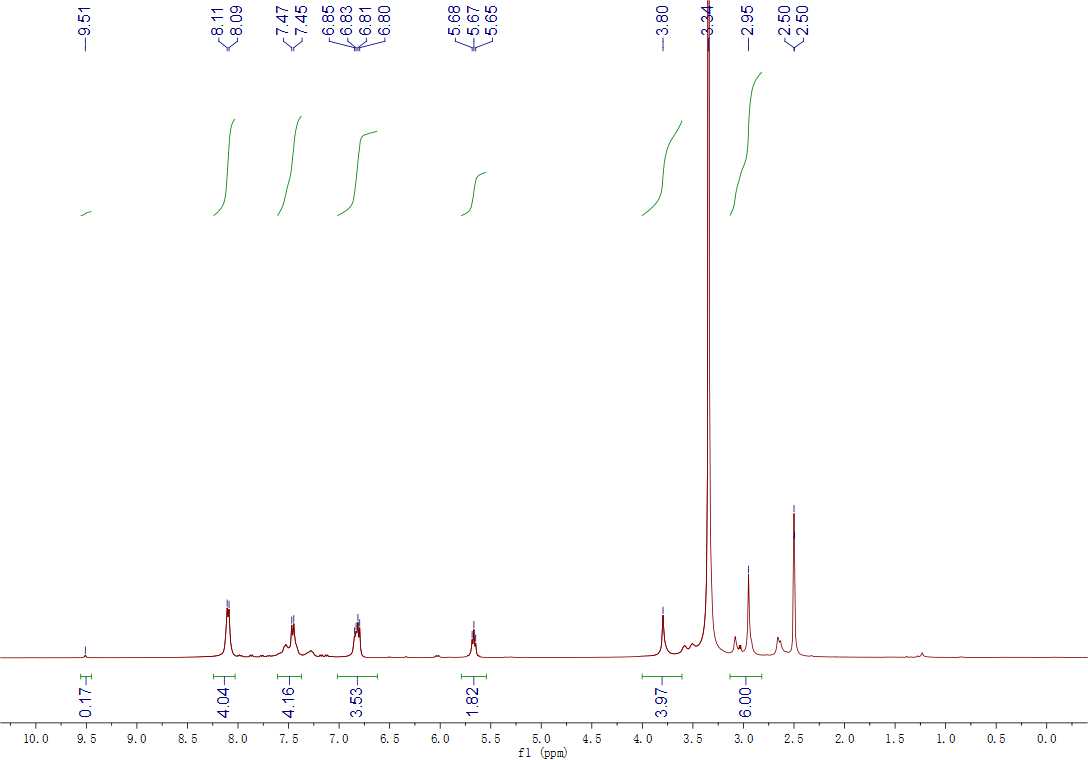


Supplementary Figure 99. **^1^H NMR spectrum of Amide-ArGO-1 (500 MHz, (CD_3_)_2_SO)**


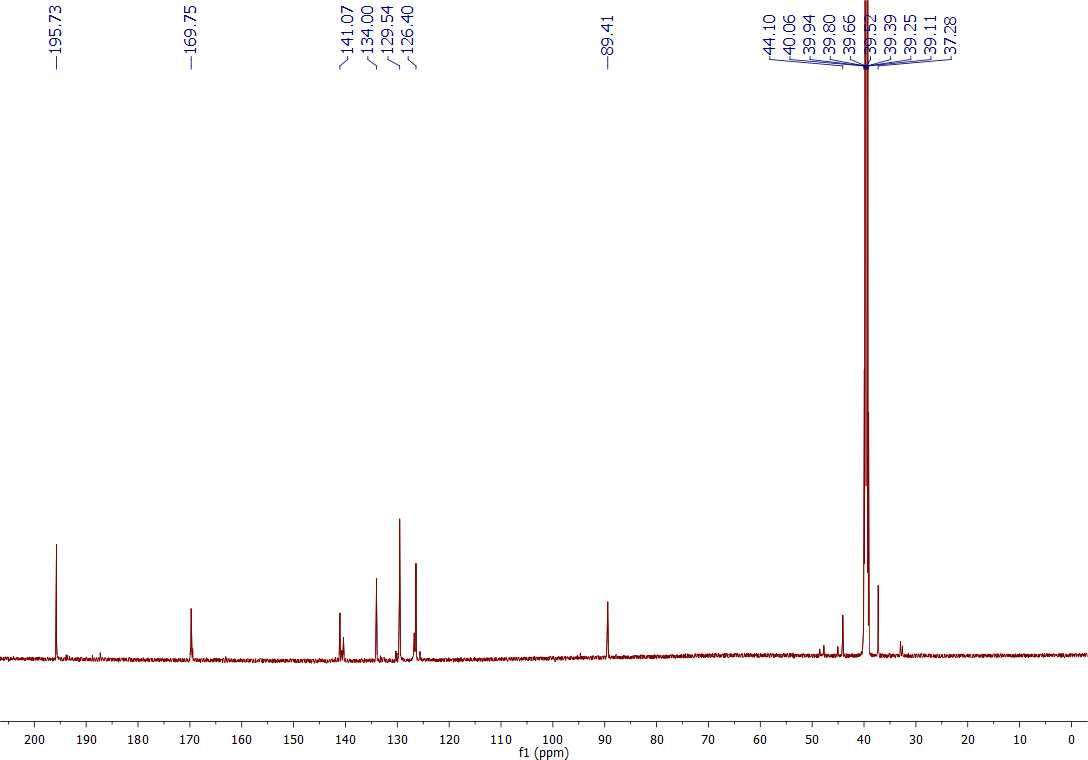


Supplementary Figure 100. **^13^C NMR spectrum of Amide-ArGO-1 (125 MHz, (CD_3_)_2_SO)**


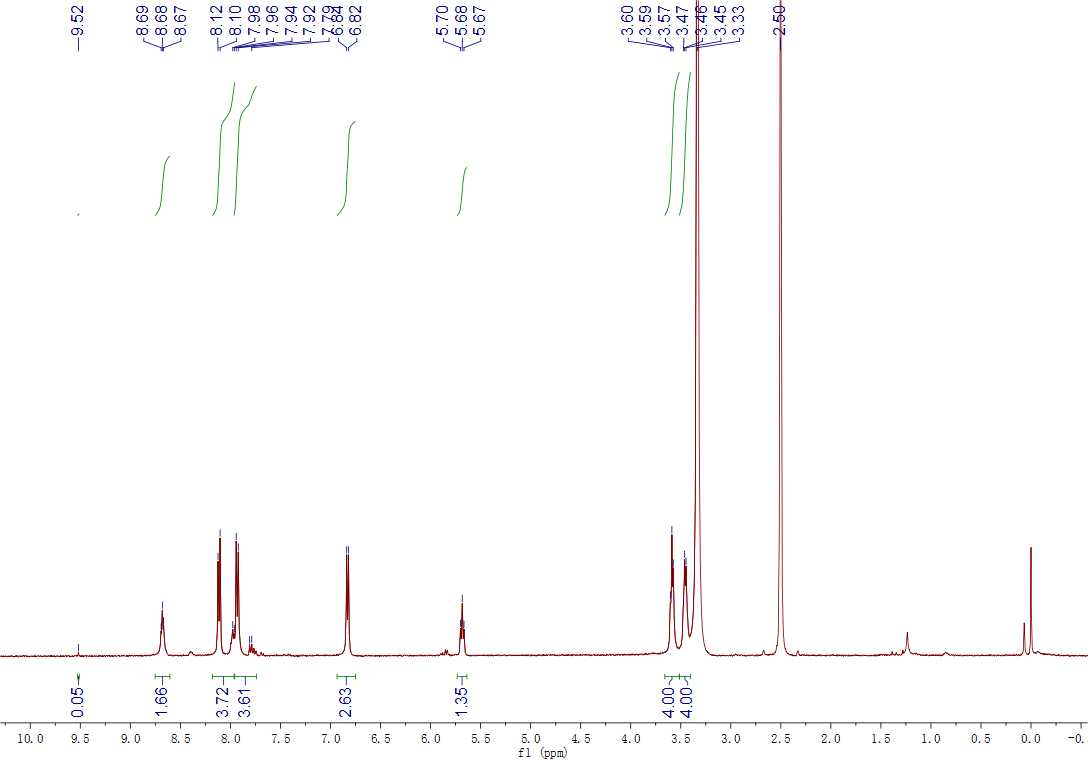


Supplementary Figure 101. **^1^H NMR spectrum of Amide-ArGO-2 (400 MHz, (CD_3_)_2_SO)**


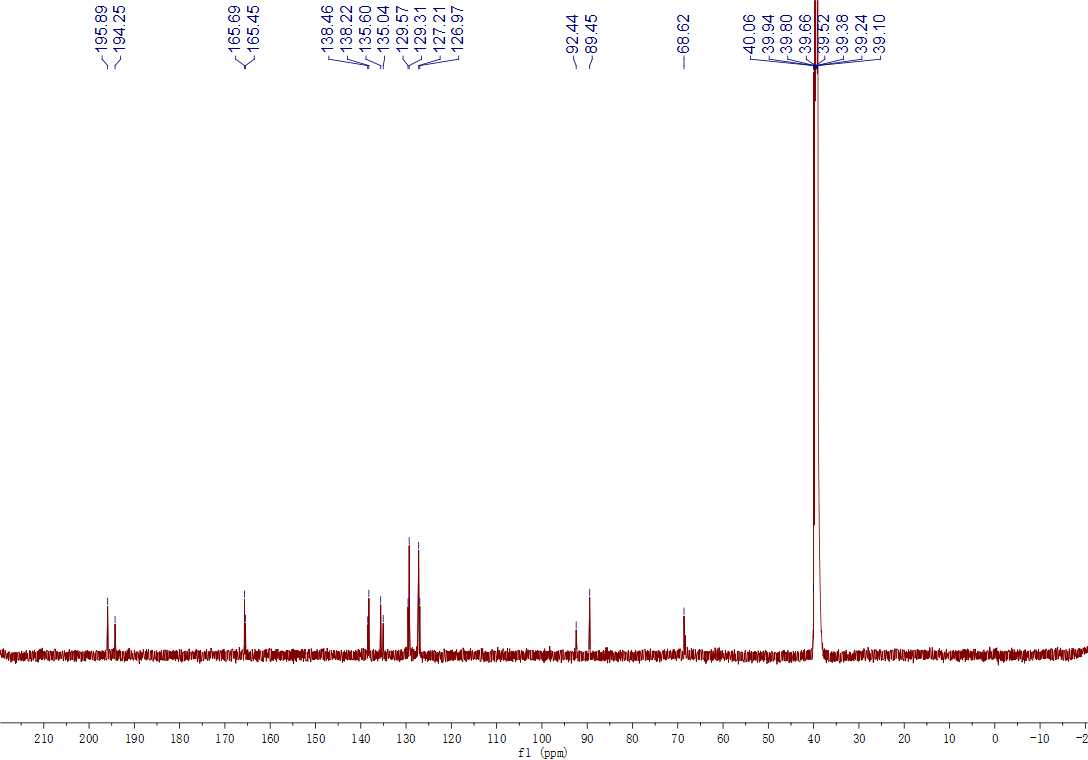


Supplementary Figure 102. **^13^C NMR spectrum of Amide-ArGO-2 (100 MHz, (CD_3_)_2_SO)**

# Supplementary Methods

## Mass Spectrometry Analysis

**Xwalk calculation of cross-linkable inter-molecular K-K, K-R or R-R pairs (Supplementary Figure 1)**

The cross-linkable inter-molecular pairs of 1808 protein complexes were calculated using following command:

java -Xmx1024m Xwalk -infile input_file -out output_file -aa1 LYS -aa2 ARG -a1 CA -a2 CA -max 60 -bb -homo -inter -euc

**Optimization of Reaction time and Temperature (Supplementary Figure 5a, b)**

12 µg BSA (0.6 µg/µl) was cross-linked with 1.5 mM ArGO3 in 100 mM, pH 8.0 borate buffer at room temperature (RT) or 37ºC for 15 min, 30 min, 60 min, or 90 min.

**Optimization of borate concentration (Supplementary Figure 5b)**

12 µg BSA (0.6 µg/µl) was cross-linked with 3 mM ArGO3 at RT for 1 h in 10, 50, 100, or 200 mM borate buffer at pH 7.5.

**Optimization of ArGO concentration (Supplementary Figure 5c)**

Six different concentrations of ArGO2 from 0.25 mM to 5 mM were tested further in 1 h room-temperature reactions each containing 12 µg BSA (0.6 µg/µl) in 50 mM, pH 7.5 borate buffer.

**SDS-PAGE analysis of the cross-linked proteins (Supplementary Figure 6)**

BSA, GST, Aldolase, PUD-1/2 and lysozyme were treated with ArGO1-3 at0.25, 0.5 or 1 mM for 1 h at RT. Cross-linked samples were separated by SDS-PAGE and stained with Coomassie Brilliant Blue. The uncropped gel figures are provided in Source Data file.

**Optimized KArGO Protein Cross-Linking Reaction Conditions**

12 μg protein (0.6 μg/μl) was cross-linked by 0.1/0.2 mM KArGO in a buffer mixture of 50 mM borate, 50 mM HEPES (pH 7.5) at RT for 15 min. The reaction was quenched using 5X volumes of acetone for at least 30 minutes at -20 ⁰C to precipitate the protein.

**Stability test of KArGO cross-links (Supplementary Figure 12e)**

A mixture of BSA and Aldolase, 7.5 μg each, was cross-linked with 0.1 mM or 0.2 mM KArGO. Cross-linked proteins were precipitated by six volumes of acetone, and the resulting pellets were left at RT, 4 ⁰C, or -20 ⁰C for 1-7 days before they were stored to -80 ⁰C till all the samples were collected for analysis. After digestion with trypsin or trypsin plus Asp-N, the samples were analysed by LC-MS/MS.

## Chemistry Experimental

**Reagents**

Chemical reagents were from J&K, Alfa Aesar or TCI Chemicals. Borate buffer was prepared from an aqueous solution of boric acid, and the pH was controlled by addition of 1 M NaOH. *N*-acetyl arginine methyl ester was synthesised according to literature procedures. 2,2’-Diiodoethyl ether and 1,2-bis-(2-iodoethoxy)ethane and were prepared from the alcohols using PPh_3_ and I_2_.^4^ 1,2-Dibromoethane was fractionally distilled from CaCl_2_.

**Solvents**

All reactions were carried out using anhydrous solvents unless explicitly stated. 1,4-dioxane was distilled from CaH_2_. Acetone was distilled from anhydrous CaSO_4_. CH_2_Cl_2_, Et_2_O, THF, toluene and DMF were dried by passing through a column of alumina.

**Experimental Techniques**

Etherification reactions were carried out in a sealed flask under an Argon atmosphere. SeO_2_ oxidation reactions were performed in sealed 10 mL microwave vials. All other reactions were carried out in oven-dried glassware under an argon atmosphere. Air and moisture sensitive reagents were transferred by syringe or cannula. Reaction temperatures other than room temperature were recorded as drysyn heating block temperatures. Microwave reactions were carried out in a Biotage microwave reactor**.** Brine refers to a saturated aqueous solution of NaCl. Analytical thin layer chromatography (TLC) was performed on Kieselgel 60 F254 pre-coated glass-backed plates and visualised with UV light (254 nm) or staining with potassium permanganate or vanillin solutions. Flash chromatography was performed using 200-400 mesh silica gel. Reverse-phase C18 chromatography was performed with a 4.5 g SepaFlash spherical C18-functionalised silica (particle size 30-50 μm) cartridge from Santai Technologies, using the CombiFlash R_f_+ chromatography system from Teledyne Isco. Water and acetonitrile were used as the eluents. Yields refer to chromatographically and spectroscopically pure materials, unless otherwise stated.

**Analytical UPLC-MS**

Samples were analysed by UPLC/MS on a Waters Auto Purification LC/MS system (3100 Mass Detector, 2545 Binary Gradient Module, 2767 Sample Manager, and 2998 Photodiode Array (PDA) Detector). The system was equipped with a Waters C18 5μm SunFire separation column (150*4.6 mm), equilibrated with HPLC grade water (solvent A) and HPLC grade acetonitrile (solvent B) with a flow rate of 0.3 mL/min.

**UV-Vis reaction monitoring**

Reaction was monitored using the Thermo-Fisher Nano-drop UV spectrometer (see Supplementary Figure 3 for experimental details).

**Characterisation**

Melting points are uncorrected. Fourier transform infra-red (IR) spectra were recorded neat using a Thermo Fisher FT-IR200 spectrophotometer. ^1^H, ^13^C and ^19^F NMR spectra were recorded on Bruker DRX-400, Bruker AV-400 or AV-500 spectrometers. Chemical shifts (d) are expressed in parts per million (ppm) relative to the residual solvent peak. Abbreviations are: s, singlet; d, doublet; t, triplet; q, quartet; m, multiplet. High-resolution mass spectra were obtained at Peking University Mass Spectrometry Laboratory using a Bruker APEX instrument.

# Supplementary References

1. Kahraman A, Malmstrom L, Aebersold R. Xwalk: computing and visualizing distances in cross-linking experiments. *Bioinformatics* **27**, 2163-2164 (2011).

2. Zhang Q, Crosland E, Fabris D. Nested Arg-specific bifunctional crosslinkers for MS-based structural analysis of proteins and protein assemblies. *Anal Chim Acta* **627**, 117-128 (2008).

3. Young RM, Davies-Coleman MT. Microwave-assisted selenium dioxide oxidation of aryl methyl ketones to aryl glyoxals. *Tetrahedron Lett* **52**, 4036-4038 (2011).

4. Cernigliaro G. *Pheromone Synthesis 4. A Synthesis of (+)-methyl-n-tetradeca-trans-2,4,5-trienoate, an allenic ester produced by the male dried bean beetle Acanthoscelides obtectus (Say); P.J. Kocienski, G.J. Cernigliaro, G. Feldstein, J. Org. Chem., 42:2, 353-55, (1977)* (1977).
