# Supplementary material for: Improving mass spectrometry analysis of protein structures with arginine-selective chemical cross-linkers
Source: Nat Commun. 2019 Sep 2;10:3911. doi: 10.1038/s41467-019-11917-z (PMC6718413; doi:10.1038/s41467-019-11917-z)
Supplement: Supplementary file 3 — Description of Additional Supplementary Files [file 41467_2019_11917_MOESM3_ESM.docx]

**Description of Supplementary Files**

**File Name:** Supplementary Data 1

**Description:** All ArGO cross-links identified under the optimal conditions. Sheet 1: ArGO2 cross-links from six model proteins. (ArGO2 6Protein) Sheet 2: ArGO1 and ArGO2 cross-links from the CNGP complex. (CNGP_ArGO).

**File Name:** Supplementary Data 2

**Description:** KArGO cross-links identified from six model proteins.
